# Born captive: A survey of the lion breeding, keeping and hunting industries in South Africa

**DOI:** 10.1371/journal.pone.0217409

**Published:** 2019-05-28

**Authors:** Vivienne L. Williams, Michael J. ‘t Sas-Rolfes

**Affiliations:** 1 School of Animal, Plant & Environmental Sciences; University of the Witwatersrand, Wits, South Africa; 2 Oxford Martin Programme on the Illegal Wildlife Trade, University of Oxford, Oxford, United Kingdom; Université de Sherbrooke, CANADA

## Abstract

Commercial captive breeding and trade in body parts of threatened wild carnivores is an issue of significant concern to conservation scientists and policy-makers. Following a 2016 decision by Parties to the Convention on International Trade in Endangered Species of Wild Fauna and Flora, South Africa must establish an annual export quota for lion skeletons from captive sources, such that threats to wild lions are mitigated. As input to the quota-setting process, South Africa’s Scientific Authority initiated interdisciplinary collaborative research on the captive lion industry and its potential links to wild lion conservation. A National Captive Lion Survey was conducted as one of the inputs to this research; the survey was launched in August 2017 and completed in May 2018. The structured semi-quantitative questionnaire elicited 117 usable responses, representing a substantial proportion of the industry. The survey results clearly illustrate the impact of a USA suspension on trophy imports from captive-bred South African lions, which affected 82% of respondents and economically destabilised the industry. Respondents are adapting in various ways, with many euthanizing lions and becoming increasingly reliant on income from skeleton export sales. With rising consumer demand for lion body parts, notably skulls, the export quota presents a further challenge to the industry, regulators and conservationists alike, with 52% of respondents indicating they would adapt by seeking ‘alternative markets’ for lion bones if the export quota allocation restricted their business. Recognizing that trade policy toward large carnivores represents a ‘wicked problem’, we anticipate that these results will inform future deliberations, which must nonetheless also be informed by challenging inclusive engagements with all relevant stakeholders.

## Introduction

The African lion is currently the only big cat of the genus *Panthera* for which international commercial trade is legal under the Convention on International Trade in Endangered Species of Wild Fauna and Flora (CITES) [1]. In response to emerging market demands for lion products, including viewing tourism, cub petting, trophy hunts and body parts, entrepreneurs in South Africa have developed a substantial commercial captive lion breeding industry, reaching a scale similar to that of captive tiger breeding operations in China [2,3]. As with China’s so-called ‘tiger farms’, the role of such commercial breeding operations is debated, with critics arguing that their presence has no conservation value [4] and, at least in the case of tigers, may even constitute a threat to wild populations [5]. However, the exact relationship between captive and wild lion populations remains evidentially unclear, and it is also plausible that the former may provide a buffer effect against over-exploitation of the latter [6]. This relationship warrants further investigation, especially given increasingly vocal public opposition to commercial captive lion breeding and some recent consequential trade policy shifts.

At the 17^th^ CITES Conference of the Parties (CoP17) in 2016, debates on the trade in lion body parts intensified when consensus could not be reached on a proposal by nine predominantly western African countries to transfer all African populations of *Panthera leo* (African Lion) from Appendix II to Appendix I of CITES [1,7–9]. Several southern African countries in particular rejected the proposal [1,7–9]. Instead, through negotiations within a working group, a compromise to retain *P*. *leo* on Appendix II was agreed with the following annotation: *A zero annual export quota is established for specimens of bones*, *bone pieces*, *bone products*, *claws*, *skeletons*, *skulls and teeth removed from the wild and traded for commercial purposes*. *Annual export quotas for trade in bones*, *bone pieces*, *bone products*, *claws*, *skeletons*, *skulls and teeth for commercial purposes*, *derived from captive breeding operations in South Africa*, *will be established and communicated annually to the CITES Secretariat* [10].

In accordance with the annotation, South Africa must establish an annual export quota for captive-origin lion body parts. The South African CITES Scientific Authority, based at the South African Biodiversity Institute (SANBI), advises the South African Department of Environmental Affairs (DEA) on the proposed size of this quota (although the Ministry of Environmental Affairs makes the ultimate decision). In South Africa, conservation regulation is a competence shared between the national government and nine provincial agencies, the latter of which may have significantly varying laws and consequent lion management practices. Accordingly, the Scientific Authority draws upon these provincial agencies for input.

In 2017, the Scientific Authority initiated a programme of interdisciplinary, collaborative, scientific research with university institution affiliates (including ourselves) to obtain further information on lions bred and maintained in captivity (hereafter referred to as ‘the captive lion industry’), legal and illegal trade, and consequences of trade for wild lions; this information is assimilated and used in policy recommendations to the DEA. As part of this research, we initiated the ‘National Captive Lion Survey’ of privately-owned properties with lions held captive for various core purposes, *viz*.: breeding, hunting, and ‘keeping’ (*i*.*e*. the retention and rearing of lions for personal pleasure, display, rehabilitation, security, and potential future sales of live animals and body parts).

The survey was run from August 2017 to May 2018 and was intended to establish baseline information for understanding the essential factors and dynamics at the interface between captive lions and wild lion conservation. The stated core aims were to: (i) understand the captive lion breeding, keeping, and hunting industries, and the trade in lions (live animals, bones and other products); (ii) investigate the trade in captive-produced lion skeletons under the newly implemented quota system; (iii) gain an understanding of the consequences of trade bans/suspensions on imports of captive-origin trophies on both the captive lion industry and wild lions; and (iv) strengthen the evidence base for the annual review of the lion skeleton export quota. Given the variability of purpose and practice within the captive lion industry, not all the aims were relatable to all responding facilities (especially those that do not sell live lions and products or allow hunting on the premises). However, given the ability of facilities to exchange live lion stocks, we sought to gather as much information as possible across the entire industry, including relevant differences in practice between provincial jurisdictions that may have arisen as a consequence of their regulatory disparities.

Our approach was grounded in two key propositions. The first is that South Africa’s captive lion industry constitutes part of a complex adaptive social-ecological system, which includes and potentially affects *in situ* populations of lions and other large felids, via trade and other mechanisms that remain poorly understood [11,12]. Second is that the most appropriate way for the Scientific Authority to address such uncertainty is by employing principles of adaptive management, informed by an ongoing process of scientific research [13]. We further postulate that, in respect of commercial activities related to lions, both legal and illegal actors are substantially motivated by economic incentives. This is consistent with past similar analyses related to tigers [14] and highlights the importance of establishing indicative economic data such as trends in sales volumes, stock management and market prices, as these will drive future adaptive market activity.

**Fig 1:**
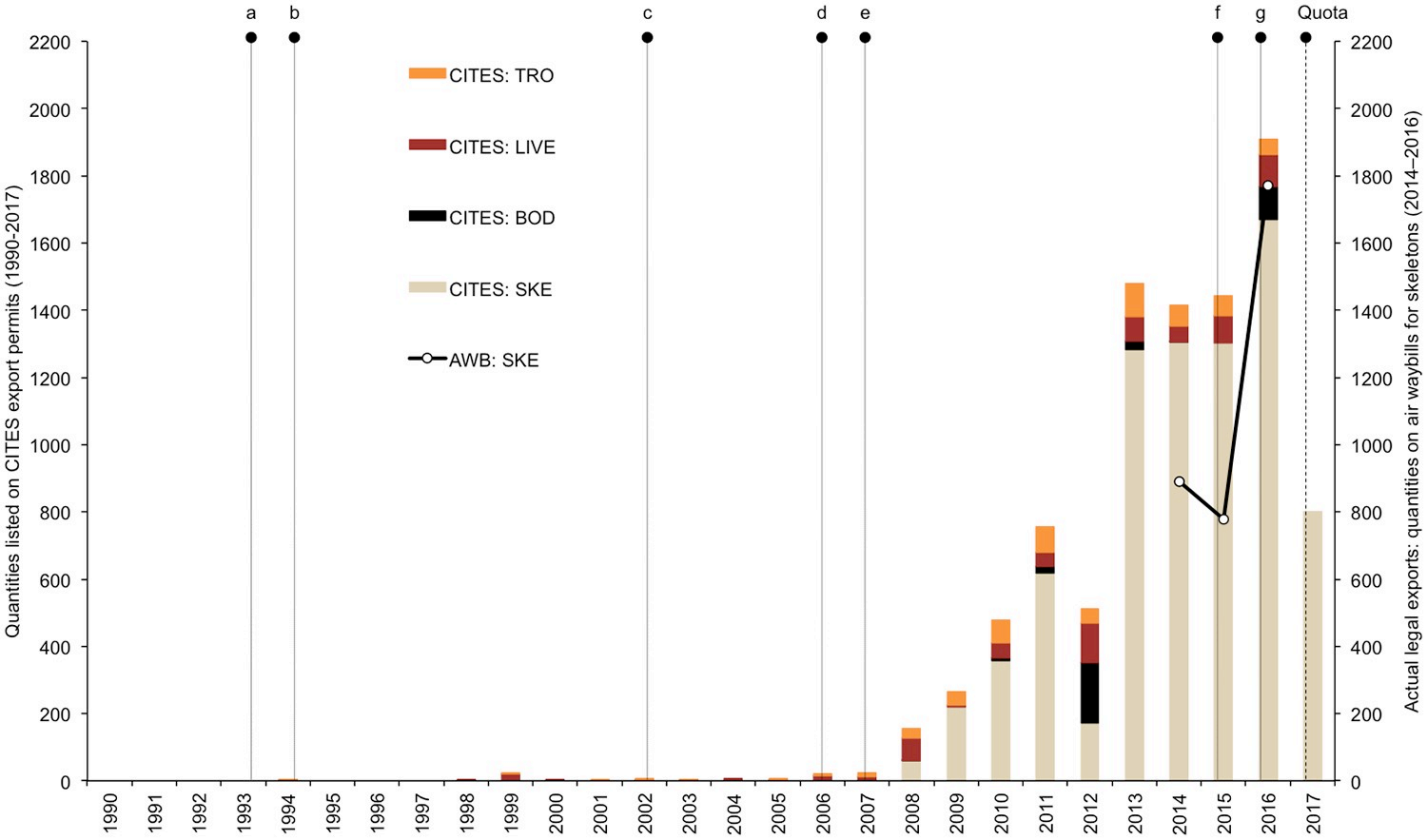
Policy interventions and annual exports of lion products from South Africa to East-Southeast Asia. Lettered vertical lines indicate the years that measures (policy interventions and regulations) aimed at protecting tigers, other Asian big cats and/or lions were introduced or implemented. a = 1993: Chinese ban on the domestic trade in tiger bone; b = 1994: adoption of CITES Res. Conf. 9.13 (CoP9); c = 2002: adoption of CITES Res. Conf. 12.5 (CoP12); d = 2006: China bans leopard hunting and purchase of leopard bones; e = 2007: CITES (CoP14) Decision 14.69 against tiger farming and breeding for trade in their parts and products; f = 2015: introduction of lion trophy import bans (Australia and France, in March and November respectively), and the USA lists lions in southern and eastern Africa as threatened under the Endangered Species Act (ESA) in December; g = 2016: USA import suspension of trophies from captive-bred lions implemented from 22 January, and a lion trophy import ban by the Netherlands in May; CITES adoption of Appendix II annotation for African lion (CoP17) mandates SA to introduce a lion bone quota from 2017. Histogram legend: CITES permits issued for export of lion trophies (TRO), live lions (LIVE), bodies (BOD) and skeletons (SKE) from South Africa. Line graph: air waybill (AWB) records of actual skeleton exports from South Africa in 2014–2016 (see also [1]). *Note*: The actual annual legal exported quantities are less than what are listed on the issued CITES permits because traders do not typically export all of what they apply to export, and/or they do not use the permit in the same year it was issued.

Williams et al [1,2,15] questioned whether measures implemented between 1993 and 2007 to curb the Asia-driven tiger parts trade provoked the start of South Africa’s lion bone trade in 2008 (Fig 1), and hence whether well-meaning policy interventions intended to protect species can create economic incentives that perversely influence trade dynamics and lead to unintended consequences elsewhere. In conducting the National Captive Lion Survey, we similarly aimed to explore the impacts that trade bans, such as the United States of America’s (USA) suspension on imports of captive-origin lion trophies from early 2016, might have on the hunting industry and accordingly on captive lion breeding. Since American hunters comprise >50% of South Africa’s foreign hunting clients (this survey, [1,2]), and they imported >50% of the lion trophies originating from captive-born lions [16], we hypothesized that, subsequent to the trade suspension, the market for lion hunts would decline significantly and consequently induce some hunting and breeding facilities with a surplus of captive lions to reduce their stock numbers by euthanasia and sell these skeletons to Asia to recoup their loss of earnings [1]. The results of our survey confirmed this prediction.

Whereas an earlier survey of the captive lion industry investigated its economic significance [17], we sought more specific evidence on industry participant reactions to inter-provincial regulatory variability, changing policies, and changing market conditions, and how these might ultimately impact upon lion breeding, trade, and conservation. Following confirmation that disruption to the hunting market has significantly affected the industry, we questioned what strategies industry participants would employ in response to consequent losses of income. We also sought insight into the added impacts of the instituted skeleton export quota, which in 2017 amounted to an effective contraction to less than half the number actually exported in 2016 (Fig 1). The results of the survey are intended to inform future policy deliberations rather than be prescriptive, although they do highlight risk areas that warrant attention if threats to wild lions are to be adequately mitigated.

## Methods

### Questionnaire survey

The online ‘National Captive Lion Survey’ (Version 1) was launched in August 2017. The structured semi-quantitative questionnaire was designed and pre-tested over a four-month period, and initially had 61 questions in seven Sections, namely: (A) participant information; (B) facility information; (C) captive stock numbers; (D) lion breeding; (E) live lion trade; (F) trade in lion bones, skeletons, products and derivatives; and (G) lion hunting (S1 File). The questions were designed to also capture information not specifically relevant to the trade in lion derivatives, and thus assemble information on the keeping of non-wild lions by facilities with varying purposes. ‘Skip logic’ was applied to the questionnaire to ensure that respondents were directed to Sections and questions applicable only to activities conducted on their properties; accordingly, the number of facilities engaged in these activities determined the sample sizes per Section and question. Assistance with the appropriate industry-specific wording of selected questions was sought from the South African Predator Association (SAPA).

The survey was administered using SurveyMonkey (www.surveymonkey.com), and was offered in English and Afrikaans. The questionnaire was distributed via email invitation among potentially suitable research participants at breeding, keeping (including sanctuaries, zoos and predator parks) and/or hunting facilities identified by DEA, SANBI, and SAPA (to its members). A hard copy version, administered by SAPA, was also available for facilities unable or unwilling to complete the survey online. A key purpose of the survey, as stated in the research collaboration memorandum, i.e. to *‘strengthen the evidence base for the annual review of the lion bone quota to ensure it is sustainable and not detrimental to wild lion populations’*, was declared in the survey introduction to encourage participation by stakeholders in the lion bone trade sector.

Version 1 of the survey ran for 140 days until December 2017; however, of the 126 visitors to the site, only 35 respondents (28%) answered >1 of the six sections [18]. The respondents represented 11% of the ‘ToPS’-registered lion facilities in South Africa. (*Note*: because lions are vulnerable in terms of the *South African National Environmental Management and Biodiversity Act* No. 10 of 2004, facilities carrying out any of seven restricted activities, such as lion keeping, require permits regulated by the *Threatened or Protected Species* (ToPS) regulations of 2007). Although results from Version 1 provided some useful baseline data, the response rate was unsatisfactory and the results inadequate for robust scientific scrutiny. Hence, the survey was reopened in February 2018 to increase the sample size until ≥25% of the ToPS-registered lion breeding/hunting facilities per province had been sampled. To account for the survey being rerun in 2018, the Version 1 questionnaire was updated with appropriate revisions. SAPA was re-engaged to distribute the revised questionnaire to its members, and the survey was also circulated to several lion bone traders, PAAZA (Pan African Association of Zoos and Aquaria) and PHASA (Professional Hunters Association of South Africa). Version 2 ran for 94 days until May 2018; there were 120 visitors to the site, of which 82 respondents (68%) answered >1 of the six sections. In total, the National Captive Lion Survey (Versions 1 & 2) ran for 234 days (33.3 weeks). Of the 117 respondents, 23 did not use the online instrument; hard copies of these responses were completed with the assistance of a third-party collector from SAPA.

Twelve compulsory questions were designed to gain an overall impression of the scale and nature of the industry, with additional voluntary questions aimed at gaining more detailed information on aspects such as the evolution of markets for body parts and hunting. Quantitative and qualitative data were sought to understand the demographic and economic aspects of the industry and drivers of decision-making. Given the sensitive nature of some data and possible uncertainties about specific numbers, certain questions enabled respondents to choose options within ranges rather than fill in exact amounts. Appropriate values for these ranges were determined through consultation with SAPA. The final assemblage of data was expected to provide an overview of the economic geography and trends of the captive lion industry rather than an accurate census with specific economic time series data. For the purposes of the intended analysis, achieving this level of detail was considered realistic and sufficient.

All protocols were carried out in accordance with the ethical guidelines of the Human Research Ethics Committee (non-medical) of Wits University (Protocol Number H17/06/55). Respondents were informed on the first page of the questionnaire that they could remain anonymous (to encourage answers to questions on sensitive issues such as income to be more accurate) and that submission of the online questionnaire would be interpreted as their consent for the information they provided to be used. The researchers did not interview any of the respondents in person. Despite some respondents identifying themselves, confidentiality is maintained throughout this paper. Furthermore, with the exception of the 23 respondents who completed the survey via the assistance of a third party from SAPA, only the first author has access to the survey responses and the names of the participants who chose to identify themselves.

### Data analysis

In addition to a synthesis of the overall result for each question, most results were subdivided according to the primary purpose of the facility (e.g. breeding or hunting), and/or the province (to elucidate inter-provincial variances shaped by legislative differences). Delimiting the primary purpose was mostly based on answers to Question 11 (ranked core purpose), but the consistency of answers to all 63 questions was also cross-checked (to see if the answers were aligned) and all activities recorded as taking place on a property were ascribed to a respondent. Accordingly, facilities were noted as being either single-purpose (e.g. breeding only) or multi-purpose (e.g. breeding and hunting). Whenever breeding *and* hunting took place on a property, the answers to all the questions were scrutinised to assess whether, of the two purposes, the primary purpose was lion breeding or hunting. Hence, results reported as ‘mostly breeding’ or ‘both*B’ refer to properties where the primary purpose is lion breeding even though hunting takes place; similarly, ‘mostly hunting’ or ‘both*H’ refers to properties where the primary purpose is lion hunting even though breeding takes place. This purpose-based approach was taken to especially elucidate differences in the activities and adaptations of facilities in the breeding and hunting industries.

Data were mostly analysed according to the percentage of responses per category per question. Since several questions required multiple responses, the total response percentages for some questions exceed 100%. Furthermore, since response rates to questions varied (in part due to questions not being applicable to every respondent), it was often more appropriate to calculate a mean and standard deviation (SD) rather than a total (e.g. mean loss of earnings per farm, rather than the total loss for all farms) so that comparisons of results for questions of unequal sample sizes could be made. Potentially anomalous responses on price values were compared to the mean ± 1*SD; outliers less than or greater than the mean ± 3*SD were excluded. Given the aggregation of certain economic data, monetary values relating to income and prices were analysed in terms of mean trends over time, the results of which are considered sufficiently revealing for the intended analysis. Prices were collected from respondents in ZAR, the local South African currency, and are reflected as nominal values (i.e. they are not adjusted for inflation). For the benefit of readers, figures and tables in S1 Figs and S1 Tables display figures converted to US dollars; however, given that exchange rates fluctuate, and breeders typically base their decisions on ZAR values, all figures and tables in the main text display these as such.

## The National Captive Lion Survey results

This section (and accompanying supplementary files) provides a comprehensive account of the survey results, which include both quantitative and qualitative data across the different subsectors of the captive lion industry (i.e. breeding, keeping, hunting, and body part sales). Readers are invited to focus on categories of specific interest, skip those of lesser interest, and proceed to the Discussion section.

### Facility information: Background and context

The National Captive Lion Survey yielded 117 usable responses, mostly from the Free State province (40%, Table 1) (S2 File lists the number of responses per question and where to find each result in this publication). The respondents represented 36% of the ToPS registered lion facilities in South Africa (Table 1). Data on the numbers of lions in these facilities (see section on ‘Captive lion numbers’) indicate a sample bias toward larger breeding facilities, given that respondents appear to have accounted for at least 68% of the total number of captive lions recorded in the 2016 ToPS census.

**Table 1:** Number and proportion of survey respondents by province and in relation to the total number of registered ToPS (Threatened or Protected Species) breeding and hunting facilities per province.

Province	No. of respondents answering all/some of the questionnaire (n = 117)	% allocation of responses by province	No. of ToPS registered breeding and/or hunting facilities per province ^a^	% responses in relation to the number of ToPS registered breeding/hunting facilities per province
**Free State (FS)**	47	40%	161 ^b^	29%
**North West (NW)**	45	38%	104	43%
**Limpopo (LP)**	16	14%	30	53%
**Gauteng (GP)**	5	4%	15	33%
**Eastern Cape (EC)**	3	3%	9	33%
**Western Cape (WC)**	1	1%	-	- ^c^
**KwaZulu-Natal (KZN)**	0	-	2	0% ^d^
**Mpumalanga (MP)**	0	-	-	-
**Northern Cape (NC)**	0	-	-	-
**Total**	117		321	36%

^a^ Correct to late 2016. Excludes facilities that keep and/or display lions only. As of the end of 2016, there were 297 breeding facilities with altogether >7800 lions (including cubs), and 24 hunting facilities with ±637 adult lions released into camps in 2016 for hunting in 2016/2017 (Table A in S1 Tables). *Source*: Department of Environmental Affairs.

^b^ Data supplied excluded the number of hunting facilities in the Free State, which should be <5.

^c^ No breeding and/or hunting facilities in the Western Cape. The respondent from the Western Cape had a property on which lions were kept and displayed.

^d^ A facility in KZN tried twice to respond and could not proceed for unresolved technical reasons.

Most respondents remained anonymous (53%) or partially anonymous (20%; persons identified themselves to a third-party collector with a hard copy of the survey). The persons answering the questionnaire were typically the facility owners or their relatives (89%), and 11% were employees. Respondents were mostly members of SAPA (69%) and/or PHASA (19%) (Table B in S1 Tables), and thus mostly breeders and/or keepers of captive-born lions. The facilities opened between 1982 and 2017 (n = 105), 51% of them in the 10 years from 2008 (Figure A in S1 Figs) and employed between one and 120 people (mean = 13±18; mode = 5) (Figure B in S1 Figs). While some respondents enumerated all their employees, others counted only those employed to work with lions if they conducted mixed farming activities on their premises.

Captive lions were mostly held at facilities for the broad purposes of breeding, keeping, hunting, and/or display. Only breeding facilities bred lions (i.e. keeping- or display-only facilities do not breed lions). Facilities that display lions are open to the public (e.g. a sanctuary, predator park, research/education or tourism purposes), whereas keeping facilities are not typically open to the public and lions are reared and kept for live and/or product sales and even ‘personal’ purposes. Overall, 88 facilities (75%) bred lions, 71 (61%) kept and/or displayed lions, and 33 (28%) facilities undertake hunting on the property. Eight respondents did not indicate a core purpose anywhere in the questionnaire. The facilities were mostly recorded as multi-purpose (n = 76, 65%), with 33 (28%) recorded as being single-purpose only (viz. 18 breeding-only, 11 hunting-only, 3 display only, 1 keeping only). Of the multi-purpose facilities, the dual purpose of breeding and keeping/display was the most common combined activity. When questioned further on the reasons for breeding and/or keeping lions, most facilities (63%–78%) did so for the purposes of live sales and hunting (Table C in S1 Tables; Figure C in S1 Figs). The breeding and keeping of lions for tourism and personal use/pleasure was listed as a purpose by ≤26% of facilities (Table C in S1 Tables).

**Fig 2:**
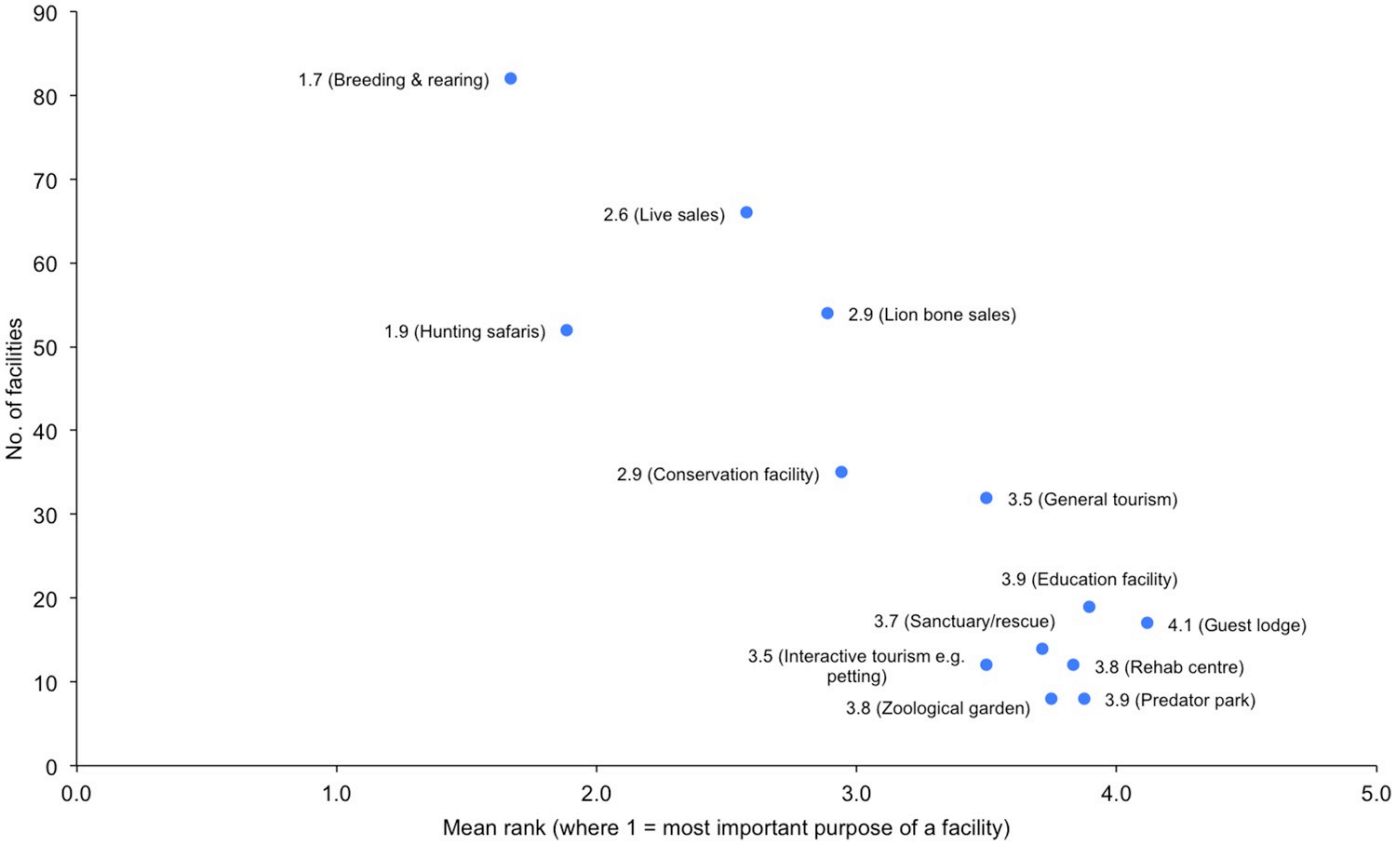
Core purposes of the facilities, expressed as the overall mean rank per purpose plotted against the number of facilities that ranked the purpose. Results correspond to Question 11. Respondents selected and ranked as many of the 13 pre-listed purpose categories that applied to their facilities.

Since facilities typically have multiple reasons for having lions, respondents were asked to rank their core activities from most- to least-important (where 1 = highest rank, down to rank 13 for the lowest placed of the pre-listed potential purposes). When the mean rank per purpose was calculated, ranks closer to 1 (left-hand side of x-axis) indicated purposes that were overall more important than purposes with lower mean ranks (i.e. values increasingly >1) (Fig 2). Breeding/rearing, and hunting safaris, were ranked highest as the core purpose of the facilities (mean ranks of 1.7 and 1.9, from 82 and 52 facilities, respectively); lion bone sales had the fourth highest overall mean rank (the same rank as a conservation facility), but was never listed as the highest ranked (principal) purpose. The least important facility purpose was ‘guest lodge’ (rank 4.1), and n = 8 ranked the purpose as either a predator park (rank 3.9) or zoological garden (rank 3.8) (Fig 2).

The specific reasons why facilities bred and/or kept lions, based on answers to Questions 9–11, are quantified in Table C in S1 Tables and Fig 2. However, when consolidating the respondents’ answers to all 63 questions, further information on links facilities have to specific activities pertaining to lions was captured, namely:

1. *Breeding*: 107 (91%) facilities have some active association with the captive lion breeding industry—either through breeding lions on the property, live sales, keeping, or hunting;
2. *Hunting*: 92 (79%) facilities had/have some association with the hunting industry in the past and/or present through either hunting on the property and/or breeding and live sales on different properties;
3. *Live sales*: 98 (84%) facilities have sold live lions for either breeding, keeping, hunting or display purposes in South Africa and/or internationally;
4. *Products*: 84 (72%) facilities have knowingly sold lion products of some description (trophies, derivatives, bones, or parts for traditional medicine) in the past or present (however, not all these facilities currently sell parts). Of these, 77 (66%) had/have sold skeletons in the past and/or present to South African lion bone traders;
5. *Sanctuaries*: 15 (13%) facilities consider one of their purposes to be that of a sanctuary to captive and/or wild origin lions;
6. *Rehabilitation*: 13 (11%) facilities consider one of their purposes to be that of rehabilitation for captive and/or wild origin lions;
7. *Conservation*: 35 (30%) of facilities consider conservation of lions to be a purpose for their facility;
8. *Tourism*: 53 (45%) said that tourism generated some income for the facility.

Of the 107 (91%) responding facilities that are directly linked to captive lion breeding (through trade of live lions), 88 facilities (75%) bred lions for various reasons (e.g. reasons in Table C in S1 Tables). Of these, 67 disclosed the number of adult male and female lions that are being used for breeding (where adults are lions ≥3 years old). On average, 64% of the breeding adults at a facility are female and 36% are male (Table D in S1 Tables), and this average is consistent across the provinces regardless of the size of the facilities. Facilities in the Free State generally have more breeding lionesses (mode = 20) than other provinces. In addition to selling and hunting, or deaths from fights and natural mortalities, lion numbers at breeding facilities are regulated and controlled in several ways (Table E in S1 Tables), depending on the province and the purpose of the facility. To prevent breeding, separation of adults in the North West and Limpopo is most common (73% and 63% of facilities respectively). In the Free State, however, lion numbers are more likely to be regulated through live sales to other facilities (78% of respondents), separation of adults (47%) and euthanasia (44%) (Table E in S1 Tables). Poaching of captive lions was experienced by 9% (6 of 64 facilities) of respondents.

Captive-origin lions from South African breeders were acquired by 87% of the respondents to stock their facilities when they opened (Table F in S1 Tables). Wild lions were also introduced when a small number of facilities were established (5%) and included lions from reserves in the provinces of Kwa-Zulu-Natal and the Eastern Cape in South Africa, and Botswana. Wild lions from other African countries (besides Botswana) were reportedly not acquired by any of the 100 respondents to this question. Similar sources were cited for the lions on the properties at the time of this survey (Table F in S1 Tables). No data were requested on how many lions were purchased to establish a facility.

The average size of the properties on which activities relating to lions take place varies per province (mean property size = 2229±2415ha) (Table 2). Areas set aside for hunting are the largest (providing hunting takes place on that property), and are 15 times, 19 times, and 40 times larger on average than areas set aside for keeping, breeding and displaying lions respectively. Hunting areas also occupied 73%±25% of the total property area, followed by breeding (9%), displaying (7%) and keeping (7%) (providing these activities took place on a property) (Table 2).

**Table 2:** Mean (±SD) (a) sizes of 99 properties and, depending on the province and farm purpose, the areas set aside for breeding, keeping, hunting and/or displaying lions, and (b) percentage of the total farm area set aside for the listed activities. Not every activity took place on the respondents’ properties. Results correspond to Question 13. (100 hectares equals 1km^2^ or 0.39mi^2^). This table excludes sample sizes per cell; the complete table with all values are in Table G in S1 Tables.

Activity	Eastern Cape (n = 3)	Free State (n = 38)	Gauteng (n = 5)	Limpopo (n = 13)	North West (n = 39)	Mean ± SD ^e^	Total area of farms
**(a) Mean area, in hectares per farm**							
**Entire property** **(total area)**	*3167±1607*	*1810±2526*	*1296±1587*	*2662±1668*	*2594±2548*	*2229±2415*	*220655*
**Breeding** ^a^	60±49	219±906	63±81	247±748	72±326	165±703	13528
**Keeping/rearing** ^b^	60±49	430±1349	58±58	33±40	78±357	212±896	17767
**Hunting** ^c^	4000±1414	4050±1907	-	1577±644	3287±2700	3163±2405	115090
**Display** ^d^^**,**^ ^f^	-	67±153	56±53	170±410	23±26	78±21	2583
**(b) Mean % of farm area**							
**Breeding**	1.8±1.4%	13.7±20.6%	6.8±7.3%	5.5±16.0%	3.4±9.1%	8.5±16.7% ^g^	
**Keeping/rearing**	1.8±1.4%	13.7±19.0%	6.0±5.4%	1.0±1.2%	3.3±8.6%	7.0±14.0% ^g^	
**Hunting**	60%	53.7±29.3%	-	57.5±32.4%	79.6±20.7%	72.7±24.8%	
**Display** ^h^	-	5.1±5.9%	6.3%±4.2%	18.6±39.4%	4.4±8.6%	7.0±18.5%	

^a^ Total area set aside for breeding lions; some respondents listed the breeding area being the same area as the keeping and/or display area (see footnote f). Includes one facility in the Western Cape.

^b^ Total area set aside for keeping and/or rearing/growing lions.

^c^ Total area set aside for released lions to be hunted in.

^d^ Total area set aside for lions to be displayed or viewed in (whether in cages, compounds or other public viewing types in properties described as sanctuaries, rehabilitation and rescue centres, predator parks, interactive tourism (e.g. petting), guest lodges).

^e^ Includes one facility from the Western Cape.

^f^ ‘Display area’ is mostly not an exclusive delimited area different to the breeding and/or keeping areas. Facilities sometime use breeding/keeping/hunting areas as viewing/display areas. In 15 of 33 responses (45%) the display area was a different delimited space to the breeding and/or keeping area. For 18 (55%) responses, the display area was the same as the breeding and/or keeping and/or hunting area (in 5 cases it was the same as the breeding area only; in 3 cases it was the same as the keeping area only; in 9 cases it was the same as the breeding and keeping areas; in one case it was the same as the hunting area).

^g^ Two facilities did not list total property size, hence *n* values differ from property area above

^h^ Includes properties described as sanctuaries, rehabilitation and rescue centres, predator parks, general tourism, interactive tourism (e.g. petting), guest lodges. See footnote ‘f’ on interpreting the display area.

Provincial hunting and breeding regulations vary per province, on (i) the minimum area of the hunting camps, (ii) the number of lions per camp, and (iii) the minimum waiting time before a lion hunt can commence after the lion has been released into a hunting camp [2]. In the Free State, regulations state that hunting camps must be ≥1000ha, and that ≤10 lions are allowed per 1000ha camp at a time. In the North West, the minimum area does not appear to be stipulated in the regulations, but a personal communication in Williams et al [2] indicated the minimum area was >400ha. In the Eastern Cape, the provincial ordinance stipulates a minimum of 2000ha per adult lion [2]. Regulations for other provinces could not be ascertained [2]. These regulations, and the regulation that Free State farms cannot breed and hunt lions on the same property [2], combined with the limited availability of land suitable for hunting, has resulted in Free State farmers specialising in lion breeding and keeping rather than hunting. Consequently, the facilities in the Free State have set aside relatively larger areas for breeding and keeping than the other provinces (Table 2).

The total area of the 99 facilities that listed the size of their properties and the areas set aside for lion breeding, keeping or hunting amounts to 220,655ha (2207km^2^)—an area equivalent to about (i) 11% of the area of the Kruger National Park (KNP), and (ii) 6.9% of the wild lion range in South Africa (wild lion range in South Africa estimated to be 32,127km^2^ [A. Dickman, pers. comm., February 2019]). The total area set aside for lion hunting alone, in camps by 33 hunting facilities, amounts to 115,090ha (1151 km^2^) (52% of the total area of the 99 facilities, or 6% of KNP) (Table 3). The total area set aside for breeding camps by 82 facilities totalled 13,528ha (135km^2^), or 0.7% of KNP.

**Table 3:** The mean (± SD) size of hunting areas set aside on the properties of 33 respondents, and the number of lions per camp. Results correspond to Question 56.

	Overall	North West	Free State	Limpopo	Eastern Cape ^d^
**No. of respondents**	33	22	4	5	2
**No. of hunting areas** ^a^	1.2 (mode = 1)	1.3 ^b^ (mode = 1)	1	1	1
**Ave. size of first hunting area**	3163±2405ha	3287±2700ha	4050±1907ha	1577±644ha	4000±1414ha
**Size range of facilities’ hunting areas**	1000–12000ha	1255–12000ha	2300–5700ha	1200–2500ha	3000–5000ha
**No. of lions in first hunting area**	1 to 17 lions	1 to 15 lions ^e^	12 to 17 lions ^c^	4 to 5 lions	8 lions
**Ave. no. of lions in first hunting area**	3.4±4.9	1.8±3.7	9.7±8.7	4.3±0.6	-
**Average area per lion**	1079ha lion^-1^	1515ha lion^-1^	405ha lion^-1^	374ha lion^-1^	625ha lion^-1^

^a^ Question 56: (i) how many hunting areas are set aside for hunting; (ii) the size of hunting areas 1, 2 and/or 3; (iii) the number of lions in hunting areas 1,2, and/or 3.

^b^ Only respondents in North West province had a second hunting area (n = 5 had two hunting areas, whereas n = 22 had one hunting area). The average size of the second hunting area was 2140ha±1036ha, but only one property had a lion in the second hunting area (the other four had no lions on them)

^c^ Two facilities had no lions in the first camp at the time of the survey

^d^ Two respondents listed facility and hunting area, but only one listed the number of lions in the area

^e^ Eight facilities listed no lions in the first hunting area.

Congruent with the provincial regulations, the actual areas set aside for hunting varied, with the Free State and North West having the largest areas (not counting the Eastern Cape because of a sample size of one), with the overall average being 3163±2405ha per property (Table 3). The number of lions in the hunting areas is 3.4±4.9, with the average area per free-roaming lion being highest in North West (1515ha per lion) and lowest in Limpopo (374ha per lion) (Table 3). With the exception of the one respondent in the Eastern Cape, the mean lion densities in the hunting areas comply with the regulations.

Finally, provincial legislation also varies on the minimum waiting time before a lion hunt can commence after the lion has been released into a hunting camp [2]: for example, ≥4 days in North West, ≥30 days in Free State, 3 days in Limpopo, and ≥6 months in the Eastern Cape. When asked how long after that mandatory waiting period lions are usually hunted, the responses for the provinces were: (i) North West—up to 60 days, but typically one week; (ii) Free State—nine days to six months; (iii) Limpopo—four days to six months; and (iv) overall—four days to six months, but typically within three weeks. There were no responses for the Eastern Cape.

### Captive lion numbers

A variable response rate to the four questions on annual captive stock numbers from 2015 to 2018 was problematic for data analysis. Accordingly, interpreting the results for the total number of lions in any year and/or for lions of any sex and age is also problematic. Hence, the results are presented as the mean annual number of lions per facility and also indicating the overall proportion of lions of a particular sex/age in a particular year and province, as well as the respondents’ type of association with the hunting industry (Table 4; Tables H–J in S1 Tables). The standard deviations (and thus variance) of the number of lions per facility tended to be large and thus are indicative of the wide range in the number of lions per facility (e.g. in 2018 the number of male lions in facilities ranged from 1 to 300, whereas the number of cubs ranged from 1 to 180; Table I in S1 Tables); accordingly, caution is advised in extrapolating these results to calculate the number of lions on captivity in South Africa since these results show the minimum number of lions in South Africa.

**Table 4:** Total proportion of lions listed by all responding facilities per age and/or sex group. The number of responding facilities differs annually, and 95 respondents provided data for ≥1 year. Results for the proportion and range in the number of lions per facility, facility type, and the proportion per province are in Tables H–J of S1 Tables. Results correspond to Questions 25–28.

31^st^ Jan of the year ^a^	% Adults (≥3 years old)		% Sub-adult (1–3 years old): any sex ^d^	% Cubs (1 year old): any sex ^e^	Total no. (any age or sex)	No. facilities (n = 95)	Mean (±SD) number per facility:		
	Male ^b^	Female ^c^					All (any age/sex)	Adults (any sex)	Sub-adults & cubs (any sex)
**2015**	*29%*	*25%*	*29%*	*17%*	3697	58	64±79	35±48	29±42
**2016**	*29%*	*24%*	*31%*	*17%*	4567	70	67±79	35±46	32±43
**2017**	*30%*	*23%*	*30%*	*17%*	5769	83	70±86	37±54	32±46
**2018** ^f^	*30%*	*25%*	*28%*	*18%*	5389	62	87±114	48±67	39±69
**Mean**	*30%*	*24%*	*29%*	*17%*					

^a^ To 31 January of the respective years.

^b^ Mean number of adult males per facility per year: *2015* = 37; *2016* = 37; *2017* = 42; *2018* = 50.

^c^ Mean number of adult females per facility per year: *2015* = 31; *2016* = 31; *2017* = 31; *2018* = 42.

^d^ Mean number of sub-adults per facility per year: *2015* = 36; *2016* = 40; *2017* = 40; *2018* = 46.

^e^ Mean number of cubs per facility per year: *2015* = 21; *2016* = 22; *2017* = 23; *2018* = 30.

According to the ToPS records for the number of lions of all ages in South African captive breeding and hunting facilities (but *excluding* keeping-only facilities for smaller numbers of lions), there were >8437 lions (of all ages) in 321 facilities by the end of 2016 (>7800 in 297 breeding facilities, and the remainder released into hunting camps; see footnote *a* in Table 1, and Table A in S1 Tables). According to our survey, there were at least 5769 lions of all ages kept at 83 facilities (of all types) by 31 January 2017 (Table 4) (i.e. ±26% of the 321 ToPS registered facilities at the end of 2016), thus accounting for at least 68% of the captive lions in South Africa. We therefore deduce that the survey captured information from most of the biggest breeding operations in the country.

The average number of lions per facility (especially adults, irrespective of facility type) increased annually from January 2015 to January 2018, but especially from 2017 (Table 4). On average, the proportion of lions at 95 facilities was: 30% adult males, 24% adult females, 29% sub-adults and 17% cubs. However, this proportion and range differs somewhat per province and per facility type (i.e. association with the hunting industry) (Tables H–J in S1 Tables); the majority of responding facilities represented in Table 4 breed lions and therefore the overall average number of lions per facility are higher than they would be for non-breeding/keeping facilities (such as hunting farms, tourist facilities and sanctuaries). Bearing in mind the trend reflected in Figure D in S1 Figs, the increase in the average number of lions per facility associated with the hunting sector (also reflected in the ranges displayed in Table I of S1 Tables) suggests that a few larger breeders are accumulating stocks to achieve economies of scale while others down-size or close their operations. Following increased public scrutiny of the ethical aspects of hunting captive-bred lions, especially those that may have been hand-reared or otherwise used for human encounters, both SAPA and PHASA are in the process of establishing ethical and operational standards and accreditation systems for breeding operations and hunting farms. Raising industry standards inevitably raises operators’ production costs and thus typically drives such industry consolidation.

Influenced by the differential provincial regulatory parameters, Free State facilities predominantly breed and/or keep lions, and the number of males has remained approximately one-third of the total number at the facilities (Table J of S1 Tables). The proportion of adult females appears to have increased marginally (but not significantly) in these facilities from 2015. Facilities in the North West, however, are mostly oriented toward hunting, with some lion breeding and keeping also occurring; the proportion of adults (males and females) has steadily decreased there from 2015 (Table J of S1 Tables)—potentially because lions are not being bought and replaced as often, due to the downturn in the hunting market, especially from USA hunting clients. Adult lions accounted for 39% of the total number in Limpopo facilities in 2015 (Table J of S1 Tables), and the proportions of adults increased notably after January 2015 to >60%–probably because facilities are not selling and hunting as many of their adult lions as they were in the past. The average number of lions per facility in Limpopo appears to have doubled from January 2017 to January 2018, but the Limpopo figures are distorted by one 2018 response from a large breeder who did not provide historical data prior to that year and the result is therefore an artefact of sample size biases.

Facilities not specifically declaring any association with the hunting industry had on average 31 lions (of any age and sex) and 45% of these were adults (Table H of S1 Tables). Facilities that are linked to the hunting industry (through selling live lions to hunting farms and/or hunting lions on the property) had three times as many lions (average 89 per facility, any age and sex) and 56% of these were adults (Table J of S1 Tables). Hence, there are higher proportions of sub-adults and cubs at facilities not directly linked to hunting. Between January 2017 and January 2018, the average number of lions in both facility types increased notably compared to previous years (Table H of S1 Tables).

### Estimated value of sales

**Fig 3:**
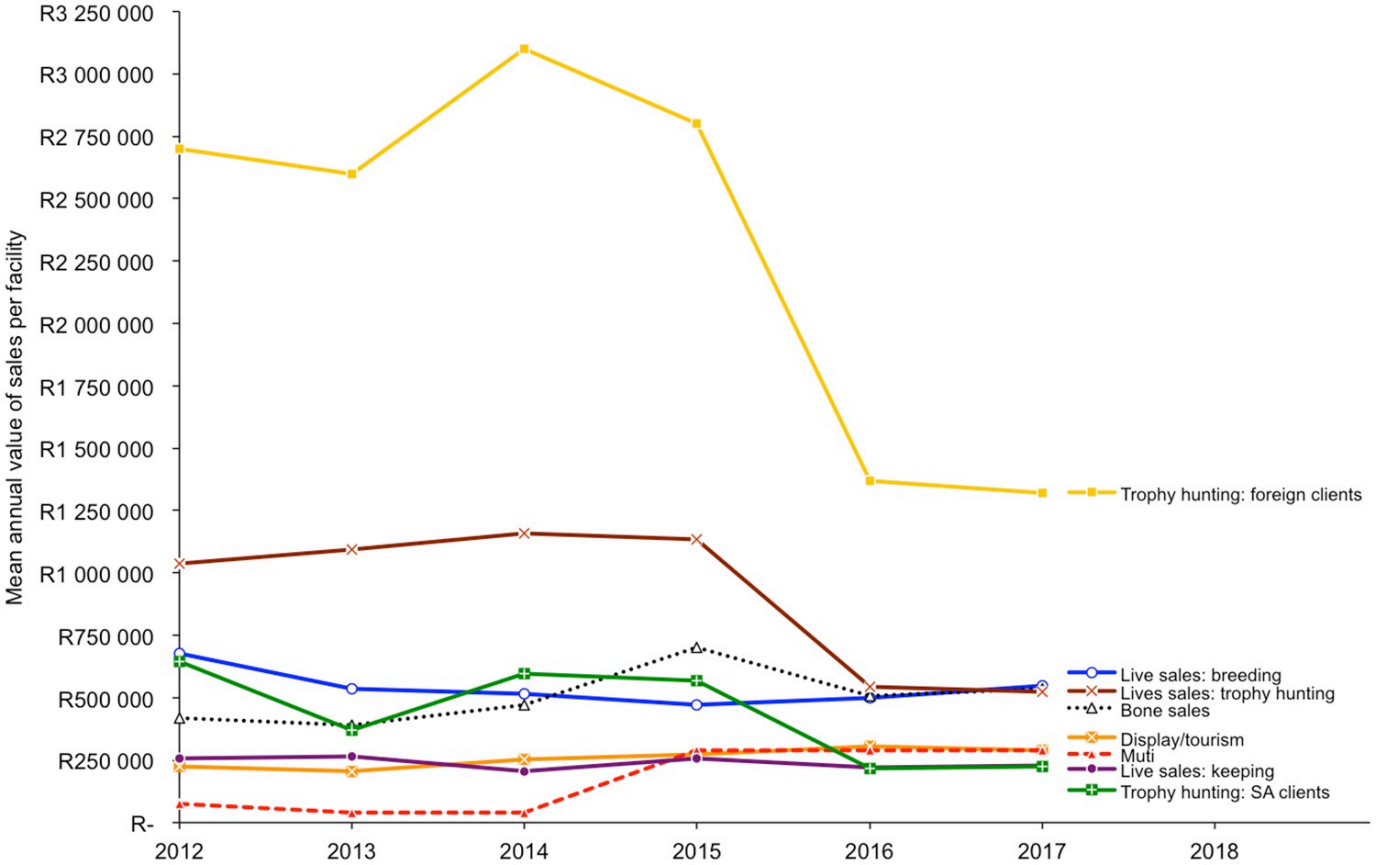
Estimated mean annual value of sales (in ZAR) per facility for income-generating activities from lions. Graph showing values in USD in Figure E in S1 Figs, where mean annual ZAR:USD exchange rates: 2012 (1: 0.122); 2013 (1: 0.104); 2014 (1: 0.092); 2015 (1: 0.078); 2016 (1: 0.068); 2017 (1: 0.075). Results correspond to Questions 15 and 16.

The top income-generating activities for facilities were foreign trophy hunts, live sales for trophy hunting and breeding, and lion bone sales, whereas the activities generating the least annual income were South African recreational hunts, live sales for lion keeping, sales of lion parts for ‘muti’ (traditional medicine in South Africa) and tourism (Fig 3; Table K in S1 Tables). Reported mean values of skeleton sales increased per year from 2012 to 2015 as the number of new facilities entering the market also increased (Table K in S1 Tables); however, the mean value of sales per facility decreased from 2016 (Fig 3). The loss of total revenues from bone sales from 2016 aligns with declines in the total values of live sales for hunting and income from foreign trophy hunts (Fig 3), following the USA lion trophy import restrictions in early 2016.

Changes in the mean value of sales are the first of the results to indicate the joint impact of the USA lion trophy import suspension and the export quota on the bone trade. Breeders that previously benefited from joint trophy hunt and bone markets saw sharply reduced revenues by 2017, and some have not traded at all. We observed that 2016 was an anomalous year that included potential lag effects from carried-over 2015 hunts and sales, as well as strategic behaviour in response to both the USA trophy restrictions and the CITES quota decision (see also [1]).

### Suspension on the import of captive-produced lion trophies

Australia, France and the Netherlands implemented bans on the import of hunted lion trophies in March 2015, November 2015 and May 2016 respectively [19–22] (Fig 1). More significantly, however, in December 2015 the USA listed the African Lion as threatened on their Endangered Species Act, essentially ruling that imports of lion trophies were suspended unless it could be shown that such imports would enhance the conservation status of the species [23]. The suspension, which took effect from January 2016, resulted in an effective ban on USA trophy imports from lions that were captive-born. Since hunting of captive origin lions in South Africa consistently generated the highest revenues of all animals for at least 10 years up to 2015 (in 2016, lions ranked third after buffalo and sable antelope) [2,24], and the USA is South Africa’s largest customer for lion trophies [2,24], it was inevitable that bans and suspensions on the import of lion trophies (especially by the USA) would have negative consequences for the lion hunting and breeding industries. Furthermore, since lion skeletons exported to Southeast Asia up to 2015 were mostly a by-product of the trophy hunting industry, it was expected that these lion trophy import restrictions would alter the nature of the lion bone trade by, for example, increasing the proportion of skeletons derived from euthanized lions supplied by breeders deciding to downscale their operations following the consequent decline in live lion sales to hunting operations.

To gain a better understanding of the consequences of this suspension, including the links between the lion trophy export and bone markets in relation to the captive breeding industry, 25 direct and indirect questions relating to this USA policy (referred to as a ‘ban’ in the survey) and potential import bans by other countries were asked in different sections of the questionnaire. Of these questions, eight were asked of all facilities in Section B (S2 File) (including sanctuaries and tourism venues) (presented in this section), while the remainder were answered only by facilities participating in at least one industry sector (e.g. breeding, hunting, etc) (presented in results to follow).

#### Impact of 2016 USA trophy import suspension

The 2016 suspension reportedly had an impact on the businesses of 82% of the responding facilities; 8% said that suspension had no impact, and 10% answered ‘not applicable’ (synonymous to ‘no impact’) (S3 File). In response to how facilities, if affected by the suspension, were adapting to the impact, 86 responded in the following ways: (i) 63% had scaled down breeding production, (ii) 58% retrenched workers, (iii) 41% sold off lion stocks; (iv) 30% redirected the business to focus on the lion bone trade; (v) 26% euthanized lions; (vi) 10% continued ‘business as usual’, (vii) 5% redirected the business to focus on interactive tourism, and (viii) 17% gave 13 answers for ‘other’. Answers provided for ‘other’ included: shifting focus towards ‘non-export hunts’ (i.e. trophies are not exported following hunts); redirecting the marketing of hunts (e.g. to hunters in other countries); focussing on selling buffalo, sable and roan hunts; and, buying a hunting farm in a different province in 2017 so that the respondent could supply lions from their own breeding facility to their new hunting facility and thus avoid euthanizing lions and/or rely less on the income from live sales to other hunting facilities (S3 File).

When asked how business would adapt if the USA suspension showed no sign of being lifted in the near future, 106 facilities responded as follows: (i) 45% will focus on the lion bone trade, (ii) 35% will convert the business to another form of wildlife breeding, (iii) 21% will close the business, (iv) 17% will euthanize all lion stocks, (v) 16% will continue ‘business as usual’, (vi) 8% will focus on interactive tourism, and (vii) 9% gave 10 answers for ‘other’ [including: entering the hunting market (responses from facilities that currently only breed lions); adapting to the current market] (S3 File).

In terms of other measureable effects, we also asked respondents to what extent certain attributes had increased, decreased or remained the same since the suspension (namely: number of lions, and total areas for breeding, keeping, growing area and/or hunting). The results suggest that some industry consolidation may be taking place, i.e. that some respondents are scaling down or even exiting the industry, but others are maintaining or even expanding their operations to claim a greater proportion of the market (Figure E in S1 Figs). The attribute cited to have changed the most was the total number of lions on the property: for 42% (of n = 99) the number of lions had increased, for 36% the number had decreased, and for 21% the number of lions had stayed the same—a result reflecting the different circumstances of, and strategies adopted by, industry participants. Some facilities with increased numbers of lions had also chosen to accommodate this growth by increasing the areas in which lions were kept, bred and/or hunted (Figure E in S1 Figs).

#### Potential ban on lion trophy imports by the UK and Europe

Cognisant of the bans by Australia, France and the Netherlands, the 2016 USA suspension, and policy reforms and evaluations in several Western countries in the wake of the growing anti-hunting movement in response to the well-publicised hunting of ‘Cecil’ the lion in Zimbabwe [22], it was pertinent to question how the industry might react to further pressure on their businesses IF the UK and other European countries also implemented lion trophy import bans. From the 105 facilities responding to this question, the following adaptations were documented: (i) 35% will focus on the lion bone trade, (ii) 32% will downscale but continue production expecting that the USA ‘ban’ would lift in the near future, (iii) 28% will convert business to another form of wildlife breeding, (iv) 24% will close their businesses, (v) 18% will euthanize all lion stocks, (vi) 15% will continue ‘business as usual’, (vii) 10% will focus on interactive tourism, and (viii) 6% gave six answers to ‘other’ (including: “*Because I have access to food for lion farming and capital has been spent on building cages and to create jobs and* [the facility] *is already established*, *it would make sense to continue farming and give the market what it needs to protect the wild* [lion] *population*. *Because I never did trophy hunting on my premises*, *it is only a result of the industry that there is still a need for bones*. *There will always be hunters who are willing to hunt lions and the need for bones will be there*, *legal or illegal*“) (S3 File).

The responses to the impacts of, and adaptions to, the trade suspension and bans show that, in the wake of the USA policy, a significant proportion of respondents were eager to sell lion bones in the near future, either as part of a strategy to continue commercial lion breeding, or to defray the costs of downscaling and euthanizing animals. This tendency will likely persist if the USA maintains these restrictions and if other trophy export markets (e.g. other European countries) are also constrained or lost and will only be offset by a slightly higher proportion of business closures and complete euthanization of stock.

### Lion bone quota: Impacts and adaptations

To enhance our understanding of the impact of the CITES-mandated lion bone export quota on lion breeders and keepers and their likely responses to it, we asked respondents whether the quota would restrict their businesses in any way—and if so, how would their business be adapted. Of the 107 facilities that responded to the question, 64% (n = 69) said ‘yes’ (the quota would restrict their business in some way), 7% said ‘no’, and 29% said they were not currently in the lion bone business. Of the 69 facilities that said ‘yes’, they indicated that they would adapt their businesses in the following ways: (i) 52% will search for alternative markets for the bones, (ii) 52% will continue selling bones, but downscale, (iii) 19% will close the business, (iv) 14% will continue ‘business as usual’, (v) 9% will stop selling bones, and (vi) 4% said ‘other’.

The four comments specified for ‘other’ are: (a) “*Markets will be destroyed”*; (b) “*Lion bones are only an option if lions are dead*, *or if they are injured during a fight and have to be put down*. *Two or three in five years”*; (c) “*By reducing hunting or lion bones* [from captive animals] *there will be no reason or purpose for lions and they will die as a result of wild populations being poached for the bone market*. *Poachers are ‘lifted’ above the law and smuggling will be like drugs that cannot be stopped*. *The scarcer the bones become*, *the more sought after* [they are] *and the prices will rise as the demand will continue to grow”*; and (d) “*The quota is not practical and currently not functional*, *how will the DEA determine who will get how many exports*?*”*.

In answering these two questions, the respondents implied that a restrictive quota would incentivize people in the lion industry to find alternative channels for bone sales. These comments align with information we obtained prior to the survey, i.e. that SAPA had requested a considerably higher quota in early 2017 than 800, and that lion skeleton exporters we interviewed independently had also expressed concerns that some frustrated aspiring sellers might resort to other (potentially illegal) trade channels. All these factors point to a distinct threat of the development of a parallel illegal market.

### Lion euthanasia

It was evident during the survey planning phase that if live lion sales to hunting facilities declined significantly in response to the consequences of the USA lion trophy import suspension, then there was a high probability of lion breeders euthanizing lions to recoup some loss of earnings and allied costs (e.g. feeding lions they could not sell). Hence, seven questions were asked to assess the likelihood of euthanasia increasing at breeding facilities—including the most direct question (Q23): ‘*has the number of lions euthanized at this facility increased in the last two years*’, and elaborate on ‘*when this started and the reasons*’ why (Questions 18–20 were discussed in the previous section; Questions 34, 35 and 48 will be discussed later).

Of the 105 facilities that responded, 29% (n = 30) answered ‘yes’ (lion euthanasia has increased since 2016), 28% answered ‘no’, and the majority (53%) replied that they were not currently in the lion bone business. Of the 30 facilities that said yes, 18 elaborated on why this practice had increased, including the following eight comments: (i) “*Mature males from 2016 who can not be hunted*”; (ii) *“Began in 2016*. *Before the US ban we ONLY sold lion bones from hunted lions—in 2016 we had to eliminate lions because the facilities were overcrowded and we needed the cash flow to look after the lions”*; (iii) “*After the restrictions on the import of lions were introduced*, *we had to act drastically*. *At this stage we are still feeding the lions*, *but we can’t carry on*. *If the hunts don’t ‘open up’*, *we will have to get rid of all lions”*; (iv) “*2016*. *There was no market for the lions and the animals became too much to maintain*”; (v) “*Must get income from lions as hunting and sales have decreased and I also look more at the breeding of better genes and thus I reduce any lions with bad genes*”; and (vi) “*2018 poor sales to USA clients due to prohibition on hunting trophy imports*”. In addition, one breeding facility that does not euthanize its lions commented “*We did not euthanize any lions*, *but buyers of our live lions may have euthanized them after purchase*”. The remainder of the comments are in S4 File.

The responses to this question re-emphasize our point in a previous section (Lion bone quota), namely that additional carcasses from euthanized captive-origin lions provide an additional source of bone supply for which a large proportion of breeders are likely to seek markets. The extent to which the size of this supply matches the loss of supply from reduced trophy hunts is unclear but must be seen in the light of the implied overall shrinkage of the captive breeding industry (and therefore possible lower output rates in future). A further provincial regulatory parameter to be considered here is that lion euthanasia is prohibited in the North West; hence, unsold lions (e.g. for breeding or hunting) have been translocated to a province where euthanasia is permitted.

### Sales and exports of live lions

Most of the 117 facilities had sold live lions (n = 98, 84%), typically for the hunting industry. Forty-five percent of these facilities (selling live lions) were in the Free State, 36% in North West, 13% in Limpopo, and 3% each in the Eastern Cape and Gauteng. Lions sold for breeding were usually 1–3 years old, but rarely older than 5 years; lions sold for hunting were predominantly ≥3 years old. Before the 2016 USA suspension, skeletons entering the lion bone market were principally a by-product of the trophy hunting industry. However, there was evidence when the survey was being designed that a growing proportion of the skeletons exported to Southeast Asia were being derived from euthanized lions. Hence, we also sought to quantify the ages at which live lions not intended for trophy hunting would be ‘sold for bones’ (i.e. would likely be euthanized); these lions were ≥1 years old (and typically 3–5 years) (Figure F in S1 Figs). The age distribution evident here is that lions sold for breeding purposes are younger, and those sold for trophy hunting and bones are older.

**Fig 4:**
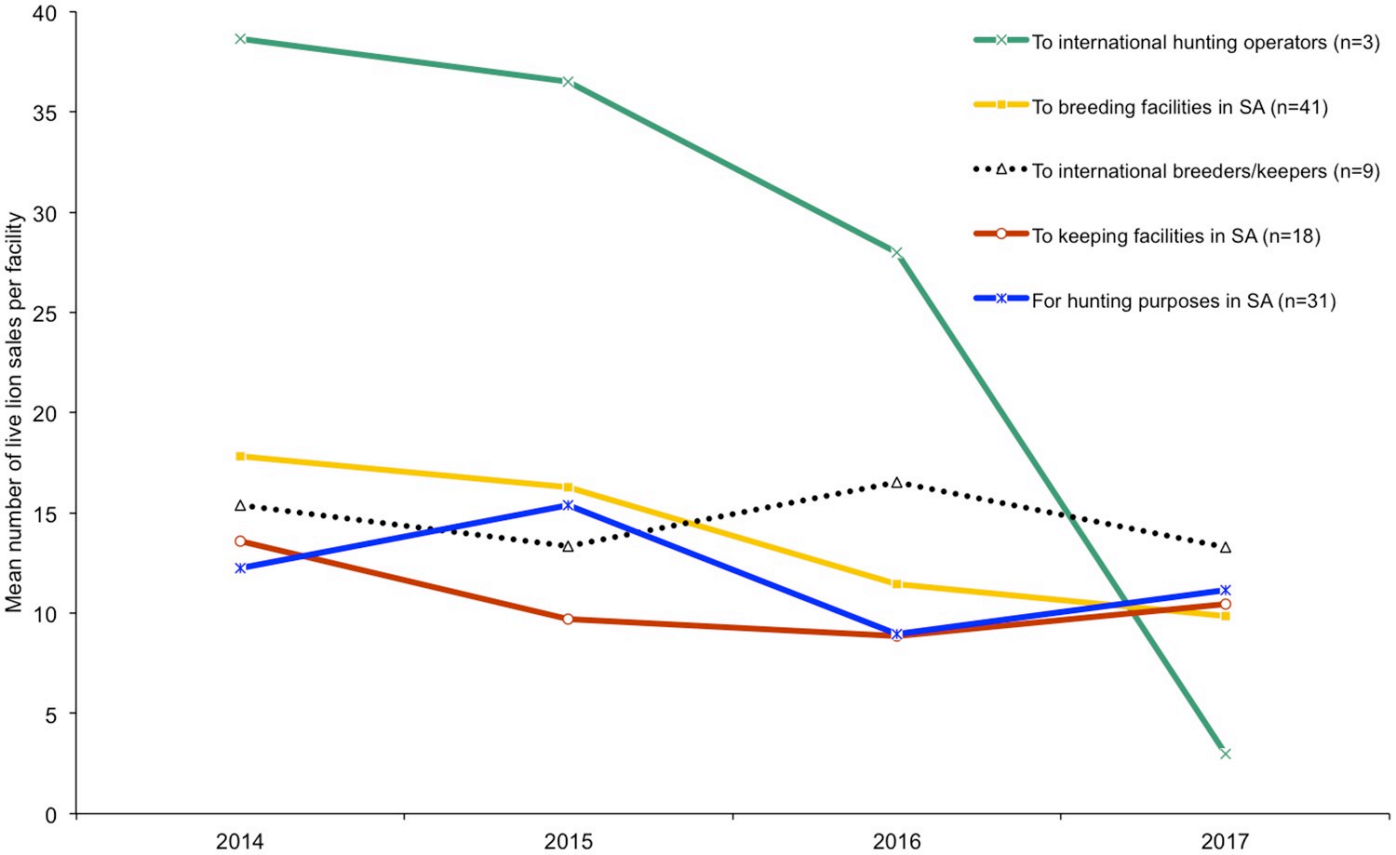
Mean number of live lion sales per responding facility from 2014 to 2017. *Note*: international hunting operators are not based in South Africa and the lions are hunted in the country they are exported to. Results correspond to Question 39.

Interpreting the temporal trends in the numbers of live lions sold by respondents to five facility types is obscured by an inconsistent sample size per annum and per category (Figure G in S1 Figs). In relative terms, however, most lions sold per annum are to breeding facilities in South Africa (average 40% from 2014 to 2017, followed by live lion sales for hunting purposes in South Africa (28%) (Figure H in S1 Figs). While some fluctuations in the proportion of the annual sales going to different facility types were observed over the period (which may be related to sample size variations) (Figure H in S1 Figs), it appears that: (i) the number of live lions sold by South African breeding facilities has declined steadily since 2015; (ii) sales of lions to hunting facilities dipped in 2016 but may have recovered in 2017; (iii) sales to keeping facilities in South Africa show no significant variation; (iv) sales to international breeders/keepers has been relatively stable; and (v) sales to international hunting operators not in South Africa (i.e. translocation of captive bred lions to African countries for hunting purposes) has declined the most, however there were only 1–3 respondents (Figures G & H in S1 Figs). These trends are further confirmed by estimates of the mean number of live lion sales per facility per annum (Fig 4), in which the significant drop in mean annual sales per facility to the international hunting operators is most evident (Fig 4) (but being cognisant of the small sample size, Figure G in S1 Figs).

**Fig 5:**
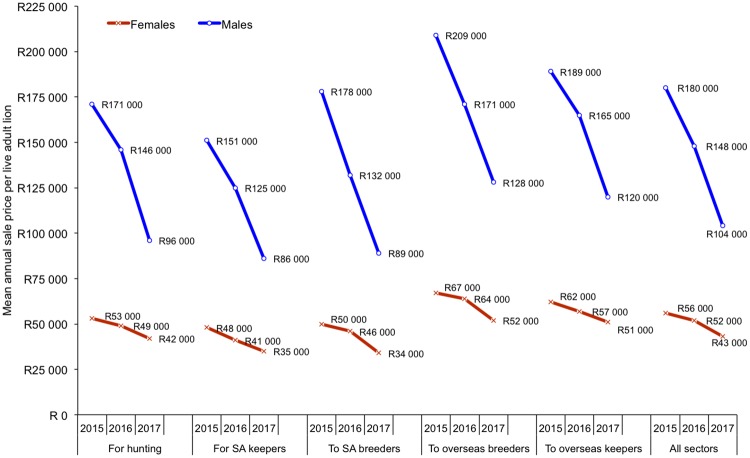
The mean annual sale price of a live adult lion per purpose (rounded up to the nearest R1000). All means, medians, standard deviations and sample sizes are in Tables L & M of S1 Tables, and Figures J & K in S1 Figs. Graph showing values in USD in Figure I in S1 Figs, where mean annual ZAR:USD exchange rates: 2015 (1: 0.078); 2016 (1: 0.068); 2017 (1: 0.075). Results correspond to Questions 41–43.

Across all five sectors, the mean sale price of adult females in 2015 and 2017 was R55,600 (±USD 4337) and R42,300 (±USD 3173) respectively, whereas adult males sold on average for R179,300 (±USD 13,985) and R103,600 (±USD 7770) in 2015 and 2017 respectively (Fig 5; USD values in Figure I in S1 Figs) (USD values are nominal and not inflation adjusted). On average, these mean sale prices decreased by 24% for females and 42% for males, and these decreases ranged between 18% and 50% across the sectors from 2015 to 2017 (calculated from Fig 5). The biggest drops in sale prices were to breeders in South Africa (drop: 32% for females; 50% for males). Sale prices of lions for hunting dropped by 21% for females, and 44% for males. The annual mean, median, standard deviations and sample sizes per sector are provided in Tables L & M in S1 Tables and Figures J & K in S1 Figs.

### Sales and exports of lion products

The structure and nature of the industry plays a role in price formation, and also influences the incentives for individuals to participate in the market. These factors therefore also respond to trade restrictions (including quotas and bans) and are fundamental to the understanding of existing and potential market dynamics. Up to 72% (n = 84) of the responding facilities had on ≥1 occasion sold lion products prior to 2018 (including trophies, body parts, bones, skeletons or other derivatives), and 77 facilities (66%) had either sold, or anticipated they would sell, lion bones into the Southeast Asian market (this information was derived from inspecting answers to all survey questions). No facilities from the Gauteng province responded to questions in this Section (F) of the survey.

In terms of industry structure, intermediary lion bone traders specialise in buying skeletons from various suppliers (viz. farms with lion carcasses) and selling/exporting them to overseas buyers in Southeast Asia. There is evidence that these intermediary traders have the most market power, but some facilities also appear to have direct links to Asian markets. Of the 45 facilities that confirmed they had used an intermediary trader (South African and/or foreign) to export bones/skeletons, nine had at some stage sold skeletons directly via non-South African traders in Southeast Asia. This is relevant insofar that it gives some indication of the potential for parallel illegal markets to evolve and the potential to ‘launder’ illegal products. In this context, ‘laundering’ refers to the fraudulent relabelling of illegally harvested products—from wild lions or other felids—to legal products, and subsequent clandestine introduction into legal supply chains.

#### Lion body part sales

**Fig 6:**
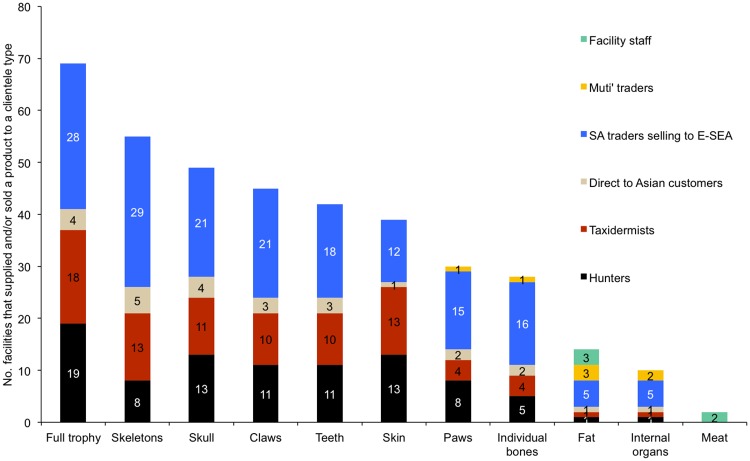
Number of facilities aware of having sold/supplied specific lion products to different clientele. Total respondents n = 52 facilities, where multiple categories and products could be selected for. A ‘full trophy’ includes skeleton, skull, skin, teeth and claws. No responses received for the category ‘Customers in other African countries’. (SA = South Africa; E-SEA = East-Southeast Asia). Results correspond to Question 45.

The complexity of the lion products market is illustrated in Fig 6. In particular, it highlights the existence of both local and overseas markets for lion body parts (and the sale of claws and teeth to export traders who would sell them to Asian customers). It also indicates that facilities are selling both directly to foreign markets and via South African intermediaries. It is also notable that none of the surveyed facilities claimed to be selling lion body parts to other African countries. Products like lion fat and meat tend to be given (not sold) to staff working at facilities.

#### Origins of the skeletons prior to January 2016

Lion skeletons from South Africa have typically entered the market as a by-product of the trophy hunting industry [1,2]. However, after the USA suspended imports of captive-origin trophies in January 2016, there were legitimate concerns that facilities with a lion surplus (from animals that could not be sold to foreign hunters) would reduce captive numbers by euthanizing lions [1], thereby providing another substantial short-term source of skeletons into the bone market. To understand the potential effects of the USA import suspension on the origins of skeletons for sale, we first evaluated the percentage of skeletons from three mortality types that were being introduced into the market before January 2016 (viz. hunting, euthanasia, and/or natural mortality; Question 47); comparisons with the origin of skeletons entering under the quota system could therefore be made from 2017.

In hindsight, Question 48 on the origin (i.e. mode of mortality) of lion skeletons was ambiguous and respondents treated it in two different ways, but with each offering different insights into the skeletons entering the market prior to January 2016:

1. As a sum of the three categories (i.e. combined total from a facility will be 100%) (n = 19 facilities of different purposes)
  - *Hunting trophies*: mean of 63%±45% of skeletons supplied to the market were from hunting trophies (≥90% of skeletons at 11 of 19 facilities originated from trophies; six facilities did not supply skeletons from trophies [respondents 14–19]; Figure L in S1 Figs);
  - *Natural mortalities*: mean of 25%±40% of skeletons supplied to the market were from natural mortalities (100% of skeletons from 4 of 19 facilities originated from natural mortalities; Figure L in S1 Figs);
  - *Euthanized lions*: mean of 12%±31% of skeletons supplied to the market were from euthanized animals (100% of skeletons from 2 of 19 facilities originated from euthanasia; Figure L in S1 Figs).
2. Each of the three categories could potentially be a maximum of 100% (n = 21 facilities)
  - *Hunting trophies*: mean of 72%±36% of the skeletons from available hunting trophies from a facility were supplied to the market (6 of 21 facilities sold all of the skeletons obtained from hunting trophies [respondents 33–40], whereas two facilities supplied only 10% of trophy-origin skeletons to the market; a further eight facilities did not supply any skeletons originating from trophies [respondents 14–19]; Figure M in S1 Figs);
  - *Natural mortalities*: mean of 46%±45% of the skeletons from natural mortalities from a facility went to the market (8 of 21 facilities sold all of the skeletons from lions that died naturally, whereas six facilities only sold ≤5% of the skeletons from natural mortalities; Figure M in S1 Figs);
  - *Euthanized lions*: mean of 66%±45% of the skeletons from euthanized animals from a facility were supplied to the market (8 of 21 facilities sold all of the skeletons from euthanized lions, whereas four facilities supplied only 5%-10% of euthanized lion skeletons to the market; Figure M in S1 Figs).
  - *Note*: these answers do not indicate the relative quantity of these skeletons per facility. If for example, a hunting facility had carcasses from 30 hunted lions, then skeletons of 22 (72%, based on the average calculated) of these would be exported, compared to five skeletons (66%) from a facility with 10 euthanized lions. Hunting facilities would have relatively more skeletons available from hunts and fewer from euthanasia, whereas breeding/keeping facilities would have no skeletons from hunts.

Responses based on both interpretations of this question are revealing. They confirm that, prior to the USA lion trophy import suspension, trophy hunts comprised the main source of exported skeletons (63%±45%). However, they also show that, prior to January 2016 (i) not all skeletons from trophy hunted animals at a facility were exported (28% were not, deduced from 2a above), and that (ii) respondents were likely to export skeletons from available euthanized animals (66%±45% of those skeletons). Facilities were also less likely to sell skeletons from naturally deceased animals; this is most likely due to economies of scale (lions are likely euthanized in batches, as opposed to isolated natural mortalities). By contrast, the sources of skeletons exported as part of the 2017 quota of 800 show that 73% of skeletons originated from euthanized lions, 26% from hunted lions, 1% from damage causing animals, and 0.4% from natural mortalities [18].

#### Skeleton supply to the export market

**Fig 7:**
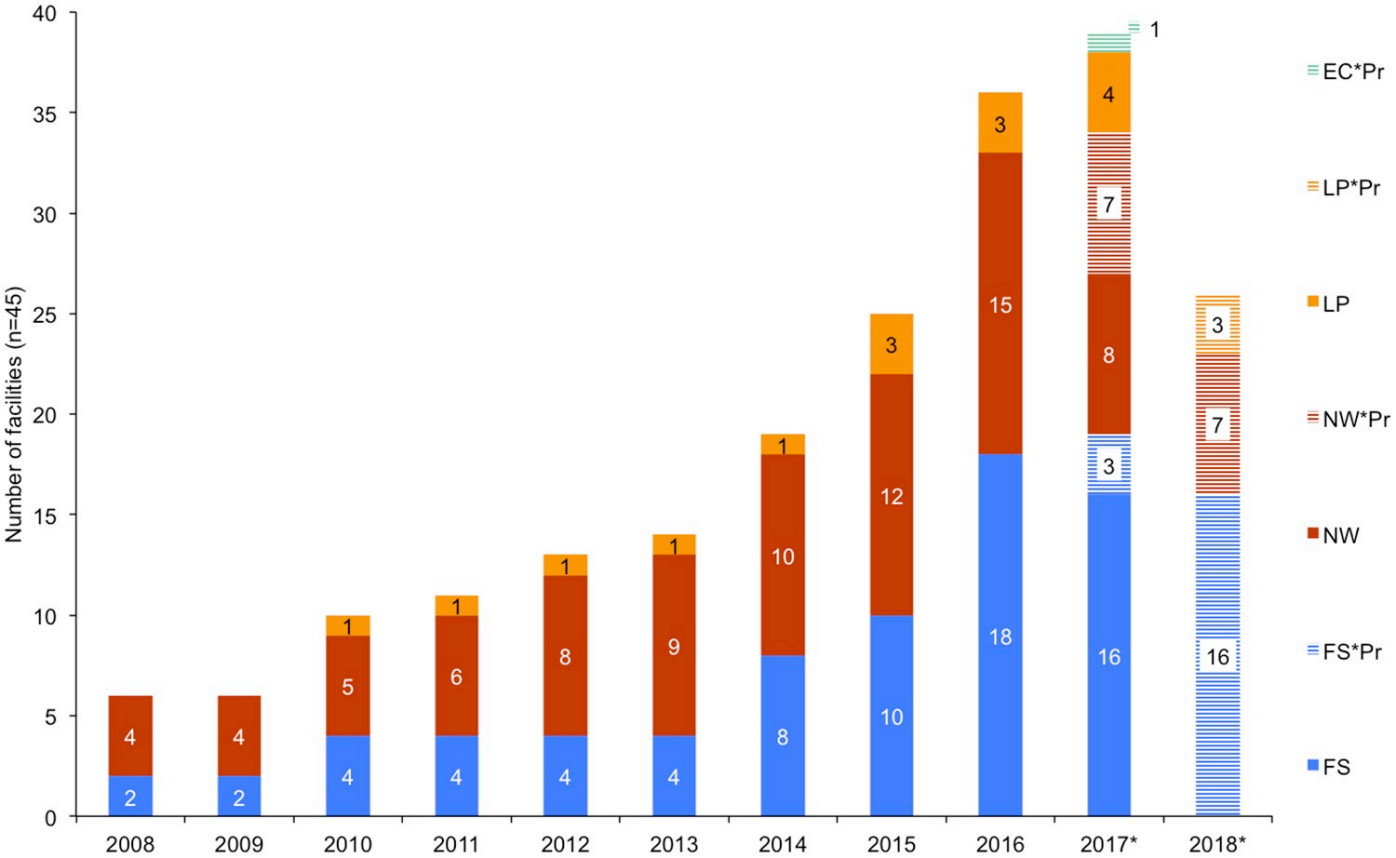
Annual provincial growth in the number of responding facilities saying that lion skeletons from their facility were exported to E-SEA at least once from 2008–2017 (that they were aware of). Legend: FS = Free State, NW = North West, LP = Limpopo, EC = Eastern Cape; the suffix *Pr refers to facilities predicting they would sell bones in either 2017/2018 (depending on the year they took the survey). Results correspond to Question 49.

**Fig 8:**
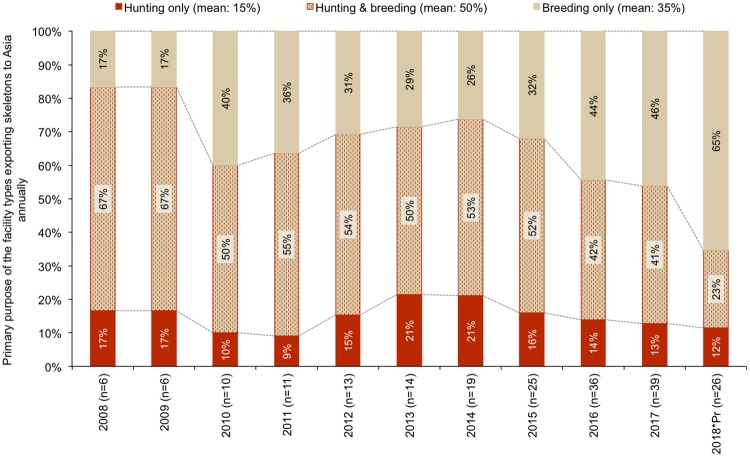
Primary purposes of the facilities exporting skeletons to Asia annually, based on hunting and breeding (sample size, n, after the year). Some facility purposes were documented as dual purpose (hunting and breeding), or a farmer owned a facility with a different purpose in a different province. Results correspond to Question 49.

Participation in the lion skeleton export market has grown since 2008, particularly in the Free State (Fig 7) and by facilities that mainly breed lions (Fig 8, Figure N in S1 Figs). From 2008–2015, the North West Province had more respondents indicating that skeletons originating from their facilities had been exported to Asia (Fig 7); however, from 2016 the Free State had relatively more facilities exporting skeletons—which also coincides with the 2016 dominance of breeders in the industry (>50% of facilities selling skeletons were breeders; Figure N in S1 Figs). The results illustrate the growing significance of the skeleton market as an alternative source of income to breeding facilities, and a decline in the relative number of hunting facilities that have exported lion skeletons, particularly from 2016 (Fig 8, Figure N in S1 Figs). Breeding facilities would sell skeletons derived from euthanized and/or naturally deceased lions to South African bone traders/intermediaries; hence, one can simultaneously infer from the Figures (Fig 8, Figure N in S1 Figs) what proportion of the skeletons exported to Asia are likely to have originated from the breeding industry and from lions that were *not* hunted.

#### Market prices for skeletons

**Fig 9:**
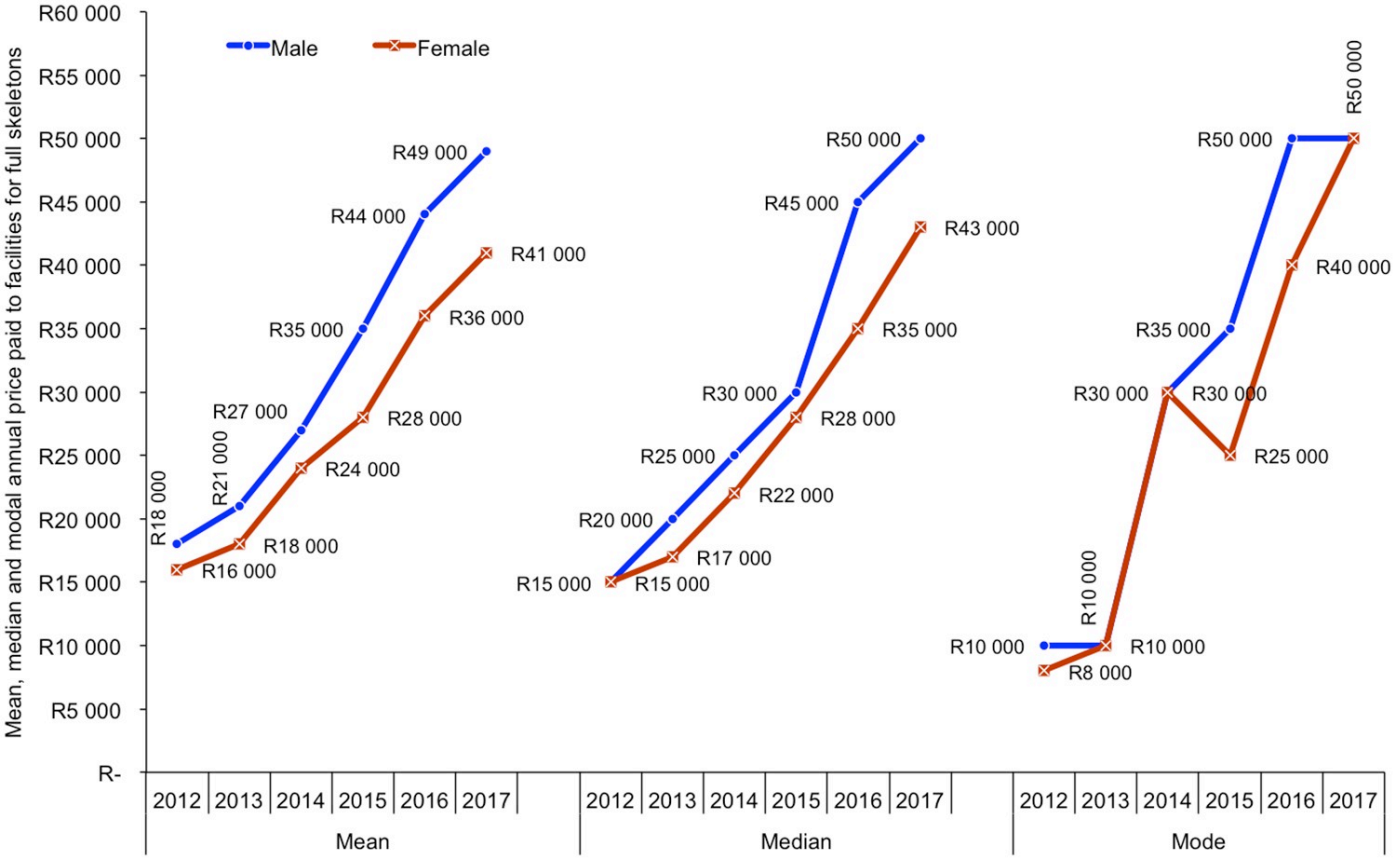
Comparative annual mean, median and modal prices of male and female lion skeletons (2012–2017) sold by responding facilities (n = 35) (mean annual prices, and sample sizes, for male and female lions in Figures Q–T in S1 Figs). Graph showing values in USD in Figure O in S1 Figs, where mean ZAR:USD exchange rates: 2012 (1: 0.122); 2013 (1: 0.104); 2014 (1: 0.092); 2015 (1: 0.078); 2016 (1: 0.068); 2017 (1: 0.075). Results correspond to Questions 50–51.

Market prices provide a valuable indication of market trends—i.e. the extent to which supply is able to meet demand. Rapidly rising prices indicate a widening gap between demand and supply. Price drops suggest that demand for skeletons is declining relative to supply. Collecting accurate price data is challenging, as market participants are often incentivised to report incorrectly (for various reasons). To obtain some indicative price data time series for skeletons we asked facilities to list the prices at which they sold male and female lion skeletons (although these prices are typically set by the buyers and not the sellers) (Fig 9, Figures O–T in S1 Figs).

Prices (mean, median and mode for both sexes) have been consistently rising over the last five years, at a rate that is significantly higher than the official inflation rates in both the producer and consumer countries. The average increase for the mean skeleton price is 22.4% per annum over the period 2012–2017, in contrast with average rates of consumer price inflation of 5.6% in South Africa, 4.4% in Vietnam, 3.1% in Laos, and 1.2% in Thailand over the same period [25] (Fig 9, Figure P in S1 Figs). This is indicative of strong and steady growth in consumer demand. The 2015–2017 prices for males show wider variance than for females (Figures P–T in S1 Figs), with the skeletons supplied from the Free State fetching substantially higher prices than those from North West Province. A closer analysis reveals that this trend of a price differential began in 2014 but increased markedly from 2016 (Figures O–T in S1 Figs). We are aware that skeletons from North West Province are mostly sourced from hunted animals and, as such, typically exclude the skulls and may otherwise be somewhat damaged. In contrast, skeletons from the Free State tend to be intact and include skulls. The data suggest that skeletons with skulls now attract a significant price premium, especially for males: in 2017 this amounted to an average premium of 65% (see Figure S in S1 Figs). This difference between prices of hunted and non-hunted male skeletons, as well as the reported varying quality (due to damage) of hunted male skeletons, provides a plausible explanation for the wider variance in male skeleton prices than those of females.

#### Skeleton export numbers

Twenty-four facilities reported selling skeletons for export to Asia at least once from 2012 to 2016 (Table N in S1 Tables, respondents F19–F42), of which five facilities had sold ≥224 skeletons in total in that period and 10 had sold ≤18 skeletons. Furthermore, seven additional facilities sold skeletons for the first time in 2017 (Table N in S1 Tables, respondents F12–F18), only one of which was a hunting-only facility. If one compares the total number of skeletons exported in a year by these facilities 2012 to 2016 with the *actual* number of skeletons exported (i.e. quantities less than the CITES permits issued) (see Fig 1), then responding facilities allegedly supplied 51%, 71% and 64% of the skeletons exported in 2014, 2015 and 2017 respectively, but only 25% of the skeletons in 2016 (bottom of Table N in S1 Tables). Hence, the facilities that participated in this survey are likely to be adequately representative of the captive lion industry. That only 25% of the 2016 exports are allegedly represented in this survey, is probably indicative of the number of new role-players (primarily breeding facilities) entering the lion bone industry from 2016 and also suggests that the 2016 export figures may include some larger breeders that euthanized all their stocks and exited the industry.

**Fig 10:**
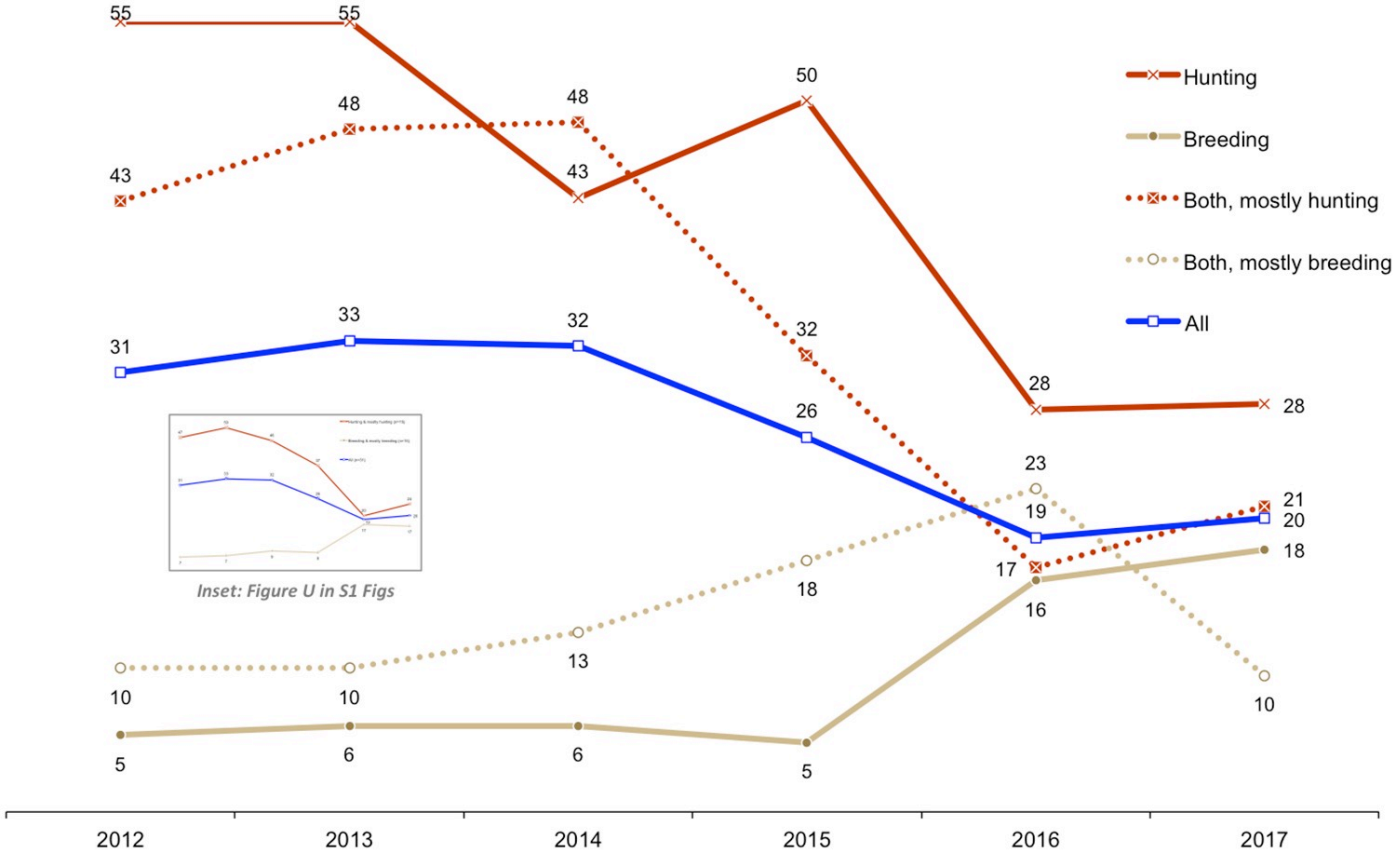
Comparative annual mean number of skeletons sold by responding facilities according to the primary purpose of the facilities (hunting and/or breeding). Dual-purpose facilities (‘Both’ hunting and breeding) were delimited based on answers to Question 11 and other survey questions. (Annual sample sizes and standard deviations are in Table N in S1 Tables). The inset Figure U in S1 Figs simplifies these results to three series. Results correspond to Question 52.

The average annual number of skeletons supplied per facility dropped significantly from 32 to 19 in 2014 and 2016 respectively (Fig 10, Figure U in S1 Figs; Table N in S1 Tables). In particular, there was a notable drop in the average number of skeletons supplied by hunting facilities (and/or facilities whose primary purpose is hunting even though lion breeding also occurs) and a near-mirrored increase in the average number supplied by breeding facilities (and/or facilities whose primary purpose is breeding even though lion hunting also occurs) (Fig 10, Figure U in S1 Figs). The 2016 mirrored convergence in the numbers exported by hunting and breeding facilities is almost certainly a result of the 2016 USA import suspension. The suspension resulted in: (i) a downturn in the South African captive hunting industry, and (ii) thus a decline in the number of hunting-origin skeletons, which was (iii) coupled with a corresponding increase in the supply of skeletons from breeding facilities some of whom, unable to sell lions to hunting farms, chose to euthanize lions to recoup some of the costs associated with keeping lions that they were unable to sell to hunters.

The number of skeletons that facilities said they could supply without restriction varied. In total, 42 facilities indicated they could supply >1892 skeletons, an average of 49±61 per facility (Table N in S1 Tables), and more than double the skeleton quota of 800 set by the DEA in 2017. Twenty of the 23 facilities that sold bones in 2016 could, if there were no restrictions, currently supply more bones than supplied in 2016, and two facilities said they could supply the same quantities (Table N in S1 Tables). On average, facilities alleged they could supply 247%±378% more bones in 2018 compared with 2016 (although listed 2016 export quantities were notably down from previous years) (deduced from Table N in S1 Tables). When compared with 2015 exports however, the number that could be supplied without restrictions was 202%±320% higher. The exports from 2012 to 2015 tended to be weighted towards facilities supplying skeletons as a by-product of recreational hunts, and outputs in that sector dropped in 2016 and 2017. Since 2016, breeders appear to be euthanizing lions on a larger scale.

### Lion hunting

The lion hunting industry is a source of skeletons for the Asian export market. While 79% (n = 92) of the respondents had/have links to the hunting industry (e.g. breeding, live sales and/or hunting), only 33 (28%) allow hunting on the property—of which the purpose of 11 (9.4%) facilities was for hunting only (excluding keeping), four (3.4%) were for hunting and keeping, and the remaining facilities were either dual purpose (i.e. breeding and hunting took place on the property) or they sold live animals to be hunted. There were 26 respondents to the last Section G of the questionnaire on lion hunting: 13 were hunting-only with keeping facilities, 10 were dual-purpose (hunting and breeding) but primarily hunting facilities, and three were dual-purpose but primarily breeding facilities.

#### Hunting clients

Clients from the USA dominated the lion hunting industry prior to 2016 (Table O in S1 Tables) (see also [2,24]). Following the January 2016 USA import suspension, there was a significant drop in the proportion of USA hunters visiting facilities and hunting lions. Since hunts (local and international) are typically booked as safari ‘packages’ that include other species, the USA clients tended to cancel their packages after January 2016 and there was a concurrent reduction in the hunting of other species on the farms. The only drop in the percentage of clients of a specific nationality visiting hunting facilities after January 2016 was for USA nationals (mean decrease per facility -34±46%). The percentage of clients from all other countries increased, especially from Asia (11±26%), ‘other’ parts of Europe (21±31%) and unspecified ‘other’ (33±38%). While there was a slight growth in the percentage of clients from the United Kingdom, Middle East and Canada, these changes are not significant.

#### Number of lions hunted per facility

**Fig 11:**
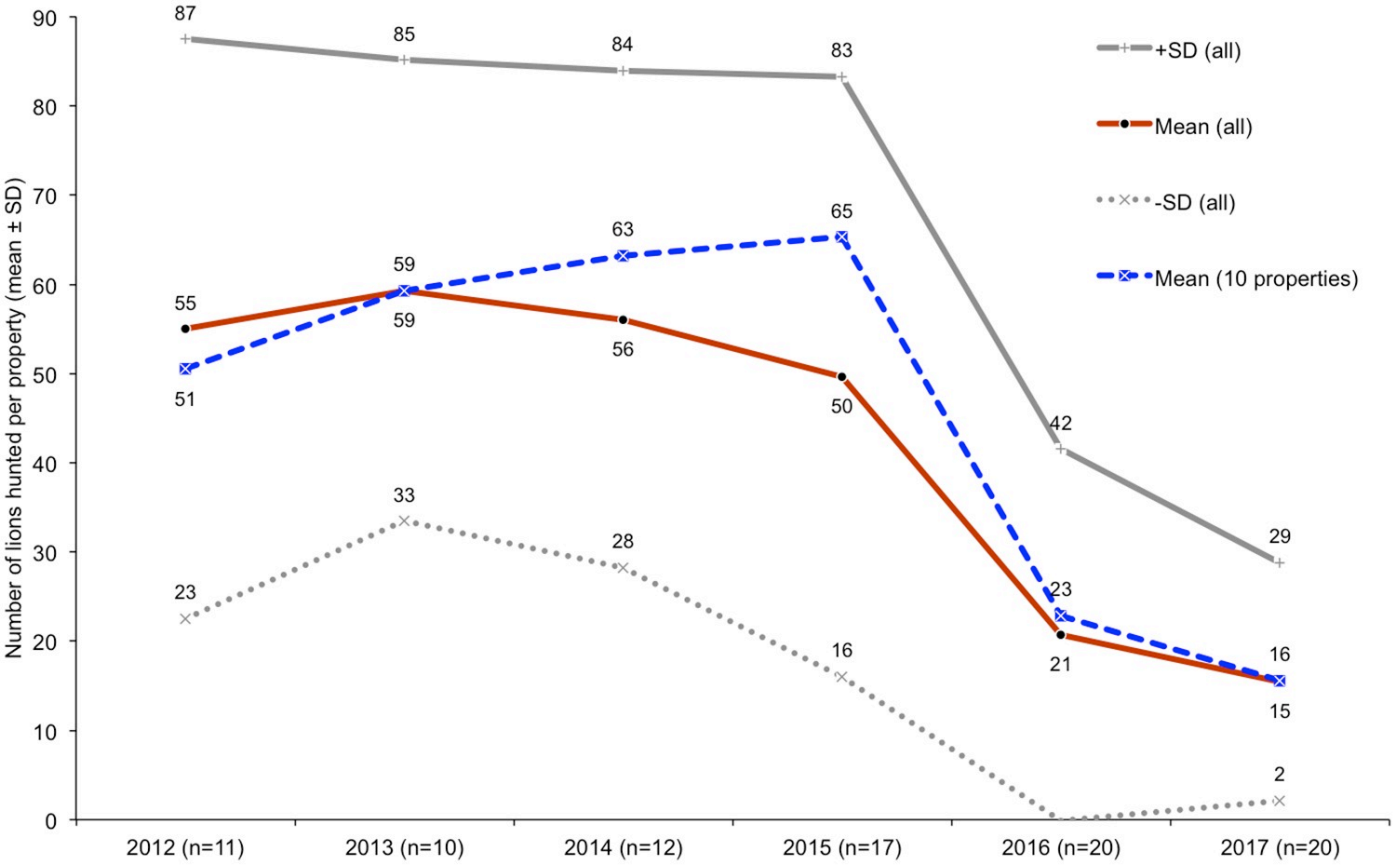
Mean (±SD) number of lions hunted per facility (all facilities, plus the 10 accounting for most of the lion hunts in the period) from 2012 to 2017. Sample sizes in brackets after the year. Results correspond to Question 59.

Further evidence of the 2016 USA import suspension’s impact is apparent in the significant drop in the mean number of lions hunted per facility, and the continuing decline thereafter through to 2017 (Fig 11). The average number dropped from ≥50 up to 2015 to ≤21 from 2016 –a decline of 58% in lions hunted. A total of 3438 lions were hunted on the properties of 22 respondents from 2012 to 2017. Hunting took place every year on 10 of these properties, amounting to 2768 lions (81% of the 3438 lions)–averaging 65 lions per year in 2015 (Fig 11). The start of the decline in income from cancelled lion hunts was experienced by 75% of 24 respondents in the first quarter of 2016, and in the second quarter by 21%. There was a slight lag effect for the dual-purpose facilities (± 2 months delay on average).

#### Loss of earnings, and jobs, at hunting facilities since January 2016

Cancellation of lion hunts following the January 2016 USA import suspension resulted in loss of farm earnings to hunting facilities, and job losses. The total loss of earnings to the 21 hunting facilities that responded to Question 61 amounted to >R115.7 million (USD 8.6 million)–an average annual loss of R5.6 million ± R5.4 million per facility, a median loss of R3 million, and ranging between R350,000 to R20 million per facility (average annual ZAR: exchange rate 1:0.074, based on: 2016 1:0.068, 2017 1:0.075, 2018 1:0.078). Six of these facilities, however, reported losses of R10 million to R20 million (mean = R12.7 million ± R4 million, median = R10.9 million) (S5 File shows two case studies). Three hunting facilities did not report the amount lost, but said it was in the region of 25% to 50% (mean ±40% loss of income per facility). These losses sometimes included income from plains game not hunted as part of the same package once the lion hunts were cancelled (S5 File). Since lions are frequently the ‘draw card’ to many international hunters interested in shooting the ‘Big 5’, it seems some hunters cancelled entire hunting packages involving other income-generating game once lion shooting was disincentivized.

Loss of farm income also resulted in some farms retrenching workers or selling the farm. From the 27 facilities that answered the question, the following retrenchments were reported: (i) average three skilled workers per farm (range 1 to 10); (ii) average seven unskilled workers (range 1 to 20); (iii) average two professional hunters (range 1 to 3). The total number retrenched by these facilities amounted to 243 workers (mean = 9±8 per facility; median and mode = 6).

### Final respondent comments

In Version 2 of the survey we included an open-ended question (Q63) to allow respondents to share any additional thoughts. The full set of responses received is in S6 File, but collectively they represent a reasonably consistent narrative. The comments reveal that respondents believe that the industry plays a positive supporting role for both lion conservation and the South African economy and also acts as a buffer against overexploitation of wild lions. More than one respondent suggested that they were breeding for improved genetics. One was highly critical of the regulatory environment, noting inconsistencies between provinces and alleging deficiencies in the permitting system. However, respondents generally appeared to be in favour of strengthening the industry through appropriate regulation and cautioned against stifling it.

Most respondents view the hunting market as the most important industry component, with the bone trade providing supplementary support. They lamented the impacts of the USA lion trophy import suspension and appear keen to rebuild the hunting market, relying on income from skeleton exports while they attempt to achieve this. Whereas one respondent stated that “*a controlled bone market is better than an uncontrolled illegal market*”, there were varied opinions on the quota: from recommending a set number (1,600–2,000 skeletons) per annum to no limit at all. One respondent suggested that a quota should be applied to hunted animals only, not those that were euthanized. In general, respondents were keen to maintain open markets to support continued breeding and sale of live lions, failing which they might consider euthanizing them.

## Discussion

The survey provided useful baseline information on South Africa’s captive lion industry. The sample limitations of the first round (in 2017) were overcome after the follow-up round (in 2018), although receiving responses in two separate years created some challenges for collation and interpretation of the data. Mindful of these, we attempted to account for any resultant discrepancies in our analyses. Given that this industry is currently subject to significant activist pressure, we had to overcome the reluctance of participants to divulge data and therefore potentially sacrifice some elements of rigour. Furthermore, much of the data and analysis thereof falls within the realm of social science, in which certain trade-offs of this nature are inevitable. Nonetheless, the final sample was sufficiently large and with enough consistency of data and trends for us to feel confident in the integrity of the findings.

The results provide not only a substantial geographic overview (by provincial jurisdiction) of the industry, but the time series data provide insight into the economic impacts of a significant policy intervention (the USA lion trophy import suspension), as well as consequent implications for conservation. The qualitative elements of certain responses provide additional insight into industry participants’ motivations and possible future actions. This information is likely to be of interest to scholars from diverse disciplines, broadly including law, economics, and conservation science; and, more narrowly including industrial organisation theory, economic geography, economic sociology, environmental regulation, trade regulation, and decision theory.

The results indicate that the captive lion industry is in an economically unstable state, having been significantly affected by the 2016 USA lion trophy import suspension and implementation of the skeleton export quota in 2017. The USA suspension has also changed industry perceptions and behaviour in respect of the export market for other lion body parts. Whereas industry participants viewed bone exports to Asia as an optional supplementary by-product market from the years 2008 to 2015, their interest in this market grew immediately following the suspension; this growing interest is also likely driven by increasing market prices for skeletons over the last five years. Furthermore, evidence suggests that 2016 heralded a new era for the trade in body parts once large volumes of intact skeletons from euthanized lions entered the export market. The price data reflect a significant and rising price premium for skeletons with skulls, reaching price levels for females that are now close to their live sales prices. It therefore appears that the USA intervention has inadvertently spawned a new lucrative direct export market for whole skeletons of euthanized lions from breeding farms.

Industry participants are adapting to the current trade restrictions in different ways. Those involved with hunting are seeking new markets and this sector may grow again, albeit at slower rates. However, if there is no substantial change in USA policy, or if there are further EU lion trophy import restrictions, breeders will feel pressured to explore further options to mitigate expected financial losses. Different breeders will continue to respond in various ways, which are not easy to predict. Some are likely to scale down significantly, if not disinvest from lion breeding altogether. At least some of these will euthanize their animals and attempt to recover costs through sale of skeletons. Most breeders appear hopeful or expectant of at least some ongoing access to an export market for lion body parts.

There are now three apparent types of potential lion skeleton exporters: (i) the hunting industry participants who seek to continue the traditional by-product trade, (ii) down-scaling breeders who are euthanizing their animals and attempting to recoup financial losses, and (iii) a new potential category of commercial breeders who might deliberately continue to act as bespoke intact skeleton suppliers. The extent to which the third category is capable of persisting as a viable stand-alone business sector remains to be seen; nevertheless, all three sources must compete for allocations of any future skeleton export quotas. The South African government must evaluate its quota-setting policies against this backdrop of both increasing economic pressures for all industry participants to become sellers and evident increasing demand from Asian buyers (reflected by rising prices, especially for skulls).

The fact that a large proportion of respondents have stated that they will seek ‘other markets’ for lion bones and other body parts signals the potential for a parallel illegal market to develop if quotas are viewed by industry participants as excessively restrictive. South Africa experienced a similar situation with rhino horn trade, whereby increased restrictions were contested by market participants, sparking a significant wave of illegal activity [26]. Should any South African captive lion industry participants develop closer links with organized criminal enterprises, the effects could be irreversible and result in greater and more widespread threats of focused commercial-scale poaching of wild felids. Well-informed existing legal exporters of lion skeletons (who are not owners of lion breeding or hunting facilities) share these concerns and the survey responses (indicating the belief that the captive lion industry acts as a buffer to protect wild lion populations from overexploitation) support this further.

Aside from considering a possible buffer effect of legal body part exports, questions remain on the conservation role of captive lion breeding for hunting. The sizes of the lion hunting areas suggest there may be indirect benefits to biodiversity conservation, i.e. through provision of economic support to maintain the natural integrity of these privately-owned ecosystems, which might otherwise be lost through conversion to other forms of land use such as agricultural monocropping. We recommend further research on this aspect, especially given the evident impact of the USA lion trophy import suspension. This impact is reflected by falling sales of lion hunts and numbers of live lions sold, consequent falling income levels and market prices of live lions, widespread operational down-scaling, losses in human employment, and loss of hunting clientele (for other species in addition to lions). These impacts could ultimately lead to land conversion and consequent biodiversity loss. Possible impacts on hunting of wild lions elsewhere remains a subject of some conjecture and also warrants further research.

Given an objective of informing future trade policy decisions, the results indicate areas that warrant ongoing monitoring and investigation. These include: (i) the total number of captive lions, (ii) trends in the market prices paid for live animals and skeletons (including the premium paid for skulls), (iii) the prevalence of lion poisonings and poaching on private property, (iv) how facilities continue to adapt to trade bans/restrictions and/or a quota, and (v) the changes/losses in earning across all sectors of the captive lion industry. Such information can guide any decisions made under an adaptive management approach aimed at minimising the risk of adversely affecting wild lion populations. In this regard, one of the most useful aspects to understand would be the price elasticity of demand for lion body parts. Monitoring market price responses to changes in quantity supplied would provide useful indicative evidence of this, suggesting another appropriate use for carefully considered quota adjustments. However, policy-makers should take care to avoid price shocks—i.e. sudden upward spikes in market prices that would boost incentives for poaching and illegal trading activity, with potential adverse consequences for wild lions.

Critics of the captive lion breeding industry raise animal welfare and ethical issues in addition to specific conservation concerns, whereas proponents of the industry argue that it provides both broader conservation and economic benefits to society, even if the former are somewhat indirect and not immediately obvious. These contrasting views highlight some inherent trade-offs in addressing multiple, potentially conflicting social policy objectives in the context of a complex adaptive system with global reach. In this regard, policy toward the captive lion industry may be viewed as a ‘wicked problem’ [27], for which there is no universally optimal solution, and one for which stakeholders may even disagree over the definition of the problem *“if they are personally invested in pursuing a particular solution*” [28]. This represents a serious challenge to those seeking to determine appropriate management actions by way of collaboration between all stakeholders. Current broader research on this topic considers techniques to gain a deeper shared understanding of the areas of risk and uncertainty and how to address them, given the substantial differences in values evident among stakeholders. Such techniques include participatory scenario planning and ethical argument analysis [29], both of which may provide additional guidance on these complex policy decisions. We anticipate that the results we have presented here will illuminate and inform ongoing deliberations, but they are certainly not the final word on them.

## Supporting information

S1 Figs PDF of 21 supplementary figures, from Figures A to U. (PDF)

S1 Tables PDF of 18 supplementary tables, from Tables A to O. (PDF)

S1 File National captive lion survey, Version 2 (English) revised for 2018. (PDF)

S2 File Survey questionnaire overview. Sections, questions numbers, questions, and the number of responses per question.
(PDF)

S3 File Responses to the ban on imports of captive-produced trophies. Results correspond to Questions 17–20.
(PDF)

S4 File Lion euthanasia. Responses to Question 23.
(PDF)

S5 File Two case studies describing specific loss of earnings and jobs in the North West and Free State provinces. Includes responses to Questions 61 and 62.
(PDF)

S6 File Final respondent comments. Responses to Question 63.
(PDF)
