# Supplementary material for: Born captive: A survey of the lion breeding, keeping and hunting industries in South Africa
Source: PLoS One. 2019 May 28;14(5):e0217409. doi: 10.1371/journal.pone.0217409 (PMC6538166; doi:10.1371/journal.pone.0217409)
Supplement: S1 Figs — (PDF) [file pone.0217409.s001.pdf]

S1 FIGURES

Supplementary Figures A to U

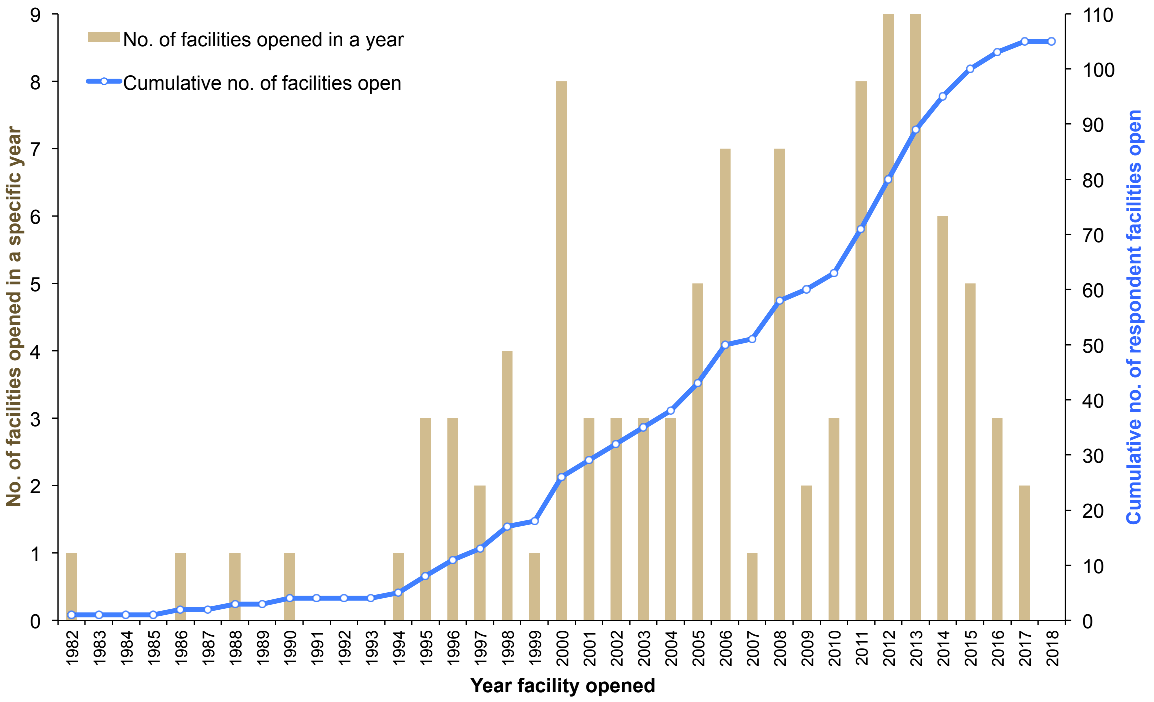

Figure A. Number of responding facilities opened by year (beige histogram), and cumulative number opened (blue line), between 1982 and 2017. N=105 respondents. Excludes 12 facilities that skipped the question. Results correspond to Question 5.

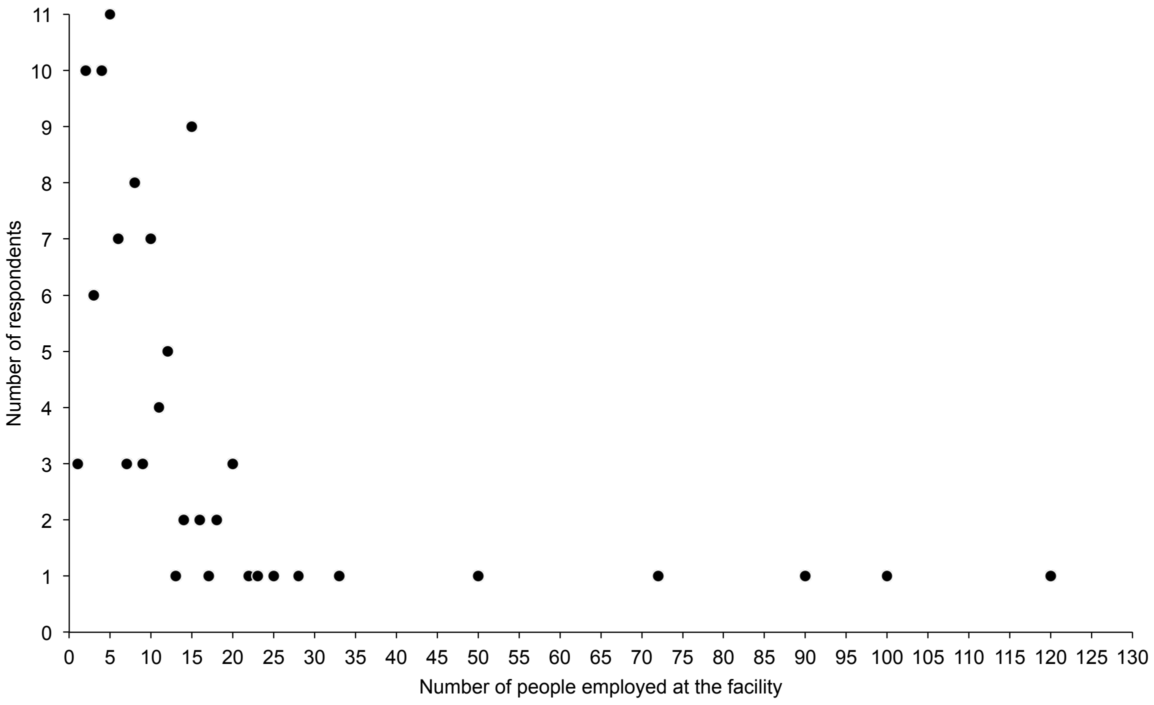

Figure B. Frequencies of the number of people reported to be employed at the responding facilities. N=107 respondents. Results correspond to Question 8.

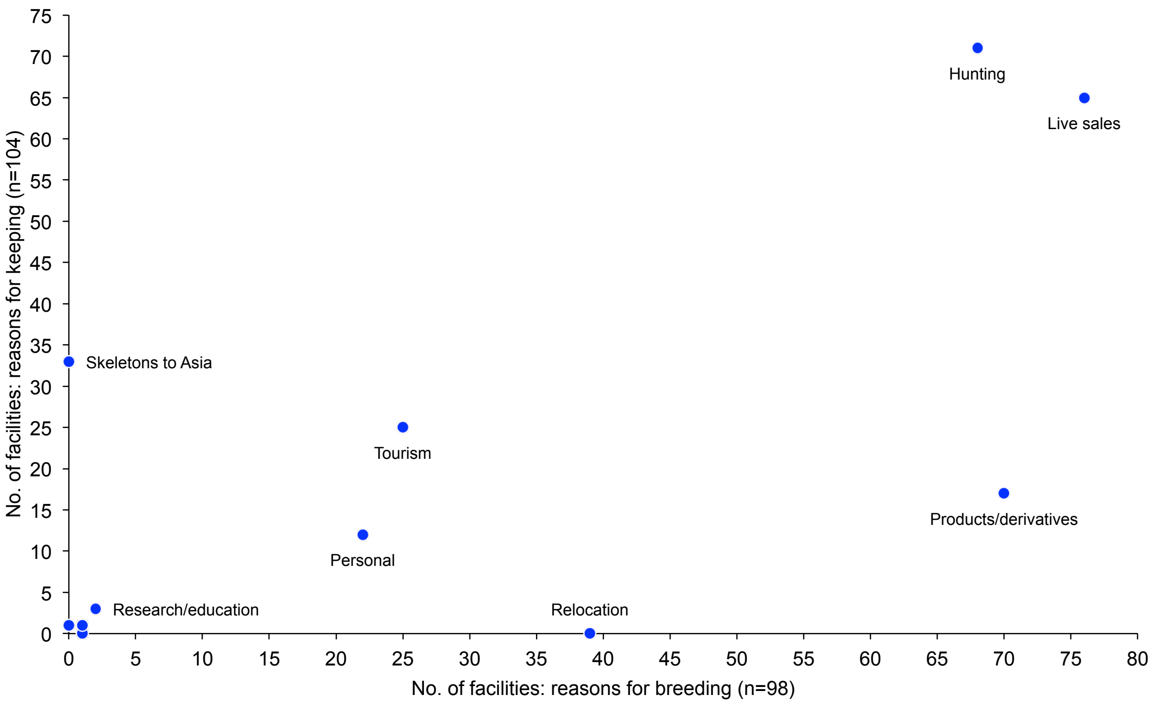

Figure C. Frequencies of the number of facilities listing specific reasons why lions are bred (x-axis) and/or kept (y-axis). Reasons with ≤1 respondent are not labelled, and are listed in Table C in S1 Tables. Results correspond to Questions 9 & 10.

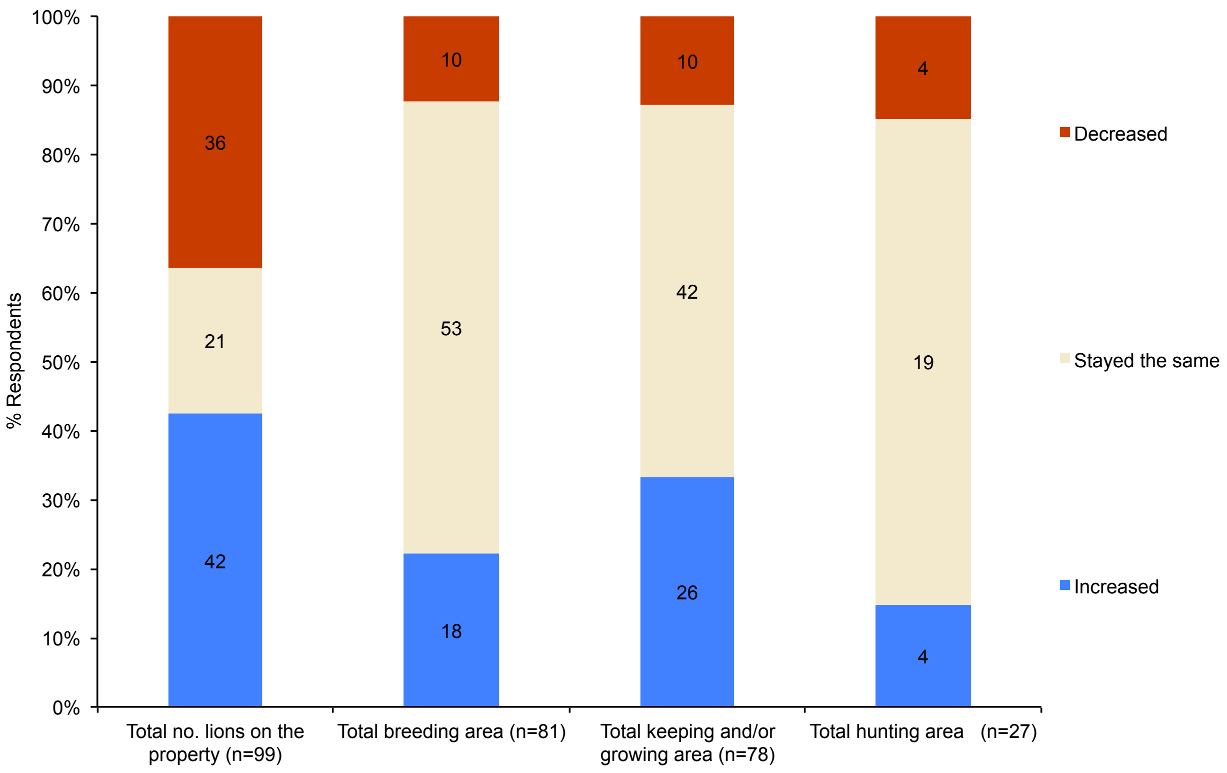

Figure D. Percentage of respondents indicating whether there had been any changes to the number of lions on the property and the areas for breeding, keeping or hunting lions since January 2016 (n=105; no. respondents in the histogram). Results correspond to Question 14.

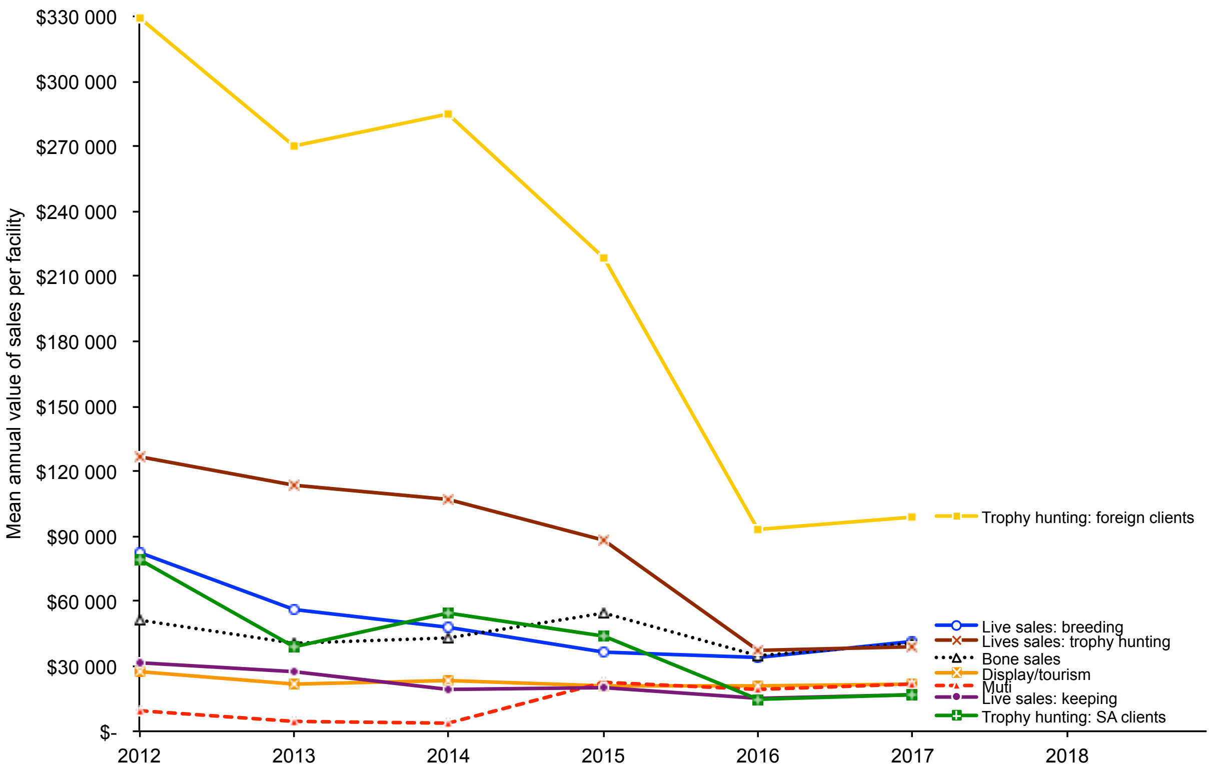

Figure E. Estimated mean annual value of sales (in USD) per facility for income-generating activities from lions. These are nominal figures (i.e. not inflation adjusted). Mean annual ZAR:USD exchange rates: 2012 (1: 0.122); 2013 (1: 0.104); 2014 (1: 0.092); 2015 (1: 0.078); 2016 (1: 0.068); 2017 (1: 0.075). Results correspond to Fig 3, and Questions 15 and 16.

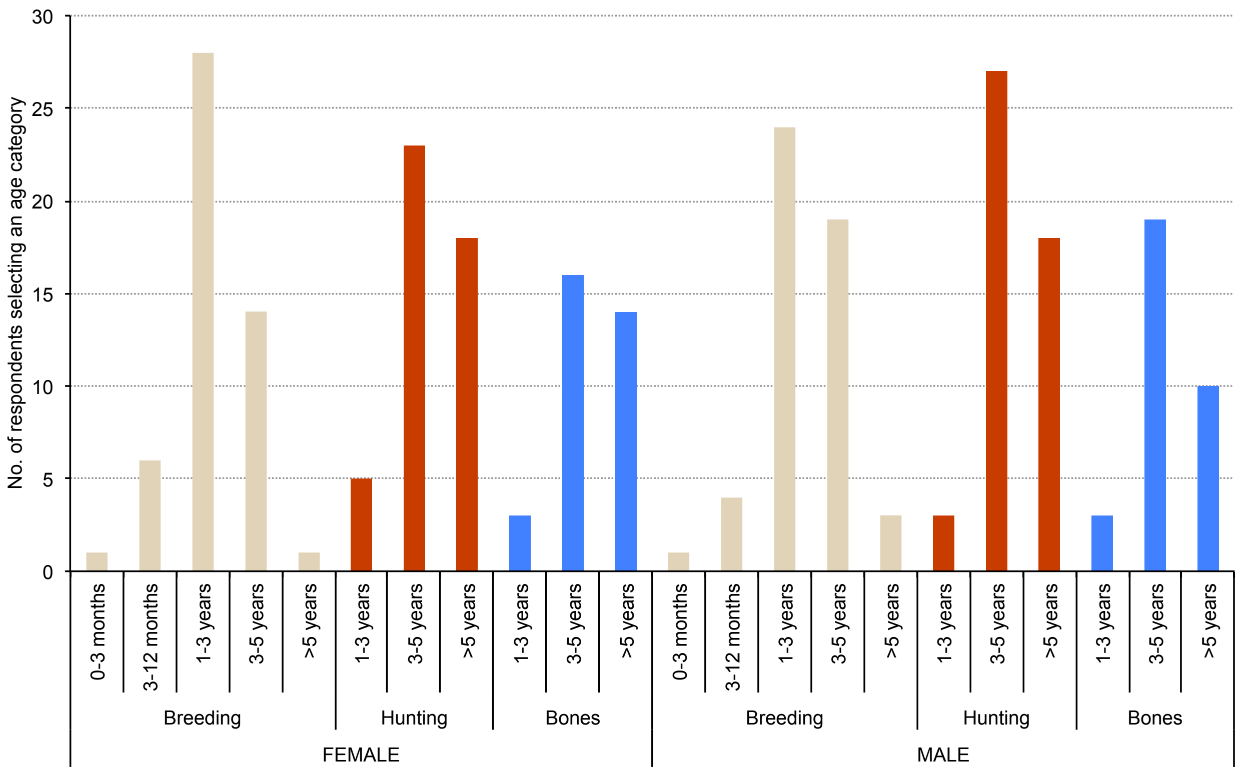

Figure F. The number of responses for the average age lions and lionesses are sold for specific purposes (n=60 overall). **Breeding** purposes in **beige** (n=53), **hunting** purposes in **red** (n=48), and inclusion into the **bone** trade in **blue** (n=34). No question was asked on the ages of lions sold to keeping facilities. Results correspond to Question 40.

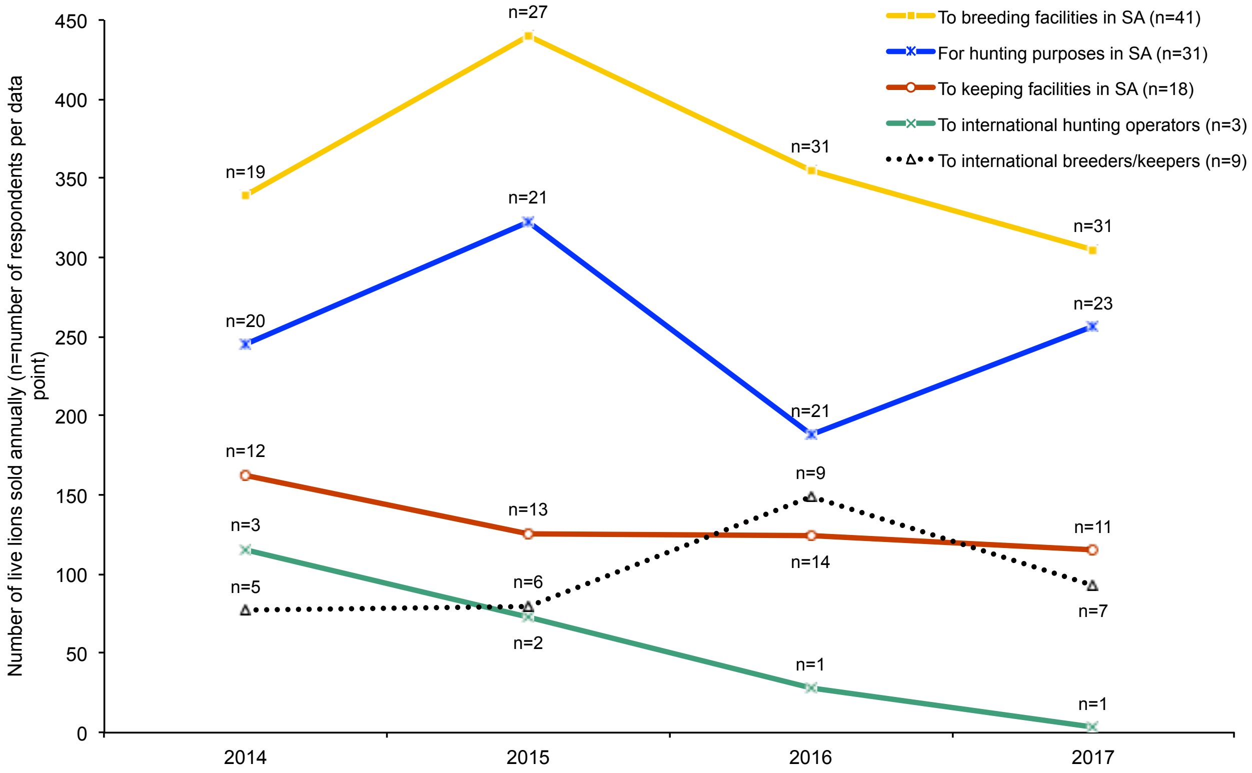

Figure G. Estimated number of live lions sold annually to five facility types from 2014 to 2017, with variable sample sizes (n). Since sample size is variable for the annual estimates, the data points are labelled with the sample size for comparative purposes. Inferences are made about the trends by estimating values as if the sample sizes were the same at each data point, hence annual ‘increases’ and ‘decreases’ must be viewed relative to the sample size of an adjacent year. *Note*: international hunting operators are not based in South Africa and the lions are hunted in the country they are exported to. Results correspond to Question 39.

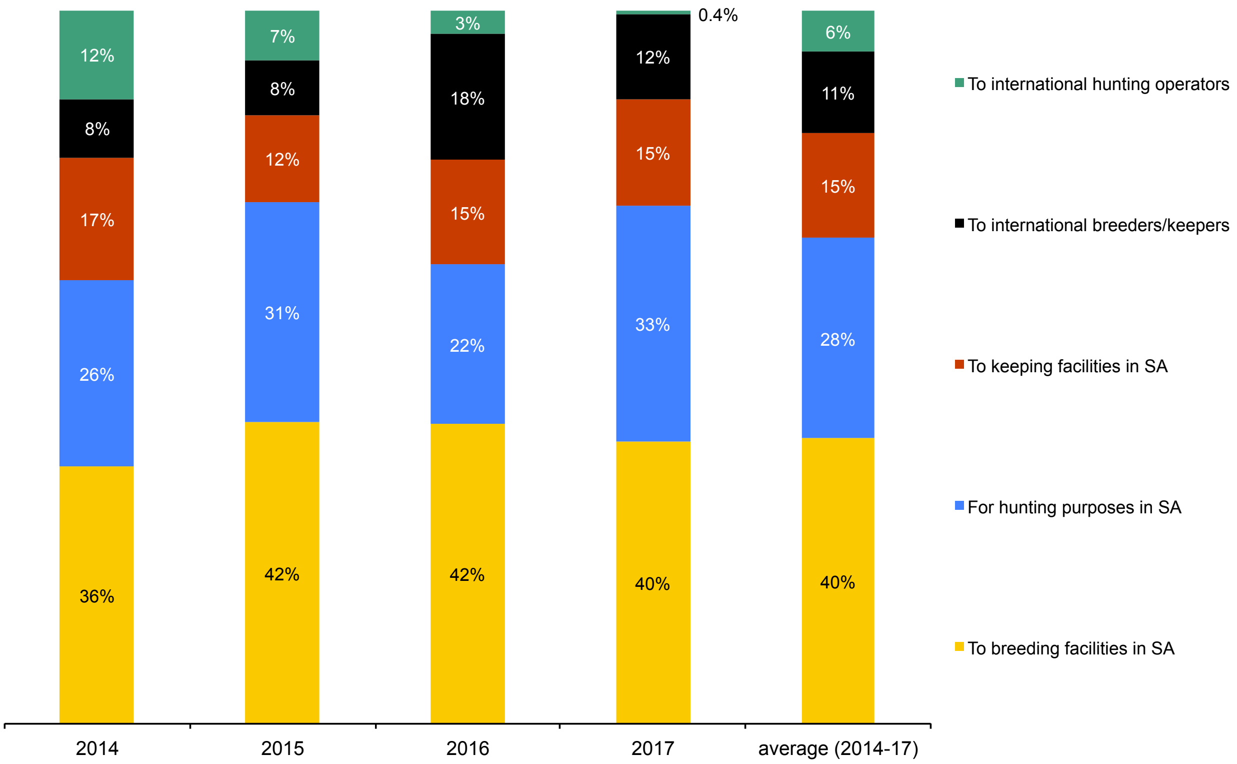

Figure H. Relative proportion of estimated live lion sales to five facility types from 2014 to 2017, and the average over that period. *Note*: international hunting operators are not based in South Africa and the lions are hunted in the country they are exported to. Results correspond to Question 39.

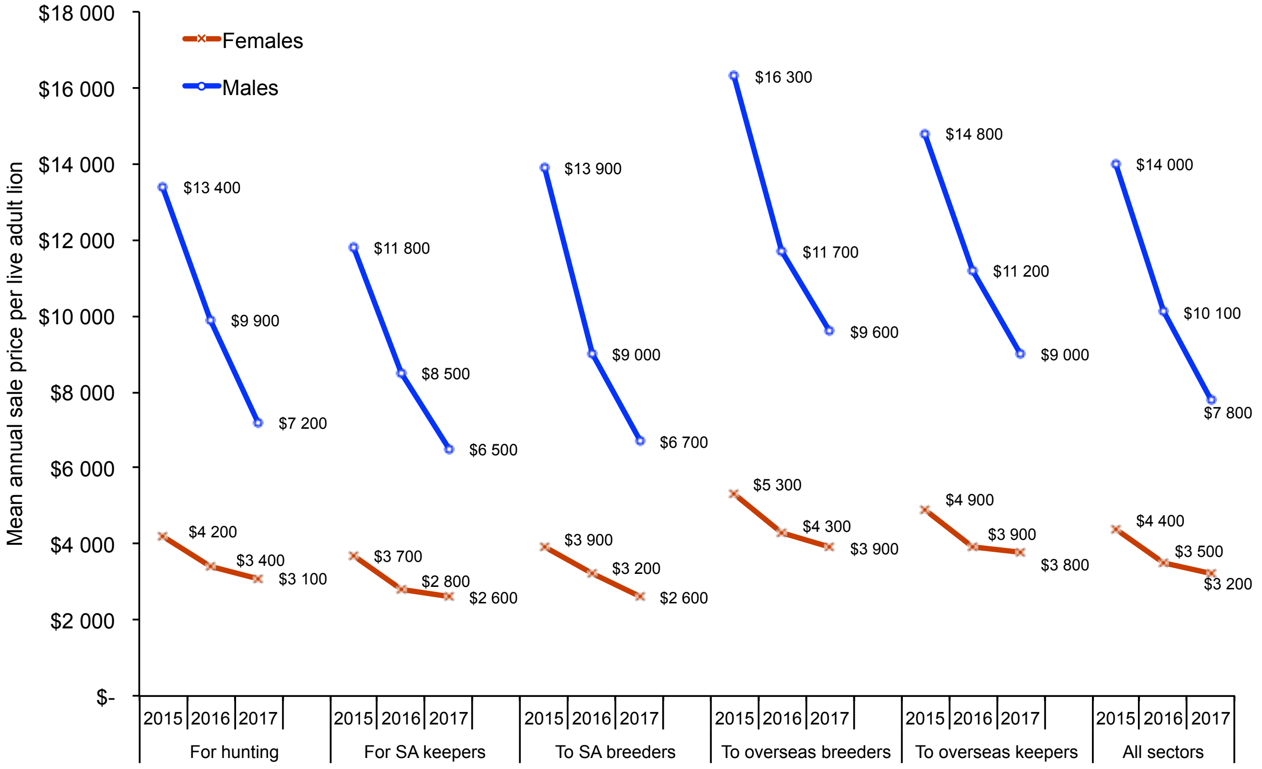

Figure I. The **mean** **USD annual sale price of LIVE ADULT lions per PURPOSE** (rounded up to the nearest USD100) (ZAR values in Fig 5). All means, medians, standard deviations and sample sizes are in Tables L & M of S1 Tables, and Figures J & K in S1 Figs. Mean annual ZAR:USD exchange rates: 2015 (1: 0.078); 2016 (1: 0.068); 2017 (1: 0.075). These are nominal figures (i.e. not inflation adjusted). Results correspond to Questions 41–43.

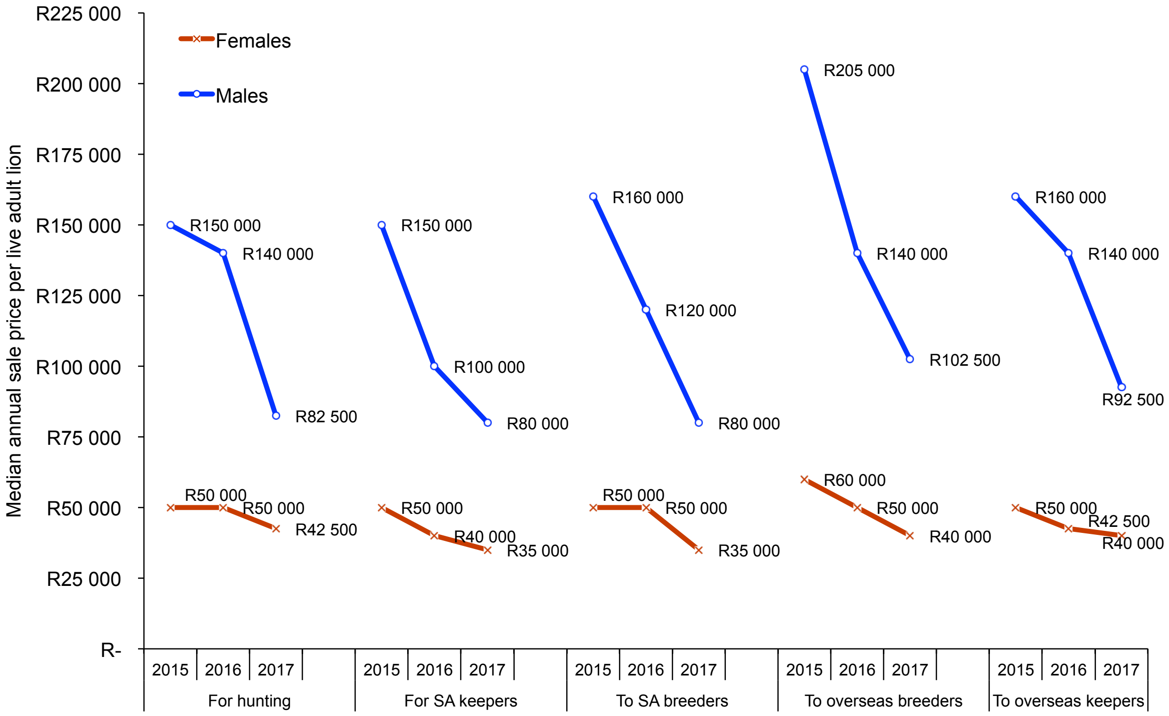

Figure J. **Median** **annual ZAR sale price of LIVE ADULT lions per PURPOSE** (rounded up to the nearest R100) (USD values in Figure K). Results correspond to Questions 41–43.

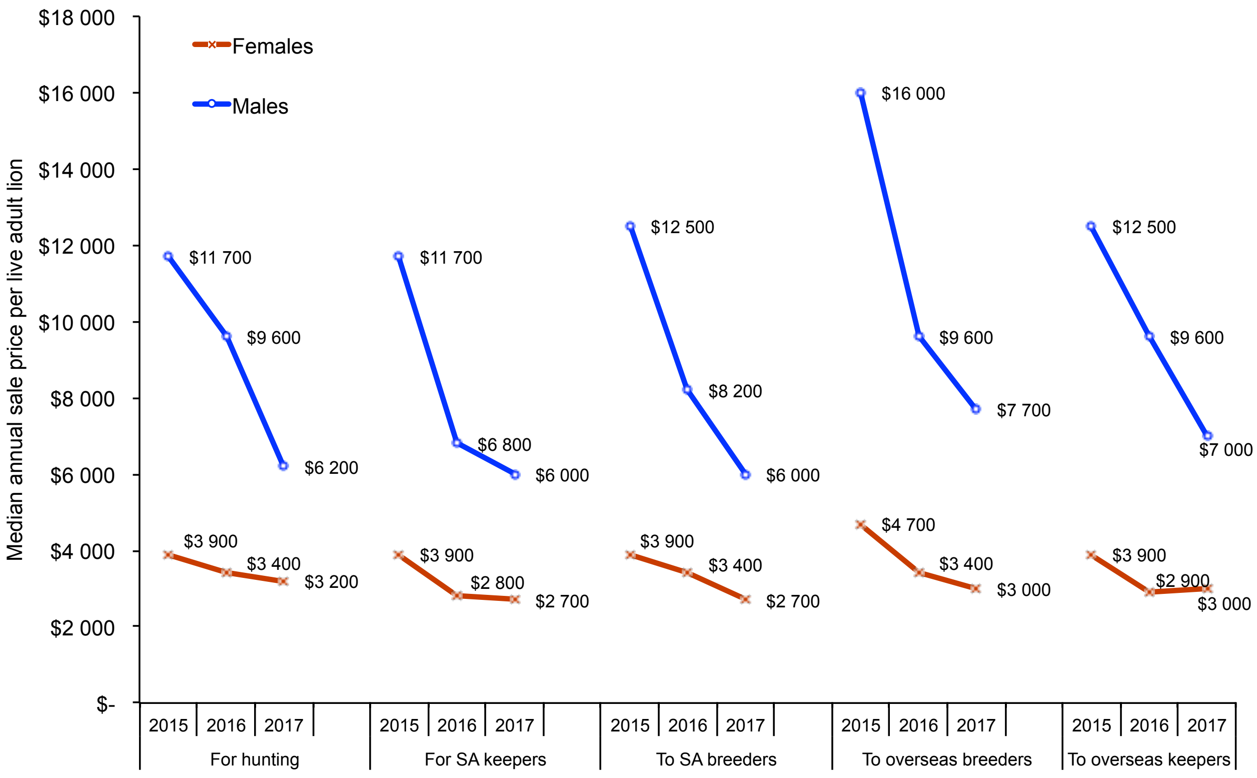

Figure K. Median annual USD sale price of LIVE ADULT lions per PURPOSE (rounded up to the nearest USD100) (ZAR values in Figure J). Mean annual ZAR:USD exchange rates: 2015 (1: 0.078); 2016 (1: 0.068); 2017 (1: 0.075). These are nominal figures (i.e. not inflation adjusted). Results correspond to Questions 41–43.

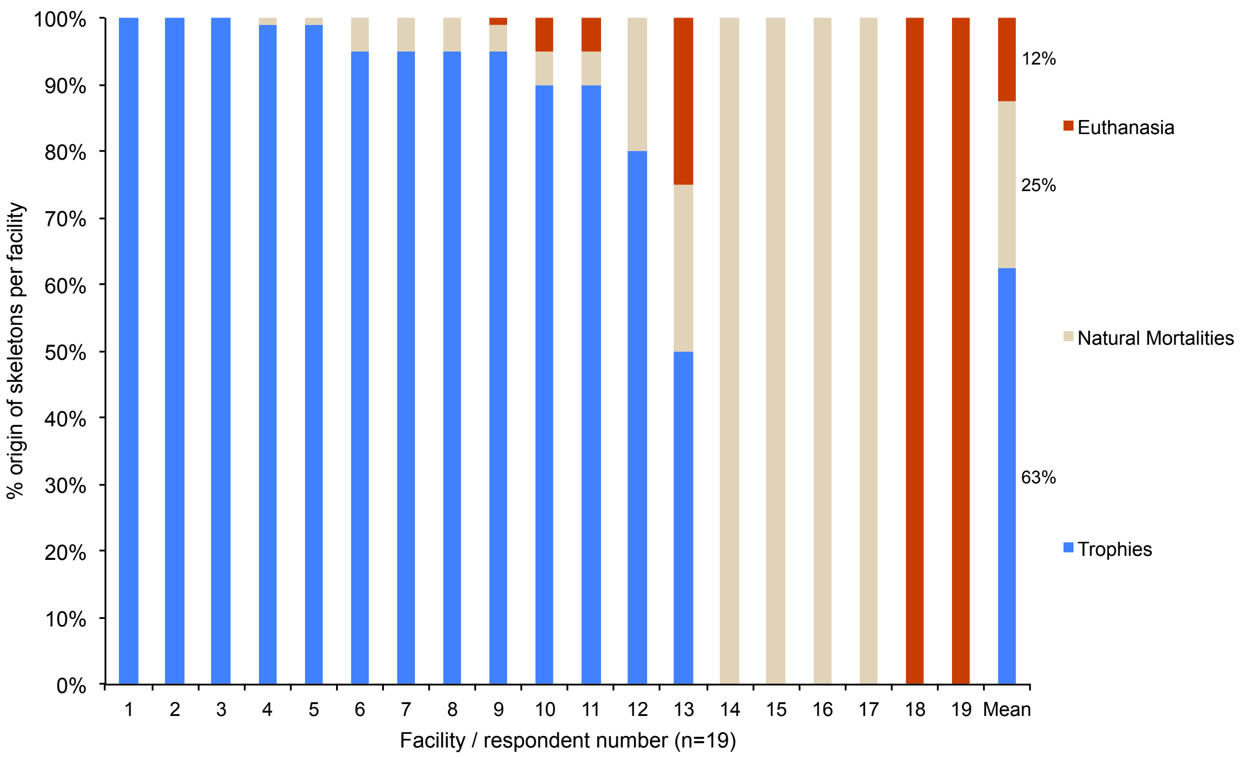

Figure L. Original answers from 19 facilities on the overall percentage origin of skeletons from different sources, and the mean. Of the 40 respondents, 19 chose to answer the question in this way (i.e. 'sum of the parts'), whereas 21 respondents interpreted this question in the way shown in Figure M. Results correspond to Question 48.

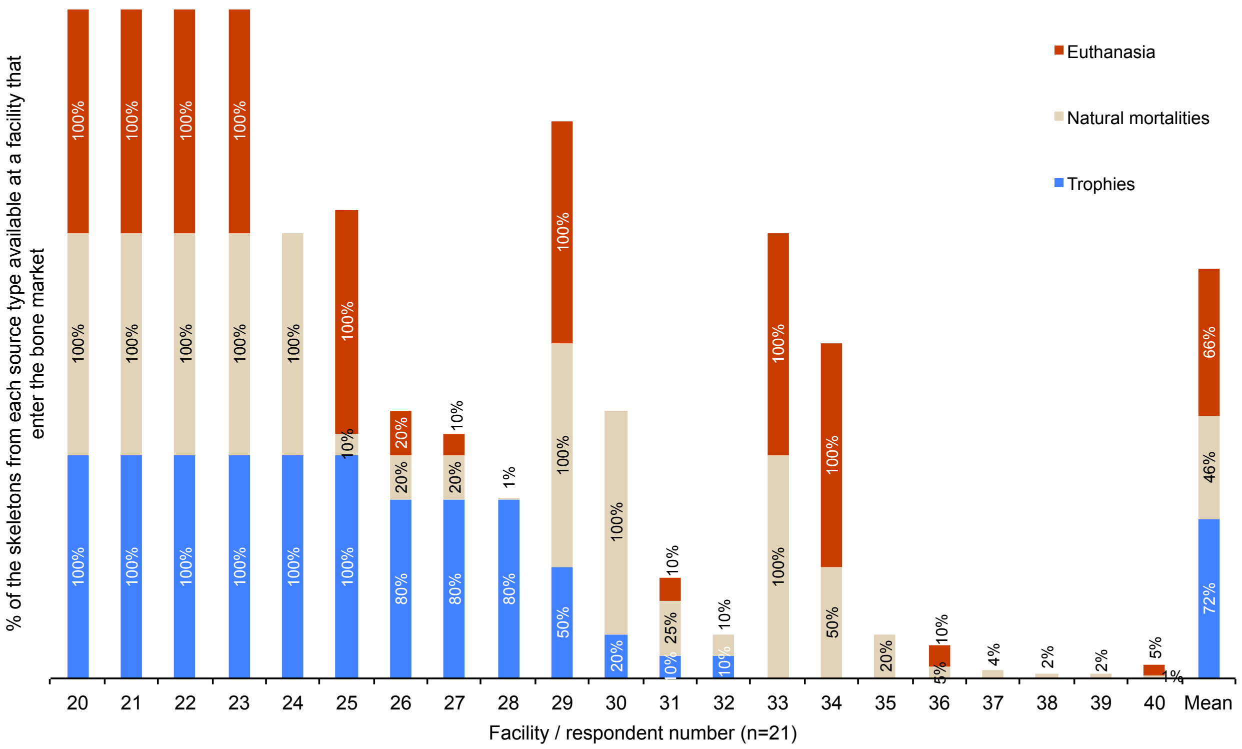

Figure M. Original answers from 21 facilities on the percentage of skeletons of a particular source type at a facility that are sold into the bone market, and the mean. Of the 40 respondents, 21 chose to answer the question in this way, whereas 19 respondents interpreted this question in the way shown in Figure L. Results correspond to Question 48.

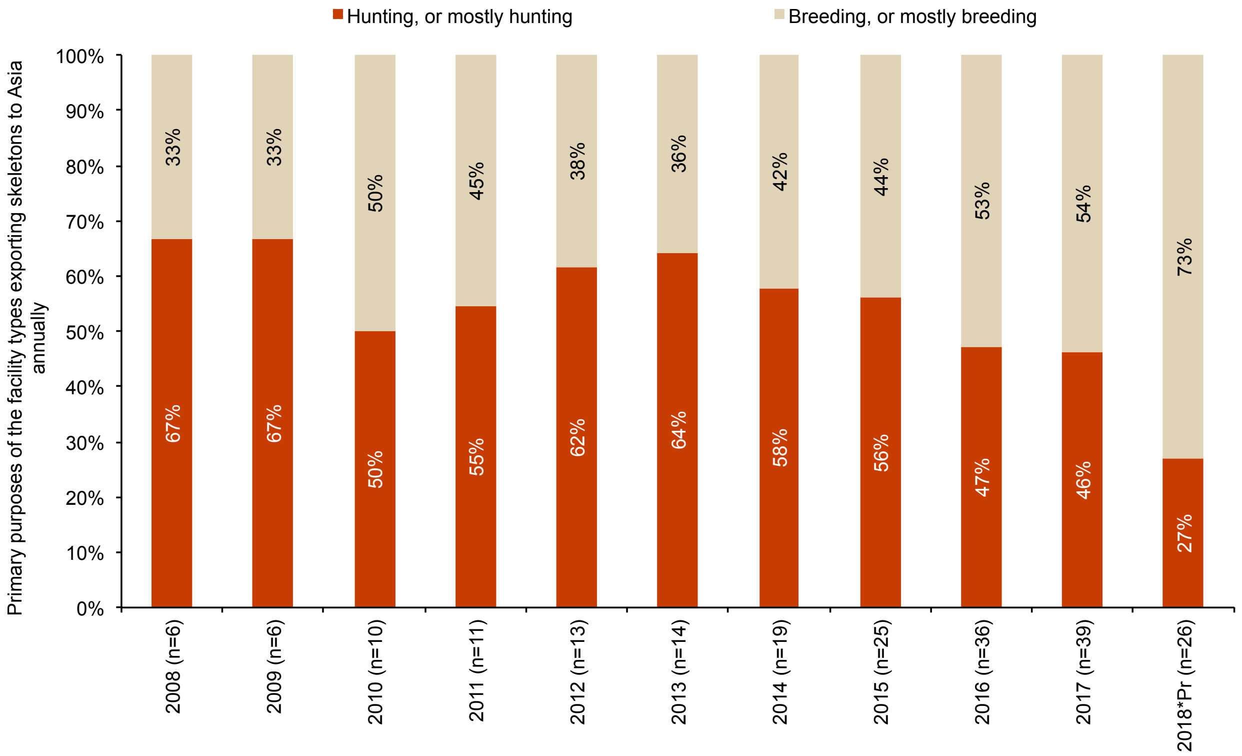

Figure N. Primary purposes of the facilities exporting skeletons to Asia annually, dichotomised as hunting (or mostly hunting) and breeding (or mostly breeding) (sample size, n, after the year). To dichotomise the purpose classifications, the ranked core purposes from Question 11 were used (along with all survey questions) to delimit whether dual-purpose facilities (i.e. those that breed and hunt, Fig 8) were 'mostly hunting' or 'mostly breeding'. Results correspond to Question 49.

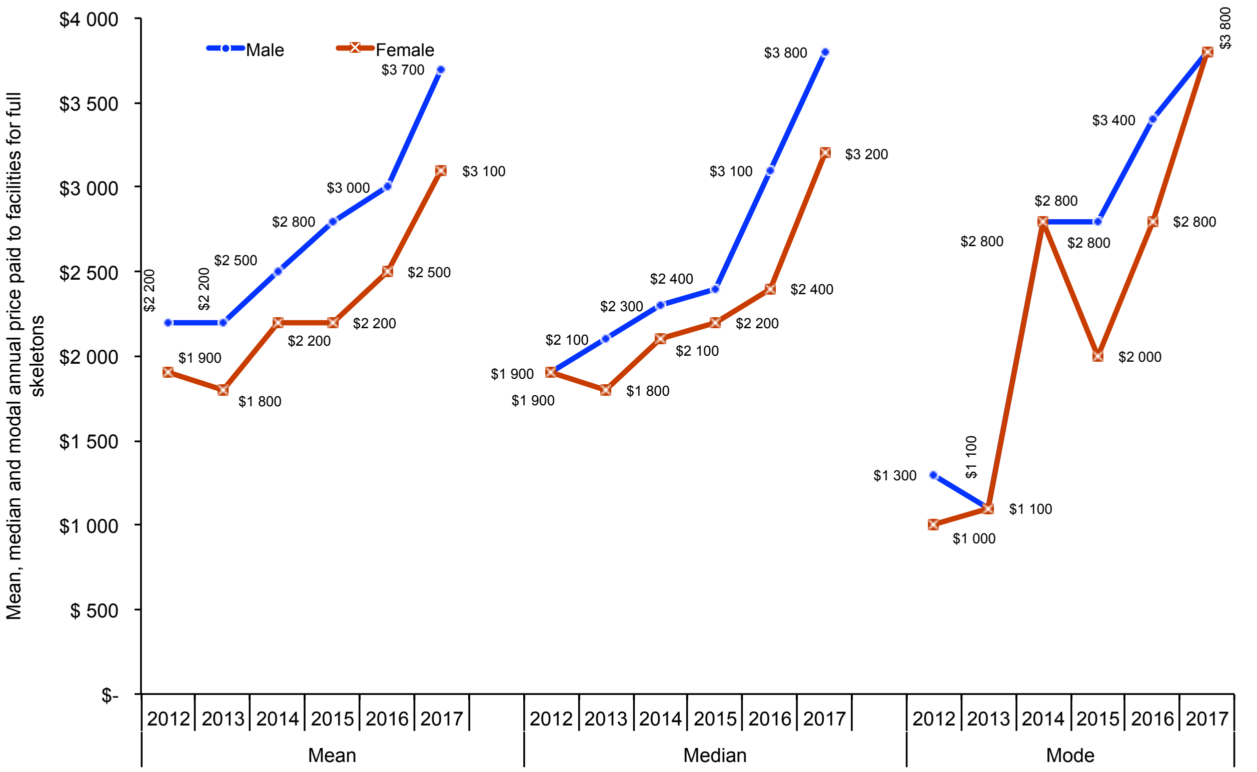

Figure O. Comparative annual mean, median and modal USD prices of MALE and FEMALE lion SKELETONS (2012–2017) sold by responding facilities (n=35) (mean annual prices, and sample sizes, for male and female lions in Figures Q–T. Graph showing values in ZAR in Fig 9, where mean ZAR:USD exchange rates: 2012 (1: 0.122); 2013 (1: 0.104); 2014 (1: 0.092); 2015 (1: 0.078); 2016 (1: 0.068); 2017 (1: 0.075). These are nominal figures (i.e. not inflation adjusted). Results correspond to Questions 50–51.

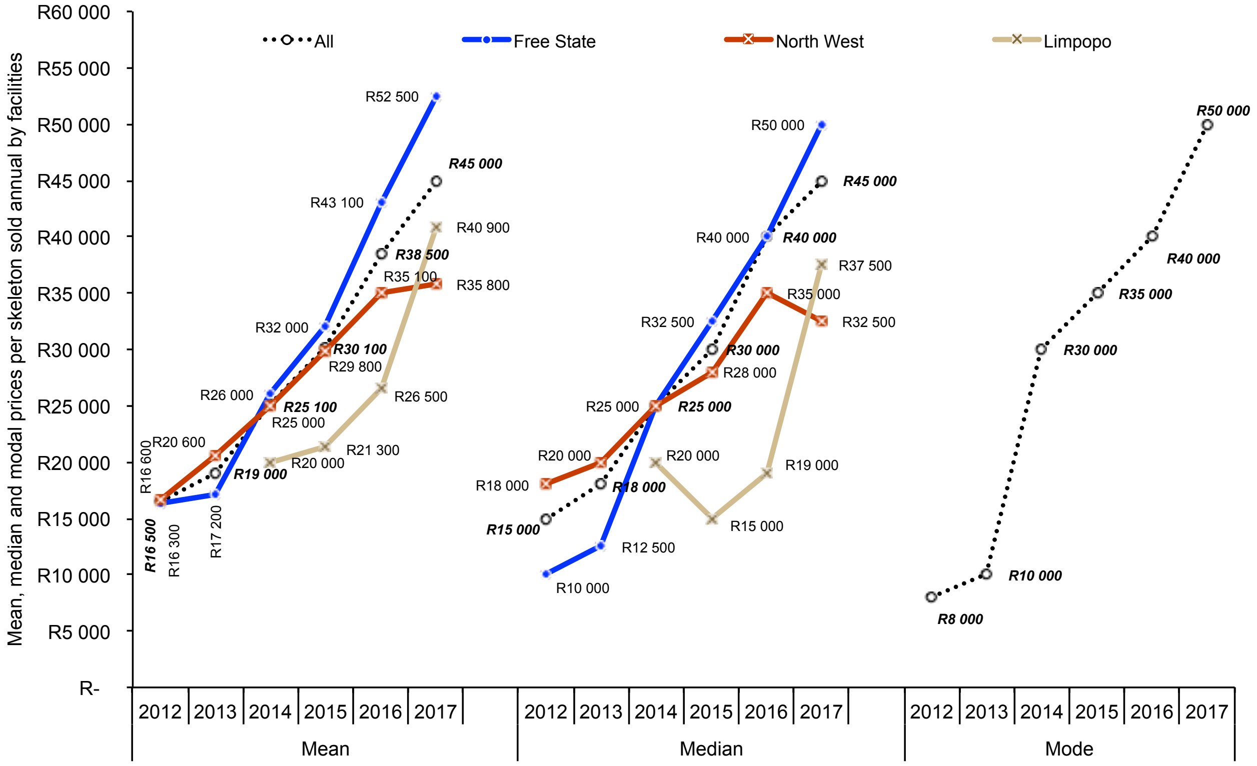

Figure P. Comparative annual mean, median and modal prices for lion SKELETONS, IRRESPECTIVE OF SEX, sold by responding facilities in three PROVINCES and overall (mean annual prices, and sample sizes, for male and female lions in Figures Q–T). Results correspond to Questions 50–51.

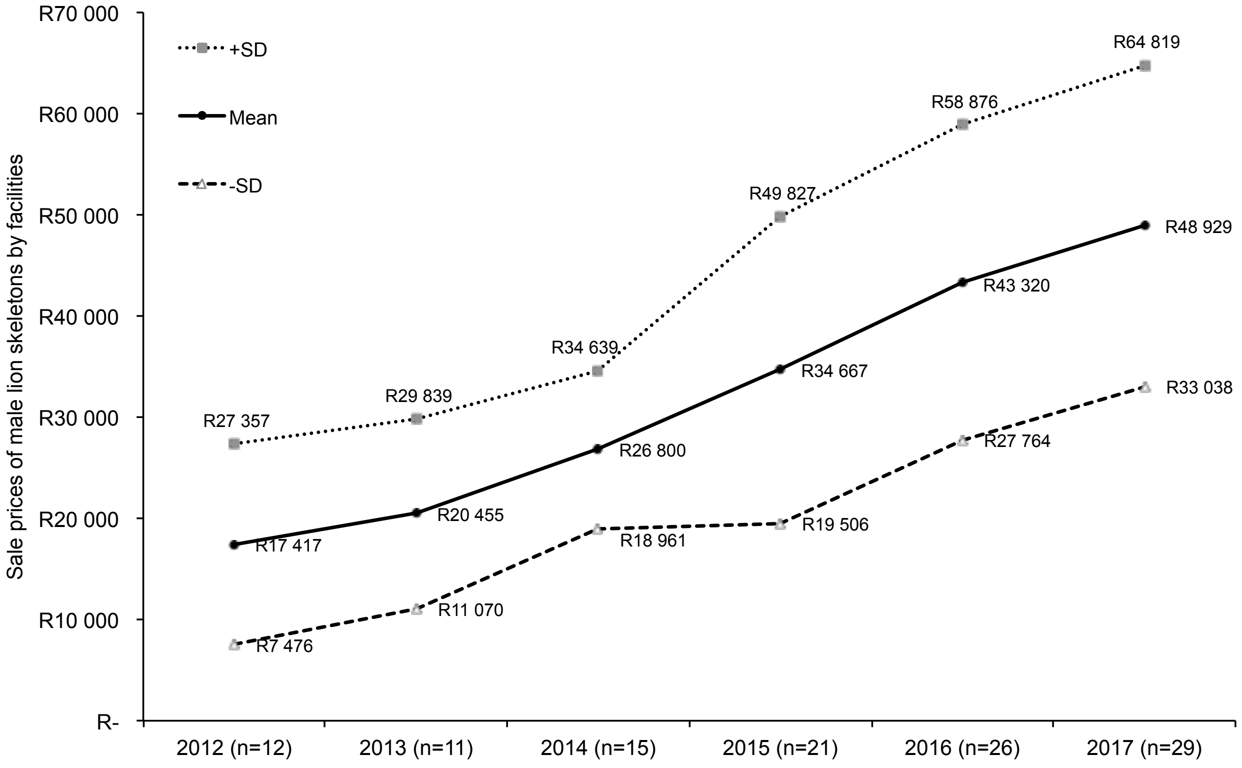

Figure Q. Prices of male lion skeletons (2012–2017) (mean ± standard deviation). Sample size (n) listed after the year. Y-axis the same as Figure R. Results correspond to Question 50.

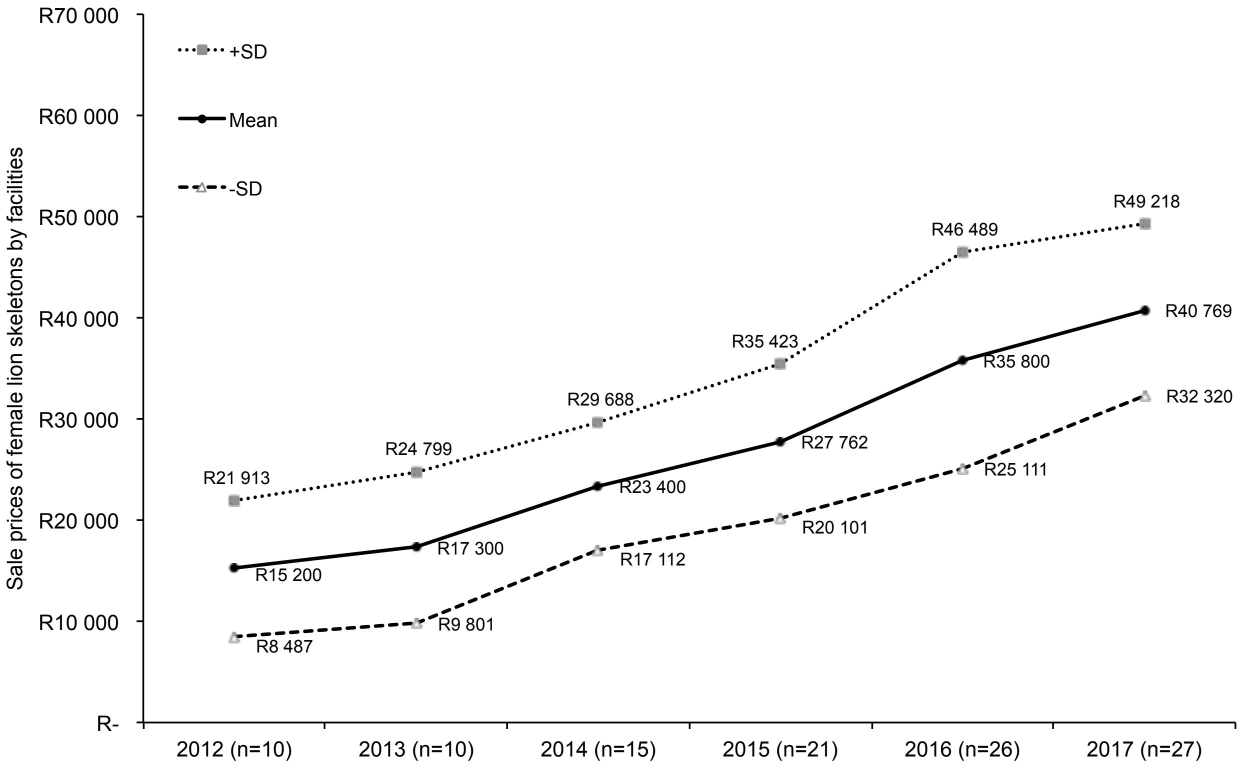

Figure R. Prices of FEMALE lion SKELETONS (2012–2017) (mean ± standard deviation). Sample size (n) listed after the year. Y-axis the same as Figure Q. Results correspond to Question 51.

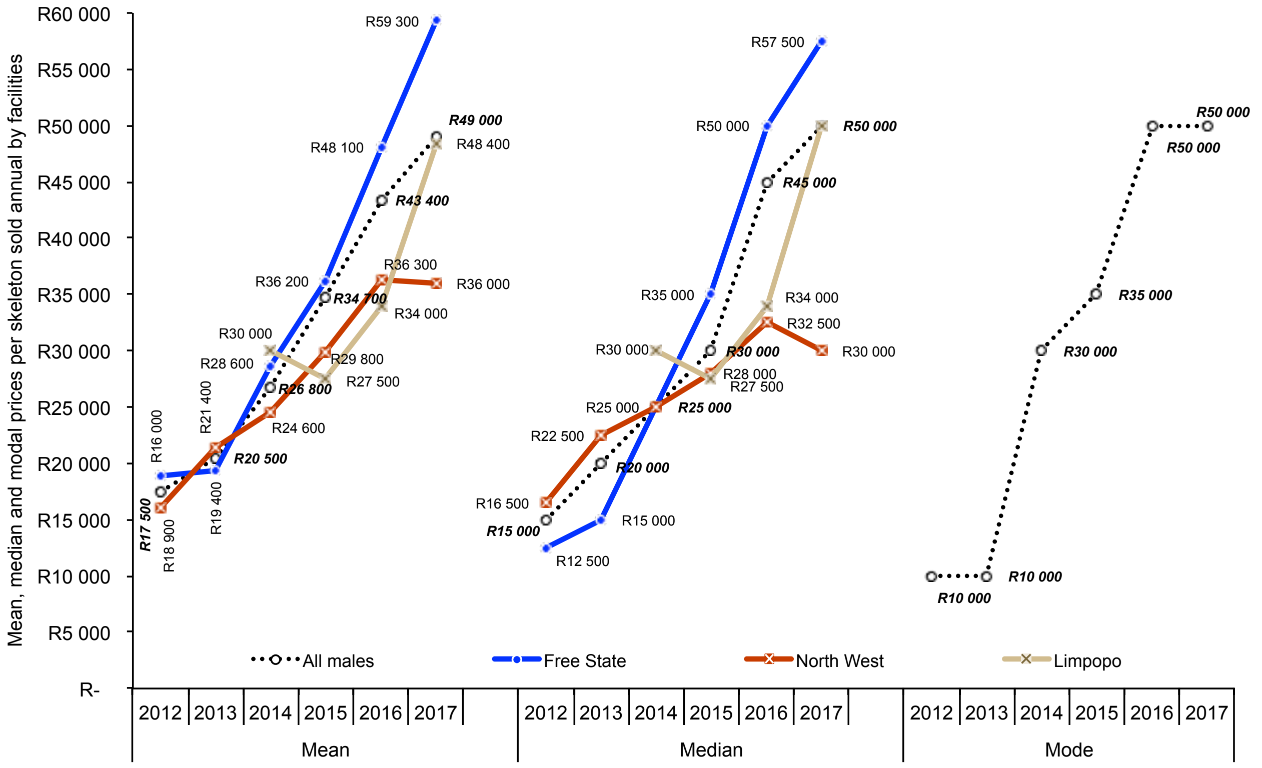

Figure S. Comparative annual mean, median and modal prices for MALE lion SKELETONS, sold by responding facilities in three PROVINCES and overall. Y-axis the same as Figure T. Results correspond to Questions 50.

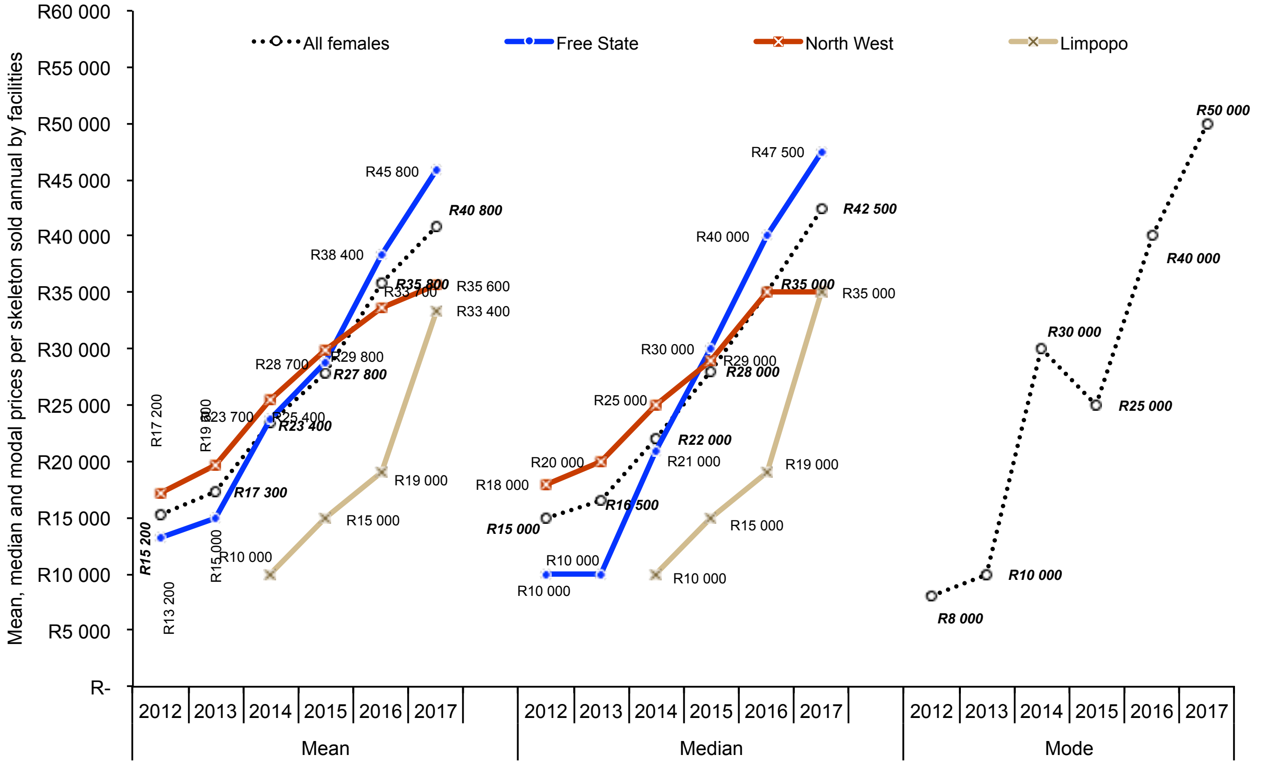

Figure T. Comparative annual mean, median and modal prices for FEMALE lion SKELETONS, sold by responding facilities in three PROVINCES and overall. Y-axis the same as Figure S. Results correspond to Questions 51.

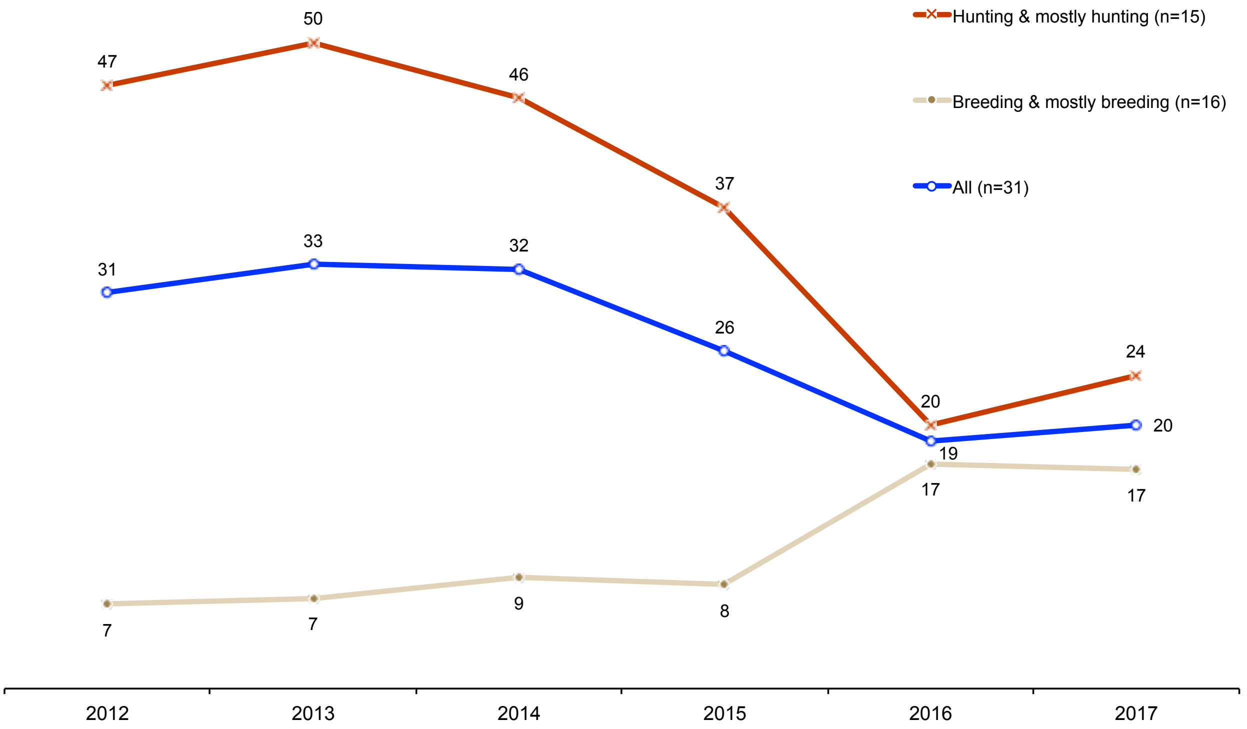

Figure U. Comparative annual mean number of skeletons sold by responding facilities according to the primary purpose of the facilities, dichotomised as hunting & mostly hunting and breeding & mostly breeding. (Annual sample sizes and standard deviations in Table N in S1 Tables). Results correspond to Question 52.
