# Supplementary material for: Born captive: A survey of the lion breeding, keeping and hunting industries in South Africa
Source: PLoS One. 2019 May 28;14(5):e0217409. doi: 10.1371/journal.pone.0217409 (PMC6538166; doi:10.1371/journal.pone.0217409)
Supplement: S1 Tables — (PDF) [file pone.0217409.s002.pdf]

S1 TABLES

Supplementary Tables A to O

Table A. Summary of the number of ToPS registered breeding and hunting facilities per province. The totals include lions of all ages and sexes. All information correct to late 2016. Excludes facilities that keep and/or display only. *Source:* the Department of Environmental Affairs.

Province	No. lion breeding facilities (no. lions)	No. lion hunting facilities (no. released for hunting c. 2016) [a]	Keeping facilities	Total breeding and hunting facilities [b]	Survey respondents	% responses in relation to the number of ToPS listed breeding and hunting facilities per province
**FS**	161 facilities (3784 lions)	No info	No info	161 facilities [c]	47	29%
**NW**	84 facilities (>3211 lions)	20 facilities (491 lions)	No info	104 facilities	45	43%
**LP**	28 facilities (>454 lions)	2 facilities (11 lions)	No info	30 facilities	16	53%
**GP**	15 facilities (152 lions)	No hunting of captive- bred lions	No info	15 facilities	5	33%
**EC**	7 (170 lions)	2 (135 lions)	No info	9 facilities	3	33%
**WC**	No captive breeding	No hunting of captive- bred lions	No info	0	1	?
**KZN**	2 (29 lions)	No hunting of captive- bred lions	No info	2	0	0%
**MP**	No captive breeding	No hunting of captive- bred lions	No info	0	0	-
**NC**	No captive breeding	No hunting of captive- bred lions	No info	0	0	-
**Total**	297 (7800 lions in breeding facilities) [d,e]	24 (637 lions released for hunting)		321	117	36% (n=321)

[a] These lions aren't included in the total captive lion numbers as they would likely be dead (or soon to be hunted) and thus not strictly part of a census on lions in captivity
[b] The total number of facilities excludes those smaller properties that keep (and not breed or hunt) a few lions. For example, the survey received one respondent from the Western Cape that had a 100ha property, of which 0.16ha for keeping and displaying lions. Hence this table excludes keeping only facilities
[c] Excludes the Free State hunting facilities, which number <10
[d] Minimum number in captivity since 11 breeding facilities in the data sent to us from DEA did not list the number of lions (2 facilities in North West, and 9 in Limpopo)
[e] Lions of all ages and sexes

Table B. Number of responding facilities and their membership to various associations. Results correspond to Question 6.

Organisation	No. (and %) of respondents who answered all/some of the questionnaire (n=117)
SAPA [a] only	62 (53%)
SAPA & PHASA [b]	18 (15%)
PHASA only	4 (3%)
PAAZA [c] only	3 (3%)
SAPA, PHASA, WRSA [d] & WTA [e]	1 (1%)
‘Other’ membership, not specified	9 (8%)
Not a member of SAPA, PHASA or PAAZA	20 (17%)

[a] SAPA = South African Predator Association.
[b] PHASA = Professional Hunters Association of South Africa.
[c] PAAZA = Pan African Association of Zoos and Aquaria.
[d] WRSA =Wildlife Ranching South Africa.
[e] WTA = Wildlife Translocation Association.

Table C. Reasons for why facilities breed and/or keep lions. Blank cells indicate reasons that were not free-listed by respondents in the ‘other’ category. Results correspond to Questions 9 (reasons for breeding) and 10 (reasons for keeping).

Reasons [a]	Reasons for breeding lions (n=98)	Reasons for keeping lions (n=104)
Live sales	n=76 (78%)	n=65 (63%)
Hunting	n=68 (69%)	n=71 (68%) [b]
Products/derivatives (e.g. skin, skeletons)	n=70 (71%)	n=17 (16%)
Skeletons/bones to Asia	Reason not a pre-listed option	n=33 (32%)
Tourism	n=25 (26%)	n=25 (24%)
Personal use/pleasure/purposes c	n=22(22%)	n=12 (12%)
Relocation purposes [d]	n=39 (40%)	Reason not a pre-listed option
Other: research/education	n=2 (2%)	n=3 (3%)
Other: love of lions	n=1 (1%)	n=1 (1%)
Other: security	n=1 (1%)	n=1 (1%)
Other: conservation of wild lion	n=1 (1%)	
Other: gene preservation	n=1 (1%)	
Other: sanctuary/rehabilitation		n=1 (1%) [e]
Other:	n=1 (1%) [f]	n=1 (1%) [g]

[a] The ‘other’ category was a free-listing option in Questions 9 & 10. In Question 11 (where respondents had to rank the core purposes of the facilities), an expanded set of pre-listed selectable options were provided – some of which where the same as the ‘other’ reasons cited in Questions 9 & 10. Hence, results for facility response numbers/proportions may differ in Table C and Fig 2.
[b] Keeping lions for hunting includes lions purchased from other breeders and kept for selling to hunting outfitters.
[c] Aware that there is a global trend for certain people to keep and even breed large carnivores as pets, we interpret this answer to represent such people. We anticipate that these owners are less motivated by commercial or financial considerations than regular commercial breeders.
[d] The questionnaire offered no guideline to respondents on how to interpret ‘relocation purposes’ for lions. The reason for including it as a response option was on advice received by SAPA that some farmers were known to breed and/or keep lions on an informal basis so that they could be relocated to areas where they could be free-roaming. After the survey, we followed up with respondents who chose not to remain anonymous to seek clarity on how they interpreted the category. Their understanding was also that people who buy live lions could relocate them to wherever they chose (which would include exports).
[e] ‘Other’ reason for keeping lions: “*we have one captive lioness due to her history as she could not be released back into the wild*”.
[f] ‘Other’ reason given for breeding lions: “*lions are free-roaming and are not a breeding project but an extensive system aimed at lions as a game farm animal* (sic)”.
[g] ‘Other’ reason for keeping lions: love of lions; “*lions as game farm animals to establish and utilise for hunting and ecotourism*”.

Table D. Reported number and percentage of adult male and female lions used for breeding during the survey period (August 2017 to May 2018) by 67 facilities, and facilities in three provinces. Results correspond to Question 33.

	Number of breeding adult lions					No. of facilities (n)
	Total reported	Mean per facility	Mode	Median	Range	
**All respondents** *(n=67 facilities; N=1418 adult breeding lions)*						
**Adult males**	511 (36%)	8±11	2	4	1 to 50	67
**Adult females**	907 (64%)	14±16	3	9	1 to 70	63
**Free State respondents** *(n=34 facilities; N=721 adult breeding lions)*						
**Adult males**	270 (37%)	8±11	5	5	1 to 50	34
**Adult females**	451 (63%)	15±12	20	13	2 to 60	31
**North West respondents** *(n=20 facilities; N=591 adult breeding lions)*						
**Adult males**	207 (35%)	10±13	2	4	1 to 40	20
**Adult females**	384 (65%)	19±23	3	8	2 to 70	20
**Limpopo respondents** *(n=8 facilities; N=63 adult breeding lions)*						
**Adult males**	21 (33%)	3±2	1	2	1 to 8	8
**Adult females**	42 (67%)	6±6	4	4	1 to 15	7

Table E. Methods employed by facilities to regulate lion numbers on their properties. Percentage of facilities selecting a method. Results correspond to Question 35

	Total responses (n=71)	Free State (n=36)	North West (n=22)	Limpopo (n=8)
**Selling**	65%	78%	59%	38%
**Separation of adults**	58%	47%	73%	63%
**Hunting**	35%	33%	41%	25%
**Euthanizing**	28%	44%	18%	-
**Natural mortalities**	24%	14%	41%	25%
**Fights**	13%	6%	23%	13%
**Contraception**	7%	-	14%	13%
**Sterilisation: males**	3%	3%	5%	-
**Sterilisation: females**	1%	-	5%	-
**Other (see footnotes)**	8%	11% [a]	5% [b]	-

[a] 'Other' answers supplied: (i) "*Euthanize adult lions for bone trade that are old enough for hunting, but which I can't sell*"; (ii) "Leave cubs with their mother"; (iii) "*It has never been necessary to use any of the above methods because we are only a small farm that recently started*"; (iv) "*Hand rearing has been stopped to lower costs and limit numbers*".

[b] Euthanasia for the bone trade.

Table F. Sources of lions used to stock the facilities when they opened, and sources of lions currently on the property. Multiple answers allowed. Results correspond to Questions 29 and 30.

Source type		Past [a]: source(s) of lions when facility was established [b] (n=100 respondents)	Present [c]: source(s) of lions currently on the property [d] (n=100 respondents)
Lions bred in this facility		-	67
Lions from captive breeders in South Africa		87	77
Lions from captive breeders elsewhere in Africa [e]		7	5
Lions from captive breeders elsewhere [e]		5	4
Wild-sourced lions from South Africa		5	2
Wild-sourced lions from elsewhere in Africa		-	-
Circuses		2	-
Do not know [f]		1	1
Other [g]	From respondents own breeding facility [h]	5	5
	From overstocked wild managed populations	1	1
	Europe	1	-
	Dropped off at a rehabilitation centre	1 [i]	1 [j]
	Zoo	-	2

[a] Sources of lions acquired when the facilities first opened between 1982 and 2017

[b] 90 respondents acquired lions from one source type when their facility was opened; five respondents acquired lions from two source types; four facilities acquired lions from three source types; and one facility acquired lions from four source types; 17 respondents did not answer the question.

[c] Lions on the property at the time of the survey, between August 2017 and May 2018

[d] 45 respondents have lions from one source type; 49 respondents have lions from two source types; three facilities each have lions from three and four source types respectively; 17 respondents did not answer the question.

[e] Source countries not divulged in Questions 29 and 30, but answers to Question 36 indicated that some lions were acquired from Botswana.

[f] Respondent with a hunting farm sources lions from a breeding facility and doesn't know their origins.

[g] 'Other': answers listed in the open-ended comment box

[h] Respondents with hunting farms who have an additional property (breeding farm) in a different province.

[i] Lioness was a tourist attraction at a lodge and she was dropped off at a rehabilitation centre.

[j] Cubs dropped off at rehabilitation centres for various reasons and given sanctuary.

Table G. Mean (±SD) (a) sizes of 99 properties and, depending on the province and farm purpose, the areas set aside for breeding, keeping, hunting and/or displaying lions, and (b) percentage of the total farm area set aside for the listed activities. Not every activity took place on the respondents’ properties. Results correspond to Question 13. (100 hectares equals 1km^2 or 0.39mi^2)

Activity	Eastern Cape (n=3)	Free State (n=38)	Gauteng (n=5)	Limpopo (n=13)	North West (n=39)	Mean ± SD [e]	Total area of farms
**(a) Mean area in hectares per farm**							
**Entire property (total area)**	*3167±1607 (n=3)*	*1810±2526 (n=38)*	*1296±1587 (n=5)*	*2662±1668 (n=13)*	*2594±2548 (n=39)*	*2229±2415 (n=99)*	*220655*
**Breeding [a]**	60±49 (n=3)	219±906 (n=39)	63±81 (n=2)	247±748 (n=11)	72±326 (n=27)	165±703 (n=82)	13528
**Keeping/rearing [b]**	60±49 (n=3)	430±1349 (n=34)	58±58 (n=3)	33±40 (n=12)	78±357 (n=31)	212±896 (n=84)	17767
**Hunting [c]**	4000±1414 (n=2)	4050±1907 (n=4)	-	1577±644 (n=5)	3287±2700 (n=22)	3163±2405 (n=33)	115090
**Display [d, f]**	-	67±153 (n=10)	56±53 (n=5)	170±410 (n=8)	23±26 (n=9)	78±21 (n=33)	2583
**(b) Mean % of farm area**							
**Breeding**	1.8±1.4% (n=3)	13.7±20.6% (n=37)	6.8±7.3% (n=2)	5.5±16.0% (n=11)	3.4±9.1% (n=27)	8.5±16.7% (n=80) [g]	
**Keeping/rearing**	1.8±1.4% (n=3)	13.7±19.0% (n=32)	6.0±5.4% (n=3)	1.0±1.2% (n=12)	3.3±8.6% (n=31)	7.0±14.0% (n=82) [g]	
**Hunting**	60% (n=1)	53.7±29.3% (n=3)	-	57.5±32.4% (n=5)	79.6±20.7% (n=21)	72.7±24.8% (n=30)	
**Display [h]**	-	5.1±5.9% (n=10)	6.3%±4.2% (n=5)	18.6±39.4% (n=6)	4.4±8.6% (n=8)	7.0±18.5% (n=30)	

[a] Total area set aside for breeding lions; some respondents listed the breeding area being the same area as the keeping and/or display area (see footnote f). Includes one facility in the Western Cape.
[b] Total area set aside for keeping and/or rearing/growing lions.
[c] Total area set aside for released lions to be hunted in.
[d] Total area set aside for lions to be displayed or viewed in (whether in cages, compounds or other public viewing types in properties described as sanctuaries, rehabilitation and rescue centres, predator parks, interactive tourism (e.g. petting), guest lodges).
[e] Includes one facility from the Western Cape.
[f] ‘Display area’ is mostly not an exclusive delimited area different to the breeding and/or keeping areas. Facilities sometime use breeding/keeping/hunting areas as viewing/display areas. In 15 of 33 responses (45%) the display area was a different delimited space to the breeding and/or keeping area. For 18 (55%) responses, the display area was the same as the breeding and/or keeping and/or hunting area (in 5 cases it was the same as the breeding area only; in 3 cases it was the same as the keeping area only; in 9 cases it was the same as the breeding and keeping areas; in one case it was the same as the hunting area).
[g] Two facilities did not list total property size, hence n values differ from property area above
[h] Includes properties described as sanctuaries, rehabilitation and rescue centres, predator parks, general tourism, interactive tourism (e.g. petting), guest lodges. See footnote ‘f’ on interpreting the display area.

Table H. Total proportion of lions listed by all responding facilities per age and/or sex group: (i) the mean per facility (this is the same as Table 4), and (ii) type of association with the hunting industry. The number of responding facilities differs annually, and 95 respondents provided data for ≥1 year. Results correspond to Questions 25–28.

31st Jan of the year [a]	% Adults (≥3 years old)		% Sub-adult (1–3 years old): any sex [d]	% Cubs (1 year old): any sex [e]	Total no. (any age or sex)	No. facilities (n=95)	Mean (±SD) number per facility:		
	Male [b]	Female [c]					All (any age/sex)	Adults (any sex)	Sub-adults & cubs (any sex)
(i) Annual captive numbers: all facilities									
**2015**	*29%*	*25%*	*29%*	*17%*	3697	58	64±79	35±48	29±42
**2016**	*29%*	*24%*	*31%*	*17%*	4567	70	67±79	35±46	32±43
**2017**	*30%*	*23%*	*30%*	*17%*	5769	83	70±86	37±54	32±46
**2018 [f]**	*30%*	*25%*	*28%*	*18%*	5389	62	87±114	48±67	39±69
**Mean**	*30%*	*24%*	*29%*	*17%*					
(ii) Annual captive numbers: type of association with the hunting industry									
**No association with hunting industry (n=25)**									
**2015**	*23%*	*24%*	*28%*	*25%*	556	21	26±32	12±13	14±23
**2016**	*23%*	*22%*	*33%*	*21%*	661	23	29±32	13±14	16±21
**2017**	*23%*	*23%*	*34%*	*20%*	639	23	28±32	13±14	15±21
**2018**	*23%*	*21%*	*35%*	*22%*	575	14	41±42	18±20	23±24
**Mean**	*23%*	*22%*	*33%*	*22%*			31±35	14±15	17±22
**Some association with the hunting industry (n=70)**									
**2015**	*31%*	*26%*	*29%*	*15%*	3141	37	85±90	48±55	37±48
**2016**	*29%*	*24%*	*30%*	*16%*	3996	47	85±89	46±52	39±48
**2017**	*32%*	*23%*	*29%*	*16%*	5130	60	86±95	47±61	39±52
**2018**	*31%*	*26%*	*27%*	*17%*	4814	48	100±125	56±73	44±77
**Mean**	*31%*	*25%*	*29%*	*16%*			89±100	49±60	40±56

[a] To 31 January of the respective years.
[b] Mean number of adult males per facility per year: *2015*=37; *2016*=37; *2017*=42; *2018*=50.
[c] Mean number of adult females per facility per year: *2015*=31; *2016*=31; *2017*=31; *2018*=42.
[d] Mean number of sub-adults per facility per year: *2015*=36; *2016*=40; *2017*=40; *2018*=46.
[e] Mean number of cubs per facility per year: *2015*=21; *2016*=22; *2017*=23; *2018*=30.

Table I. Total number of lions (minimum and maximum) listed by all responding facilities per age and/or sex group: (i) the mean per facility, and (ii) type of association with the hunting industry. The number of responding facilities differs annually, and 95 respondents provided data for ≥1 year. Results correspond to Questions 25–28.

31st Jan of the year [a]	Range in adult numbers (≥3 years old)		Range in sub-adult numbers (1–3 years old): any sex	Range in cub numbers (1 year old): any sex	Total no. (any age or sex)	No. facilities (n=95)
	Male	Female				
**(i) Annual captive numbers: all facilities**						
**2015**	1–150	1–100	1–200	1–70	3697	58
**2016**	1–200	1–100	1–180	1–50	4567	70
**2017**	1–300	1–139	1–200	1–100	5769	83
**2018**	1–300	1–188	1–200	1–180	5389	62
**(ii) Annual captive numbers: type of association with the hunting industry**						
**No association with hunting industry (n=25)**						
**2015**	1–35	1–30	1–40	1–50	556	21
**2016**	1–35	1–30	1–40	1–50	661	23
**2017**	1–33	1–28	1–45	1–30	639	23
**2018**	1–38	1–34	1–49	1–30	575	14
**Some association with the hunting industry (n=70)**						
**2015**	1–150	1–100	1–200	1–70	3141	37
**2016**	1–200	1–100	1–180	1–50	3996	47
**2017**	1–300	1–139	1–200	1–100	5130	60
**2018**	1–300	1–188	1–200	1–180	4814	48

[a] To 31 January of the respective years

Table J. Total proportion of lions listed by all responding facilities per age and/or sex group per province. The number of responding facilities differs annually, and 95 respondents provided data for ≥1 year. Results correspond to Questions 25–28.

31st Jan of the year [a]	% Adults (≥3 years old)		% Sub-adult (1–3 years old): any sex	% Cubs (1 year old): any sex	Total no. (any age or sex)	No. facilities (n=95)	Mean (±SD) number per facility:		
	Male	Female					All (any age/sex)	Adults (any sex)	Sub-adults & cubs (any sex)
**Free State (n=38)**									
**2015**	28%	22%	32%	18%	1942	21	92±98	46±56	46+58
**2016**	29%	22%	32%	17%	2279	29	79±85	40±53	38±48
**2017**	34%	22%	29%	16%	2314	34	68±66	38±53	30±31
**2018**	32%	26%	29%	12%	2585	32	81±84	47±70	34±42
**Mean**	*31%*	*23%*	*30%*	*16%*					
**North West (n=36)**									
**2015**	*32%*	*28%*	*26%*	*13%*	1491	21	71±71	43±51	28±21
**2016**	*28%*	*26%*	*29%*	*17%*	2050	24	85±86	46±47	39±44
**2017**	*27%*	*21%*	*33%*	*19%*	2838	30	95±112	46±59	49±66
**2018**	*24%*	*19%*	*30%*	*27%*	2126	20	106±162	46±65	61±105
**Mean**	*27%*	*23%*	*30%*	*19%*					
**Limpopo (n=12)**									
**2015**	*14%*	*25%*	*24%*	*37%*	87	9	10±8	4±2	6±6
**2016**	*28%*	*31%*	*28%*	*13%*	122	10	12±8	7±6	5±5
**2017**	*36%*	*38%*	*14%*	*12%*	375	11	34±75	25±62	9±14
**2018** [b]	*39%*	*37%*	*15%*	*9%*	537	6	90±102	68±84	22±34
**Mean**	*35%*	*36%*	*17%*	*12%*					
**Gauteng (n=5)** [c]									
**2015**	*36%*	*41%*	*8%*	*14%*	85	5	17±11	13±9	4±5
**2016**	*27%*	*32%*	*29%*	*11%*	106	5	21±16	13±10	9±9
**2017**	*29%*	*38%*	*20%*	*14%*	112	5	22±18	15±12	8±8
**2018**	*50%*	*50%*	*0%*	*0%*	6	1	6	6	0
**Mean**	*31%*	*37%*	*19%*	*13%*					

[a] To 31 January of the respective years.
[b] One respondent from Limpopo province reported captive lion numbers to January 2018 only (and omitted 2015–2017 numbers). The facility kept a higher than average number of lions, hence the averages and other values for 2015–2017 are lower than they would be had the respondent provided this information.
[c] Means are skewed by small sample size, especially 2018.

Table K. Estimated annual value of sales (ZAR), and the mean value per respondent, for income generating activities from lions from 2012 to 2017 (M=million). Sample sizes differ annually, hence the mean value per facility was calculated to evaluate annual trends. Mean annual ZAR:USD exchange rates: 2012 (1: 0.122); 2013 (1: 0.104); 2014 (1: 0.092); 2015 (1: 0.078); 2016 (1: 0.068); 2017 (1: 0.075); 2018 (1: 0.078). Results correspond to Questions 15 and 16.

		n	2012	2013	2014	2015	2016	2017
**Trophy hunting: foreign clients**	*Number of respondents*	25	14	16	17	22	22	23
	Total value of annual sales for facilities [a]		R37.5 M	R40.6 M	R51.3 M	R60.8 M	R30.0 M	R30.4 M
	Mean value of sales per facility [b]		R2.7 M	R2.6 M	R3.1 M	R2.8 M	R1.37 M	R1.32 M
**Trophy hunting: SA clients**	*Number of respondents*	17	8	8	11	13	12	13
	Total value of annual sales for facilities		R5.2 M	R3.0 M	R6.6 M	R7.4 M	R2.6 M	R2.9 M
	Mean value of sales per facility		R647 000	R372 000	R596 000	R568 000	R217 000	R224 000
**Live sales: trophy hunting**	*Number of respondents*	37	20	19	22	26	28	27
	Total value of annual sales for facilities		R20.73 M	R20.78 M	R25.6 M	R29.5 M	R15.3 M	R14.2 M
	Mean value of sales per facility		R1.04 M	R1.1 M	R1.17 M	R1.2 M	R546 000	R524 000
**Live sales: breeding**	*Number of respondents*	47	23	26	26	36	41	34
	Total value of annual sales for facilities		R15.6 M	R14.0 M	R13.5 M	R17.0 M	R20.5 M	R18.2 M
	Mean value of sales per facility		R677 000	R537 000	R518 000	R472 000	R500 000	R550 000
**Live sales: keeping**	*Number of respondents*	38	11	11	15	23	31	25
	Total value of annual sales for facilities		R2.9 M	R3.0 M	R3.1 M	R6.0 M	R6.9 M	R5.7 M
	Mean value of sales per facility		R259 000	R265 000	R206 000	R259 000	R221 000	R228 000
**Bone sales for export**	*Number of respondents*	44	18	19	23	32	38	33
	Total value of annual sales for facilities		R7.5 M	R7.3 M	R10.8 M	R22.1 M	R19.3 M	R17.8 M
	Mean value of sales per facility		R413 000	R383 000	R470 000	R691 000	R508 000	R537 000
**Display / tourism / education**	*Number of respondents*	13	10	11	12	13	12	12
	Total value of annual sales for facilities		R2.3 M	R2.3 M	R3.1 M	R3.6 M	R3.7 M	R3.5 M
	Mean value of sales per facility		R226 000	R206 000	R252 000	R272 000	R307 000	R290 000
**Muti**	*Number of respondents*	3	1	1	1	2	2	2
	Total value of annual sales for facilities		R80 000	R40 000	R40 000	R580 000	R580 000	R580 000
	Mean value of sales per facility		R80 000	R40 000	R40 000	R288 000	R288 000	R288 000

[a] Calculated per annum by multiplying the no. of respondents per sales value class (in ZAR) by the median value of the class (e.g. median of R22 500 for the ‘R15 000–R30 000’ class)
[b] Calculated as the mean for the values in the preceding row (e.g. for 2012, R27.5 million / 14, etc.)

Table L. The mean annual sale price of a live adult lion per purpose (values rounded to the nearest R100). For nominal (not inflation adjusted) USD values, the mean annual ZAR:USD exchange rates are: 2015 (1: 0.078); 2016 (1: 0.068); 2017 (1: 0.075). Results correspond to Questions 41–43.

		Total Responses	2015	2016	2017
**To SA breeders**	**Males**	42	R177,800 ± R86,500 (n=35)	R131,400 ± R70,800 (n=33)	R88,600 ± R36,200 (n=35)
	**Females**	40	R49,300 ± R19,300 (n=33)	R45,800 ± R19,600 (n=30)	R33,500 ± R13,600 (n=33)
**To SA keepers**	**Males**	30	R150,600 ± R71,900 (n=24)	R124,400 ± R72,200 (n=20)	R85,700 ± R40,400 (n=23)
	**Females**	30	R47,100 ± R14,600 (n=22)	R40,800 ± R16,100 (n=20)	R34,700 ± R12,000 (n=21)
**To international breeders**	**Males**	20	R208,100 ± R111,600 (n=8)	R170,600 ± R107,400 (n=8)	R128,000 ± R109,600 (n=8)
	**Females**	20	R66,900 ± R35,100 (n=8)	R63,200 ± R39,700 (n=8)	R51,300 ± R44,600 (n=8)
**To international keepers**	**Males**	20	R188,900 ± R107,600 (n=9)	R164,400 ± R107,000 (n=8)	R120,000 ± R109,400 (n=8)
	**Females**	20	R61,700 ± R34,700 (n=9)	R56,780 ± R45,000 (n=8)	R50,600 ± R44,800 (n=8)
**For hunting**	**Males**	39	R170,900 ± R58,800 (n=33)	R145,200 ± R78,200 (n=27)	R96,000 ± R40,500 (n=30)
	**Females**	36	R52,700 ± R13,600 (n=28)	R48,800 ± R14,600 (n=24)	R41,200 ± R10,900 (n=26)

Table M. The modal annual sale price of a live adult lion per purpose (values rounded to the nearest R100). For nominal (not inflation adjusted) USD values, the mean annual ZAR:USD exchange rates are: 2015 (1: 0.078); 2016 (1: 0.068); 2017 (1: 0.075). Results correspond to Questions 41–43.

		Total Responses	2015	2016	2017
**To SA breeders**	**Males**	42	R160,000 (n=35)	R120,000 (n=33)	R80,000 (n=35)
	**Females**	40	R50,000 (n=33)	R50,000 (n=30)	R35,000 (n=33)
**To SA keepers**	**Males**	30	R150,000 (n=24)	R100,000 (n=20)	R80,000 (n=23)
	**Females**	30	R50,000 (n=22)	R40,000 (n=20)	R35,000 (n=21)
**To international breeders**	**Males**	20	R205,000 (n=8)	R140,000 (n=8)	R102,500 (n=8)
	**Females**	20	R60,000 (n=8)	R50,000 (n=8)	R40,000 (n=8)
**To international keepers**	**Males**	20	R160,000 (n=9)	R140,000 (n=8)	R92,500 (n=8)
	**Females**	20	R50,000 (n=9)	R42,500 (n=8)	R40,000 (n=8)
**For hunting**	**Males**	39	R150,000 (n=33)	R140,000 (n=27)	R82,500 (n=30)
	**Females**	36	R50,000 (n=28)	R50,000 (n=24)	R42,000 (n=26)

Table N. The respondents’ answers to quantities of skeletons exported: (i) how many skeletons from their facility were exported to Asia in 2012–2017, and (ii) an estimate of how many skeletons the facility could export to Asia in one year of there were no restrictions, and comments on how the estimate was calculated. Results correspond to Questions 52–53.

Facility no.	Number of skeletons exported (Q52) (n=31)						Number of skeletons that could be exported without restrictions (Q53)	How the estimate was calculated by the respondent (Q53)
	2012	2013	2014	2015	2016	2017		
F1							<5% of stock	Facility not focused on bone market. Natural mortality (<5%) will be available to the market, which will help a lot
F2							2–3	Natural mortalities of lions that have killed each other
F3							8	Lions not suitable for breeding
F4							10	-
F5							20	
F6							20	
F7							70	I need to reduce my lion numbers. I need to support my income from the lions, otherwise my lion business will run at a loss.
F8							80	To maintain a stable population my population increase needs to be balance with a population decrease. I could do this balance via the hunting market, but due to the restrictive measures on the hunting market I had to support the bone market with skeletons. If no hunting market exists, I need to have an "outlet" for my products, and the bone market will be an obvious choice. I can produce approximately 60 to 80 lion cubs per year, thus would need a market for about 60 to 80 animals per year.
F9							80	This is the number of small lions that we breed per year
F10							100	Because there were too few sold in the previous year, the total built up.
F11							300	An assessment based on a combination of bred lions and purchased animals
F12						4	10–15	Depending on the hunting trade
F13						5	80	Production
F14						10	15	Currently having stock
F15						10	15	-
F16						11	50	Current breeding stock, and sub-adult animals reaching adulthood, but no hunting opportunities
F17						43	50	I am producing between 40 and 60 lion cubs per year.
F18						43	50	I can export about 50 on a yearly basis
F19					2	3	4	-

**F20**				3	9	5	10	Only hunted carcasses
**F21**				3	10	15	30	Based on the number of adult lions not hunted that are older than 3 years and ‘right’ to hunt
**F22**				45	35	35	55	USA market will make the count higher
**F23**					80	80	250	-
**F24**					2	4	5–7	-
**F25**				1			0	-
**F26**				2	3		10	-
**F27**				5	8	10	See comment	Normally I will think as a breeding farm and 10% of my lions can be exported as bones – these will be lions that have died by chance, or those that I want to euthanize because they may not be right for breeding. This is only if the hunting market is open. If the hunting market is closed, then the numbers can increase 50% due to limited space.
**F28**				20	40	40	50	If I breed full out, it is half of the increment
**F29**			1	5	10	15	20	Because I am going to hunt 20 this year
**F30**			5	10	15	15	30	-
**F31**			10	2	2	3	24	8 lionesses x 3 cubs = 24. This will also happen over time. 1 lioness produces 3 litters over 2 years. Therefore over a period of two years 74 will be feasible.
**F32**			40	58	54	42	60	If there are no restrictions: can hunt an average of 60 lions per year
**F33**	2	4	4	4	4	6	6	Lions that are too old to breed, and injured lions
**F34**	4	4	4	6	8		30	With the current hunting market decreasing, there has also been a reduction in the sales of breeding animals – i.e. we are now obliged to sell 40-60% of our lion stock in to other markets such as bones. With my current breeding, I have to sell about 30 carcasses
**F35**	10	10	10	10	15	15	25	This is what my facility allows – to produce 25 lions for the bone market older than 3 years. Food is a limiting factor.
**F36**	10	10	20	25	30		30	-
**F37**	30	40	60	44	5		40–80	This is the number of lions we can comfortably hunt every year if there were no restrictions.
**F38**	40	45	50	55	34	22	100	If the USA market opens up, I will be able to export 100 skeletons with the European market
**F39**	40	50	60	60	4		5–10	In 2016 we only hunted 5 lions, compared to 50 in 2015. Because the import of lions into the USA is closed, we only hunt between 5–10 lions per year.
**F40**	50	50	50	50	50	50	?	Do not know
**F41**	50	50	60	60	5	5	5	-
**F42**	70	65	80	80	14	20	70–90	Average 70–90 based on current bookings from USA clients if the USFWL (US Fish and Wildlife) open hunting and bookings from Middle East clients China and Poland.

	2012	2013	2014	2015	2016	2017	***SUMMARY***
**Total skeletons (SKE) exported**	306	328	454	548	439	511	*>1892*
% of actual exports			*51%* [a]	*71%* [a]	*25%* [a]	*64%* [a]	
**Mean SKE facility^{-1} yr^{-1}**	31	33	32	26	19	20	*49*
**±SD**	23	23	27	26	21	19	61
**No. respondents (n)**	10	10	14	21	23	25	42

[a] Actual exports in 2014, 2015 and 2016 were 889 skeletons, 777 skeletons and 1771 skeletons respectively [Ref 1]). Exports in 2017 were 800 according to the quota

Table O. The estimated mean percentage (±SD) of hunting clients per facility from countries and regions before and after the January 2016 U.S. suspension on the import of captive-origin lion trophies, and the mean percent difference per facility. Results are ordered from left to right based on the bottom row (i.e. from most to least negative and positive change in proportion of clients) (n=33). Results correspond to Questions 57–58.

	USA	United Kingdom	Middle East	Canada	South Africa	Australia	Asia	Other Europe	Other (not specified)*
**Before Jan. 2016**	73±25%	6±5%	9±13%	7±5%	33±39%	4±1%	7±11%	11±8%	15±7%
**After Jan. 2016**	42±35%	8±4%	14±14%	14±8%	37±36%	10±10%	20±28%	35±30%	46±37%
**Mean % difference per facility**	-34±46%	1±2%	1±7%	3±7%	4±18%	7±13%	11±26%	21±31%	33±38%

* Includes Russia and Croatia
