# Supplementary material for: Born captive: A survey of the lion breeding, keeping and hunting industries in South Africa
Source: PLoS One. 2019 May 28;14(5):e0217409. doi: 10.1371/journal.pone.0217409 (PMC6538166; doi:10.1371/journal.pone.0217409)
Supplement: S1 File — (PDF) [file pone.0217409.s003.pdf]

PDF version of NATIONAL CAPTIVE LION SURVEY (South Africa) (V2: REVISED FOR 2018)

Welcome to the Survey

OVERVIEW

In accordance with the annotation to the Appendix II listing for the African Lion adopted at the 17th Conference of the Parties to CITES (CoP17) in October 2016, South Africa is required to establish an export quota for lion bones. The South African Scientific Authority is mandated to advise the Department of Environmental Affairs (DEA) on the size of the quota on an annual basis.

In order to provide sound scientific decision support to the DEA, a research project has been initiated by the South African Scientific Authority with the following aims to: (1) increase our understanding of the captive lion breeding industry and trade in lions (especially bones, but also other products and live lions) in South Africa; (2) investigate the trade in captive produced lion bones under a quota system; (3) gain a better understanding of the consequences of the US ban on imports of captive-origin trophies; and (4) strengthen the evidence base for the annual review of the lion bone export quota.

INVITATION

We hope you will take part in this study by completing this questionnaire. **You do not have to answer all the questions,** but we would appreciate you answering as many questions as honestly as possible. If there are certain activities that the facility does not do (e.g. hunting), then you will be automatically redirected to a different set of questions.

The **questionnaire can be completed anonymously**; however, even if you do choose to reveal your name, then neither your name nor that of the facility will appear in any of the publications or reports resulting from this survey.

The survey will remain open until a date to be confirmed in 2018. The survey can be completed in Afrikaans (Link to survey in Afrikaans).

If you received an invitation from SAPA (South African Predator Association) to participate, then the hardcopy of this survey is available from SAPA. SAPA has agreed to capture these survey responses electronically, thereby guaranteeing further anonymity. The original questionnaires returned to SAPA will not be copied to us.

Submission of the questionnaire will be taken as your consent for us to use the information you provide.

NOTE: If you experience a technical problem with a question, please will you inform us afterwards which number it is.

Should you have any queries or concerns about the research, please contact any member of the collaborating team:
* Dr Vivienne Williams (vivienne-ncls@wildscience.co.za) University of the Witwatersrand (Primary researcher)
* Mr Michael 't Sas-Rolfes tsas.rolfes@gmail.com) Independent researcher / University of Oxford
* Ms Michele Pfab (M.Pfab@sanbi.org.za) SANBI

Ethics Clearance No.: H17/06/55 (Universiteit van die Witwatersrand)

* 1. **Please note before you start: several broad questions will be asked about the income generated from lion sales (live, products, trophies, etc). In the interests of giving us the correct information when responding to these important questions, please consider the option of remaining anonymous IF it means that you are more likely to provide an accurate answer. If you are in anyway unsure, rather remain anonymous. All information will be used for analytical purposes only and will be treated as confidential.**

- ◯ I prefer to remain anonymous
- ◯ I do not mind identifying myself and/or the facility/business

PDF version of NATIONAL CAPTIVE LION SURVEY (South Africa) (V2: REVISED FOR 2018)

SECTION A: Participant Information

2. Facility

Respondent name

Facility name

* 3. Province the facility is in?

* 4. The person answering this questionnaire is:

- The owner of the facility
- An employee of the facility
- Other (please specify)

PDF version of NATIONAL CAPTIVE LION SURVEY (South Africa) (V2: REVISED FOR 2018)

SECTION B: Facility Information

5. What year did the facility open?

[ ]

* 6. Is the facility SAPA* accredited and/or a member of PHASA** and/or a member of PAAZA***?

** South African Predator Association; ** Professional Hunters Association of South Africa; *** Pan African Association of Zoos and Aquaria*

- [ ] SAPA accredited
- [ ] PHASA member
- [ ] SAPA & PHASA
- [ ] PAAZA
- [ ] None of these
- [ ] Other (please specify)

* 7. Purpose of the facility? (select all that apply)

- [ ] Breeding
- [ ] Keeping
- [ ] Hunting
- [ ] Display
- [ ] Other (please specify)

8. Number of people employed at the facility?

* 9. Reasons for breeding (select all that apply)

- [ ] Breeding for live sales
- [ ] Breeding for relocation purposes
- [ ] Breeding for hunting
- [ ] Not applicable
- [ ] Breeding for products/derivatives (incl. bones)
- [ ] For tourism
- [ ] Breeding for personal use/pleasure
- [ ] Other (please specify)

* 10. Reasons for keeping (select all that apply)

***** *'Keeping' for hunting includes lions purchased from other breeders and kept for selling to hunting outfitters*

- [ ] For hunting*
- [ ] For personal use/purposes
- [ ] For 'muthi'
- [ ] For skeletons/bones to Asia
- [ ] For live sales
- [ ] Not applicable
- [ ] For products/derivatives (e.g. skins)
- [ ] For tourism
- [ ] * Other (please specify)

11. Rank the core purpose(s) of the facility, starting from 1=most important.(select as many apply)

Breeding and rearing

Hunting safaris

Lion bone sales

Zoological garden

Predator park

Education facility

General tourism

Interactive tourism (e.g. petting, walking with lions)

Live sales

Rehabilitation centre

Sanctuary / rescue

Guest lodge with wedding/conference venue

Conservation facility

Other

12. Regarding paying visitors*: (* e.g. hunters, clients, tourists, scholars, etc)

On average, how many does the facility get per year?

Is the number of visitors increasing, decreasing or stable?

13. What is the approximate size of: (specify whether in units of ha, km^2, m^2)

the entire property

[ ]

the total area set aside for breeding lions

[ ]

the total area set aside for growing and/or keeping lions

[ ]

the total area set aside for released lions to be hunted

[ ]

the total area set aside for display

[ ]

14. From January 2016, did:

	Increase	Decrease	Stay the same	N/A
The total number of lions on the property:	○	○	○	○
The total breeding area:	○	○	○	○
The total keeping and/or growing area:	○	○	○	○
The total hunting area:	○	○	○	○

15. Estimated annual value of sales (1): indicate per activity from the drop down options:

NB: We would ideally like data going back to 2012. However, if you can't remember figures for some of those years, we would strongly encourage that you at least provide estimates for 2015 to present, and omit what you don't recall.

	Live sales for breeding	Live sales for trophy hunting	Trophy hunting on the property: international clients (packages)	Trophy hunting on the property: SA clients
2012				
2013				
2014				
2015				
2016				
2017				

Other (please specify)

[ ]

16. Estimated annual value of sales (2): indicate per activity from the drop down options:

NB: We would ideally like data going back to 2012. However, if you can't remember figures for some of those years, we would strongly encourage that you at least provide estimates for 2015 to present, and omit what you don't recall.

	Live sales for keeping	Bone/skeleton sales for export market	Skin/body part sales for 'muti'	Display/ tourism / educational visits
2012				
2013				
2014				
2015				
2016				
2017				

Other (please specify)

17. Did the January 2016 US ban on the import of captive-produced lion trophies impact your business in any way?

- ◯ Yes
- ◯ No
- ◯ N/A

18. Since you answered 'Yes', please indicate how you are adapting to the impact (select all that apply)

- ☐ We have scaled down breeding production
- ☐ We have redirected the business to focus on the lion bone trade
- ☐ We have redirected the business to focus on interactive tourism
- ☐ We have had to let go of employees/workers
- ☐ Other (please specify)
- ☐ We have sold off live lion stock
- ☐ We have euthanised lions
- ☐ We have continued business as usual

19. If the US ban continues to be implemented with no sign that it will be lifted in the near future, we will (select all that apply)

- ☐ Convert the business to another form of wildlife breeding
- ☐ Close the business
- ☐ Focus on the lion bone trade
- ☐ Focus on interactive tourism
- ☐ Continue business as usual
- ☐ Euthanise all lion stock
- ☐ Other (please specify)

20. In addition to the US ban, if the UK and/or Europe also implements a ban on an the import of lion hunting trophies, we will: (select all that apply)

- ☐ Convert the business to another form of wildlife breeding
- ☐ Close the business
- ☐ Focus on the lion bone trade
- ☐ Focus on interactive tourism
- ☐ Downscale, but continue production expecting that the US bans will lift in the near future
- ☐ Euthanise all lion stock
- ☐ Continue business as usual
- ☐ Other (please specify)

21. Will a lion bone quota restrict your business in any way?

- ○ Yes
- ○ No
- ○ We are not currently in the lion bone business

22. Since you answered Yes, how will you adapt the business (select all that apply)

- ☐ Will stop selling bones
- ☐ Will continue selling bones, but downscale
- ☐ Search for alternative markets for the bones
- ☐ Close the business
- ☐ Focus on interactive tourism
- ☐ Continue business as usual
- ☐ Other (please specify)

23. In the last two years, has the number of lions euthanised at the facility increased?

- ◯ Yes
- ◯ No
- ◯ N/A

If Yes, indicate when this started and the reasons:

24. Do you think the general trade in captive produced lions (live and products/bones/derivatives) affects wild lion populations? How?

SECTION C: Captive stock numbers

25. By 31 January 2018, approximately how many of the following did you have? Enter a number in each line [including zero (0) if applicable]

Adult males (3+ years)

Adult females (3+ years)

Subadults all sexes (1-3 years)

Cubs (all sexes) (less than 1 year)

26. By 31 January 2017, approximately how many of the following did you have? Enter a number in each line [including zero (0) if applicable]

Adult males (3+ years)

Adult females (3+ years)

Subadults all sexes (1-3 years)

Cubs (all sexes) (less than 1 year)

27. By 31 January 2016, approximately how many of the following did you have?

Adult males (3+ years)

Adult females (3+ years)

Subadult all sexes (1-3 years)

Cubs (all sexes) (less than 1 year)

28. By 31 January 2015, approximately how many of the following did you have?

Adult males (3+ years)

Adult females (3+ years)

Subadult all sexes (1-3 years)

Cubs (all sexes) (less than 1 year)

29. From where has the current lion stock in the facility been sourced: **(select all that apply to your facility)**

- [ ] Lions bred in this facility
- [ ] Lions from breeders in South Africa
- [ ] Lions from breeders elsewhere in Africa
- [ ] Lions from breeders elsewhere
- [ ] Other (please specify)
- [ ] Wild-sourced lions from South Africa
- [ ] Wild-sourced lions from elsewhere in Africa
- [ ] Do not know
- [ ] Other*

30. From where was the original lion stock for the facility sourced: **(select all that apply to your facility)**

- ☐ Lions bred in this facility
- ☐ Lions from breeders in South Africa
- ☐ Lions from breeders elsewhere in Africa
- ☐ Lion breeders elsewhere
- ☐ Circuses
- ☐ Other (please specify)
- ☐ Wild-sourced lions from South Africa
- ☐ Wild-sourced lions from elsewhere in Africa
- ☐ Do not know
- ☐ Other*

Redirect question

*** 31. Is the purpose of this facility for <u>hunting only</u> (i.e. no breeding)**

- ◯ No (redirect to Q32)
- ◯ Yes, the purpose of the facility is for hunting only (redirect to Q45)

Redirect question

*** 32. Does the facility breed lions?**

- ○ Yes, we breed lions (redirect to Q33)
- ○ No, we do not breed lions (redirect to Q39)

PDF version of NATIONAL CAPTIVE LION SURVEY (South Africa) (V2: REVISED FOR 2018)

SECTION D: Lion breeding

33. How many adults (3+ years) are presently being used to breed?

Number of adult males

Number of adult females

34. Indicate the average percentage that these factors result in a reduction in lion numbers at this facility:

Euthanasia

Selling as live animals

Hunting

Natural mortalities

Fights

Poaching

35. How are lion numbers controlled at the facility? (answer all that apply)

- ☐ Euthanising
- ☐ Sterilisation: females
- ☐ Hunting
- ☐ Separation of adults
- ☐ Contraception
- ☐ Natural mortalities
- ☐ Sterilisation: males
- ☐ Selling
- ☐ Fights
- ☐ Other (please specify)

36. Has the facility ever introduced wild lions into its breeding stock?

◯ Yes   ◯ No

If yes, indicate when, where and/or which populations(s) the stock was from

37. Please answer Yes, No, or N/A to the following questions:

	Yes	No	N/A
Does your facility keep a stud book?	◯	◯	◯
Are the lions marked in a manner that they are identifiable (e.g. microchipped, etc)	◯	◯	◯
Have the lions been DNA analysed?	◯	◯	◯

Redirect question

*** 38. Has the facility sold live lions since 2012?**

- ◯ Yes, we have sold live lions (redirect to Q39)
- ◯ No, we have have not sold live lions (redirect to Q45)

SECTION E: Live lion trade

39. Estimate the number of live lions sold from this facility in the specified years:

	2014	2015	2016	2017
To breeding facilities in South Africa				
For hunting purposes in South Africa				
To keeping facilities in South Africa				
To international breeders or keepers				
To international hunting operators (i.e. not in South Africa)				

Other (please specify) and/or elaborate on the international destinations

40. What is the average age that live lions are sold for:

	Male	Female
Hunting		
Breeding		
Bones		

41. In 2015, what was the average sale price of an adult lion:

Males, to SA breeders

Males, for SA keepers

Males, for hunting in SA

Males, to international breeders

Males, to international keepers

Lioness, to SA breeders

Lioness, for SA keepers

Lioness, for hunting

Lionesses, to international breeders

Lionesses, to international keepers

42. In 2016, what was the average sale price of an adult lion:

Males, to SA breeders

Males, for SA keepers

Males, for hunting in SA

Males, to international breeders

Males, to international keepers

Lioness, to SA breeders

Lioness, for SA keepers

Lioness, for hunting

Lionesses, to international breeders

Lionesses, to international keepers

43. In 2017, what was the average sale price of an adult lion:

Males, to SA breeders

Males, for SA keepers

Males, for hunting in SA

Males, to international breeders

Males, to international keepers

Lioness, to SA breeders

Lioness, for SA keepers

Lioness, for hunting

Lionesses, to international breeders

Lionesses, to international keepers

Redirect question

*** 44. Has the facility sold lion products, bones, skeletons, body parts, trophies and/or other derivatives?**

- ◯ Yes, we have sold products (redirect to Q45)
- ◯ No (redirect to Q55)

SECTION F: Trade in lion bones, skeletons, products and derivatives

45. Select the lion body parts the facility is aware of having sold/supplied to:

	Hunters	Taxidermists	Direct to customers in Asia	To SA traders selling to Asian customers	'Muti' traders in South Africa	Customers in other African countries	Other markets (elaborate in comments)
Full trophy (skeleton, skull, skin, teeth, claws)	☐	☐	☐	☐	☐	☐	☐
Skeletons (full/partial)	☐	☐	☐	☐	☐	☐	☐
Skin	☐	☐	☐	☐	☐	☐	☐
Claws	☐	☐	☐	☐	☐	☐	☐
Skull	☐	☐	☐	☐	☐	☐	☐
Teeth	☐	☐	☐	☐	☐	☐	☐
Paws	☐	☐	☐	☐	☐	☐	☐
Individual bones	☐	☐	☐	☐	☐	☐	☐
Fat	☐	☐	☐	☐	☐	☐	☐
Internal organs	☐	☐	☐	☐	☐	☐	☐

Further comments?

46. If the facility has used a middleman (SA and/or foreign) to export bones/skeletons to Asia, what % of the total number of skeletons went via a middleman/bone agent/trader?

0% (no sales from this facility use a middleman/bone agent/trader)

50%

100% (all sales use a middleman/bone agent/trader)

47. If you sell bones through middlemen, have you sold bones using middlemen based in countries besides South Africa? If yes, what percentage of bones are sold through these international middlemen (against the balance sold to South African middlemen)

0% (no sales through middlemen/bone agents/traders based in another country)	50%	100% (all sales use a South African middleman/bone agent/trader)

48. Before January 2016, what percentage of:

...skeletons originating from hunting trophies went into the bone market?

...skeletons originating from natural mortalities went into the bone market?

...skeletons originating from euthanised animals went into the bone market?

49. Indicate the years in which lion bones/skeletons originating from this facility were exported to Asia (that you are aware of):

- ☐ 2008
- ☐ 2009
- ☐ 2010
- ☐ 2011
- ☐ 2012
- ☐ 2013
- ☐ 2014
- ☐ 2015
- ☐ 2016
- ☐ 2017
- ☐ predicted 2018

50. List the prices (or a range) at which you sold<u>MALE</u> lion skeletons (complete set and/or partial): ***(Indicate if this price is paid to the middleman or the Asian importer)***

2012

2013

2014

2015

2016

2017

51. List the prices (or a range) at which you sold LIONESS skeletons (complete set and/or partial). *(Indicate if this price is paid to the middleman or the Asian importer)*

2012	
2013	
2014	
2015	
2016	
2017	

52. Indicate the number of skeletons (i.e. that equate to a whole lion) that made up the bone exports from this facility to Asia each year.

2012	
2013	
2014	
2015	
2016	
2017	

53. Estimate how many skeletons (i.e. that equate to a whole lion) that you could currently export to Asia in one year if there were no restrictions. Please explain how you calculated this estimate.

Redirect question

*** 54. Is hunting allowed on the premises?**

- ◯ Yes, hunting is allowed (redirect to Q55)
- ◯ No, hunting is not allowed (redirect to Final Comments & Suggestions)

PDF version of NATIONAL CAPTIVE LION SURVEY (South Africa) (V2: REVISED FOR 2018)

SECTION G: Lion hunting

55. What is the average length of time that lions have been in the hunting area at the commencement of the hunt?

56. Concerning hunting areas set aside for hunting, list:

How many hunting areas?

Size of hunting area 1

Size of hunting area 2 (if applicable)

Size of hunting area 3 (if applicable)

Number of lions currently in hunting area 1?

Number of lions in hunting area 2? (if applicable)

Number of lions in hunting area 3? (if applicable)

57. <u>Before</u> January 2016, indicate the percentage of clients who were from:

South Africa

USA

United Kingdom

Europe

Middle-East

Australia

Canada

Asia

Other

58. **<u>After</u> January 2016, indicate the percentage of clients who were from:**

South Africa

USA

United Kingdom

Europe

Middle-East

Australia

Canada

Asia

Other

59. **How many lions were hunted on the property in:**

2012

2013

2014

2015

2016

2017

60. **After January 2016: if your income from lion hunting started to decline, what month did your business start to experience this decline:**

61. Since January 2016, what do you estimate the total loss of earnings to be? Explain how you arrived at this figure

62. Due to the ban (from January 2016), how many people have you retrenched and/or been unable to support?

Skilled workers

Unskilled workers

Professional hunters

Other 1 (describe)

Other 2 (describe)

63. Any comments and suggestions?
