# Supplementary material for: Born captive: A survey of the lion breeding, keeping and hunting industries in South Africa
Source: PLoS One. 2019 May 28;14(5):e0217409. doi: 10.1371/journal.pone.0217409 (PMC6538166; doi:10.1371/journal.pone.0217409)
Supplement: S2 File — Sections, questions numbers, questions, and the number of responses per question. (PDF) [file pone.0217409.s004.pdf]

S2 FILE

QUESTION SUMMARIES: NUMBER OF RESPONDENTS AND ANSWER LOCATION

Abbreviations:

- **X** = not in the manuscript;
- **R** = in results/text of the manuscript;
- **Tables/Figs** = in the numbered Tables and Figures;
- **S1–S6 Files/Figures/Tables** = in the numbered Supplementary Files/Figures/Tables

Colours:

- Beige answers in the manuscript and SANBI report;
- White answers not in the manuscript or SANBI report;
- Blue answers in the SANBI report only.
- Red answers excluded and will be part of a separate *in prep* paper.

Section	Question no.	Question	Respondent count per question	Answer location
A	Q1	(Preference for anonymity) (*number of respondents that viewed the survey)	242*	R
	Q2	Name	66	X
	Q3	Province	197	Table 1
	Q4	Job description	197	R
B	Q5	Year facility opened	117	R; S1 Figs (Figure A)
	Q6	Membership of organisations	108	R; S1 Tables (Table B)
	Q7	Facility purpose	107	R
	Q8	No. of employees	107	R; S1 Figs (Figure B)
	Q9	Reasons for breeding	107	R; S1 Figs (Figure C); S1 Tables (Table C)
	Q10	Reasons for keeping	107	
	Q11	Rank the core purposes of the facility	105	R; Fig. 2
	Q12	No. of paying visitors, and are the numbers increasing/decreasing/stable?	97	X
	Q13	Approximate sizes of various areas (farm, breeding, keeping, camps, etc)	102	R; Table 2; S1 Tables (Table G)
	Q14	From January 2016, which ones increased/decreased/stayed the same (refers to no. of lions on the property, total breeding areas, total keeping/growing area, total hunting area)	105	R; S1 Figs (Figure E)
	Q15 & Q16	Est. annual value of sales 1 & 2 (8 income categories)	73	R; Fig 3; S1 Figs (Figure D); S1 Tables (Table K)
	Q17	Any impact if 2016 US decision?	105	R; S3 File
	Q18	If yes to (17), indicate how you are adapting?	86	
	Q19	What will happen if US ban continues?	106	
	Q20	If the UK & Europe implement bans in the future, what will you do?	105	
	Q21	Will the lion bone quota restrict business?	107	R
	Q22	If yes to (21), how will you adapt?	107	R
	Q23	Has euthanasia of lions increased in 2 years	105	R; S4 file
	Q24	Does trade of captive lions affect wild lion populations (describe how)	97	X

**C**	Q25	Est. no. lions at 31 January 2018	64	R; Table 4; S1 Tables (Tables H–J)
	Q26	Est. no. lions at 31 January 2017	84	
	Q27	Est. no. lions at 31 January 2016	70	
	Q28	Est. no. lions at 31 January 2015	59	
	Q29	From where has current lion stock been sourced?	100	R; S1 Tables (Table F)
	Q30	Where was original lion stock sourced from?	100	
	Q31	**Redirect: is purpose hunting only? (No→ Q32; Yes→ Q44)**	102	X
	Q32	**Redirect: does facility breed lions (Yes→ Q33; No→ Q38)**	82	X
**D**	Q33	No. adults breeding	67	R; S1 Tables (Table D)
	Q34	% that certain factors result in reduction in lion numbers	64 (partial)	X (ambiguous question wording)
	Q35	How are lion numbers controlled?	71	R; S1 Tables (Table E)
	Q36	Wild lions introduced to breeding stock?	71	R
	Q37	Stud book, identification, DNA?	71	X
	Q38	**Redirect: has facility sold live lions (Yes→ Q39; No→Q44)**	81	X
**E**	Q39	Est. no. live lions sold 2014 - present	55	R; Fig 4; S1 Figs (Figure G & H)
	Q40	Average age when sold	57	R; S1 Figs (Figure F)
	Q41 to Q43	Average sale price adult lion 2015, 2016 and 2017	46	R; Fig 5; S1 Figs (Figures I–K); S1 Tables (Tables L & M)
	Q44	**Redirect: facility sold products, bones, trophies etc (Yes→Q45; No→Q54)**	98	X
**F**	Q45	Have products been sold to any of the seven customer types	52	R; Fig 6
	Q46	Has facility used middleman to export bones to Asia	98	R
	Q47	Has facility sold bones via middlemen in other countries	98	R
	Q48	Before Jan 2016, what % skeletons from named selected sources entered bone market	47	R; S1 Figs (Figures L & M)
	Q49	When did bone exports from facility start?	45	R; Fig 7; Fig 8; S1 Figs (Figure N)
	Q50	Prices of male skeletons 2012-2017	35	R; Fig 9; S1 Figs (Figures O–T)
	Q51	Prices of female skeletons 2012-2017	35	
	Q52	Number skeletons exported 2012-2017	31	R; Fig 10; S1 Figs (Figure U); S1 Tables (Table N)
	Q53	How many skeletons could you export?	31	
	Q54	**Redirect: hunting allowed at facility? (Yes→ Q55; No→ Q63)**	106	X
**G**	Q55	Ave. time lion in hunting area	25	R
	Q56	Details of hunting area(s) (sizes, number, etc)	28	R; Table 3
	Q57	% clients from countries before 2016	33	R; S1 Tables (Table O)
	Q58	% clients from countries after 2016	33	
	Q59	Number lions hunted 2012-2017	22	R; Fig 11
	Q60	After Jan 2016, what month did income start to decline?	27	X
	Q61	Since Jan 2017: est. loss of earnings	90	R; case studies in S6 File
	Q62	Due to ban, have people been retrenched?	27	
	Q63	Final comments and/or suggestions	28	R
