# Supplementary material for: Born captive: A survey of the lion breeding, keeping and hunting industries in South Africa
Source: PLoS One. 2019 May 28;14(5):e0217409. doi: 10.1371/journal.pone.0217409 (PMC6538166; doi:10.1371/journal.pone.0217409)
Supplement: S3 File — Results correspond to Questions 17–20. (PDF) [file pone.0217409.s005.pdf]

S3 FILE

BANS ON IMPORTS OF CAPTIVE-PRODUCED LION TROPHIES: GENERAL IMPACTS AND ADAPTATIONS

Responses to Questions 17–20 are listed below.

- ***Did the January 2016 US ban on the import of captive produced lion trophies impact business in anyway? (Q17)***

  - 105 facilities responded:
    - 86 (82%) indicated that the 2016 US ban had affected business;
    - 8 (8%) indicated the ban had no affect
    - 11 (10%) indicated this was 'Not Applicable' to their business (although this answer is tantamount to 'No' because facilities not in the hunting and breeding sectors typically responded this way)

- ***If the answer was 'Yes' to the above question, respondents were asked to indicate how they were adapting to the impact (Q18)***

  - 86 facilities responded:
    - 54 (63%): breeding production scaled down
    - 50 (58%): employees/workers let go
    - 44 (51%): live lion stock sold off
    - 26 (30%): redirected business to focus on the lion bone trade
    - 22 (26%): euthanized lions
    - 9 (10%): continued business as usual
    - 4 (5%): redirected business to focus on interactive tourism
    - 15 (17%): selected 'other', and listed the following ways in which they were adapting:
      - *We have shifted our focus towards non-export hunts*
      - *We have redirected our marketing of hunting*
      - *We have hunted 35% less lions*
      - *We have 'stockpiled' some adult lions*
      - *Scaling down of number of lions released for hunting purposes*
      - *I bought a hunting farm in 2017* [Note: this was done so that respondent could supply lions from his breeding facility to his new hunting facility and thus avoid euthanizing lions and having to rely on other hunting facilities to buy lions from his breeding facility following the downturn of the market]
      - *Had to let go of lions because the hunting market was weak. No income to look after the animals*
      - *We have scaled down more than half of the hunting area, and paid off 15 workers*
      - *We will start selling again from this year*
      - *Change the business with more focus on buffalo, sable and roan hunts*
      - *Extensive system begins with managed wild lions*
      - *Must sell lions for MUCH less than they are worth – just to keep going and pay expenses*
      - *No interested buyers at the lion sales. Value of animals has come down.*
      - *Had to start over marketing in other countries. Many workers lost their jobs.*

- ***If the US ban continues to be implemented with no sign that it will be lifted in the near future, what would respondents do? (Q19)***

  - 106 facilities responded:
    - 53 (45%): will focus on the lion bone trade
    - 41 (35%): will convert business to another form of wildlife breeding
    - 24 (21%): will close the business
    - 20 (17%): will euthanize all lion stock
    - 19 (16%): will continue business as usual
    - 9 (8%): will focus on interactive tourism
    - 10 (9%): selected ‘other’, and listed the following ways in which they were adapting:
      - *Will adapt business to current markets*
      - *Will look for alternative markets*
      - *Focus on other hunting markets*
      - *Stop breeding and sterilize animals*
      - *Sterilize females*
      - *Enter the hunting market* [response from a breeding-only facility]
      - *Marketing of lion hunts to move to other countries*
      - *Will continue extensively with fewer lions with high worth*
      - *Still unsure what we will do*
      - *What do I do with the facilities that cost me thousands to erect?*

- ***In addition to the US ban, IF the UK and/or Europe also implemented bans on the import of lion hunting trophies, what would the respondents do? (Q20)***

  - 105 facilities responded:
    - 41 respondents (35%): will focus on the lion bone trade
    - 37 (32%): will downscale but continue production expecting that the ban will be lifted
    - 33 (28%): will convert business to another form of wildlife breeding
    - 28 (24%): will close their business
    - 21 (18%): will euthanize all lion stock
    - 17 (15%): will continue business as usual
    - 12 (10%): will focus on interactive tourism
    - 7 (6%): selected ‘other’, and listed the following
      - *Focus on other hunting markets*
      - *Hunt lions with local hunters and continue to sell bones*
      - *Still unsure what to do*
      - *Because I have access to food for lion farming and capital has been spent on building cages and to create jobs and* (the facility) *is already established, it would make sense to continue farming and give the market what it needs to protect the wild* (lion) *population. Because I never did trophy hunting on my premises, it is only a result of the industry that there is still a need for bones. There will always be hunters who are willing to hunt lions and the need for bones will be there, legal or illegal*
      - *In this case, we will be affected because we would lose the interactive lion program*
      - *Release the lions and let them run free.*
