# Supplementary material for: Born captive: A survey of the lion breeding, keeping and hunting industries in South Africa
Source: PLoS One. 2019 May 28;14(5):e0217409. doi: 10.1371/journal.pone.0217409 (PMC6538166; doi:10.1371/journal.pone.0217409)
Supplement: S4 File — Responses to Question 23. (PDF) [file pone.0217409.s006.pdf]

S4 FILE

Lion Euthanasia

Responses to Question 23 are listed below.

In response to the question: *"in the past two years, has the number of lions euthanized at the facility increased?"*, 30 replied 'Yes'. A further 18 elaborated on when and why they started euthanizing lions, and the table lists these open-ended answers.

English answers

- *2017. Lions broke out of the facility.*
- *Middle of 2016, I could not sell excess lions for hunting purposes*
- *When I could not hunt all my access animals.*
- *Females were euthanized, due to old age*
- *Had to euthanize old females*
- *2016 Middle, I could not sell my excess lions for hunting purposes*
- *We expanded* (be)*cause of US closed for hunting*

Afrikaans answers, and English translations

Afrikaans	English
2018 Swak verkope aan jag mark. Swak verkooe weens verbod	2018 Poor sales to hunting market due to prohibition
2018 swak virkope aan VSA, kliente weens die virbot op jag trovee invoere	2018 poor sales to USA clients due to prohibition on hunting trophy imports
2016 Waarde so geval aspok vraag na trofees	Post-2016 fallen values and demand for trophies
agv inkomste verloor deur minder of geen meer verkope aan leeujag ondernrmings, om nie werknemers aftedank nie,om leeugetalle binne perke te hou,om nog steeds n inkomste uit leeus te verdien sodat ek my verpligtinge kan nakom	Due to income lost by reduced or terminated sales to lion-hunting operations, to avoid retrenchment of employees, to maintain my lions within acceptable standards, to still earn an income from lions so that I can fulfill my obligations
As gevolg van die verbod op invoere na Amerika	Due to the ban on imports into America
Volwasse mannetjies vanaf 2016 wat nie gejag kan word nie.	Mature males from 2016 who can not be hunted.
2016 Daar was geen mark vir die leeus nie en die diere het teveel geraak om te onderhou.	2016 There was no market for the lions and the animals became too much to maintain.
wyfies wat nie meer teel nie en wat beseer word indie hulle baklei	Females who no longer breed and who are injured in fighting them
Moet n inkomste uit leeus verkry aangesien jag en verkope afgeneem het en ook kyk ek meer na die teeling van beter gene en so verminder ek enige leeus met slegte gene	Must get income from lions as hunting and sales have decreased and I also look more at the breeding of better genes and thus I reduce any lions with bad genes
Nadat die verbond op die invoer van leeus ingestel het moes ons drasties optree. Op die stadium voer ons nog die leeus maar ons kan nie aangaan nie. Indien die jag nie oopmaak nie sal ons alle leeus laat uitsit	After the ban had been introduced on imports of lion trophies we had to act drastically. At this stage we still carry the lions but we can not proceed. If the hunt does not open, we will 'let the lions go' (euthanize them)
As gevolg van omstandighede.	Due to circumstances.

Three respondents who did not answer 'Yes' provided the following comments

**Has the no. lions euthanised increased?**	**Afrikaans**	**English**
No	Teeling ingeperk	Breeding restricted
N/A	By ons fasiliteite self word daar nie leeus uitgesit nie, maar die kopers van lewendige leeus mag dit moontlik uitgesit het na dit aangekoop is by ons.	We did not euthanize any lions, but buyers of our live lions may have euthanized them after purchase.
N/A	We were still building on our numbers.	
