# Supplementary material for: Born captive: A survey of the lion breeding, keeping and hunting industries in South Africa
Source: PLoS One. 2019 May 28;14(5):e0217409. doi: 10.1371/journal.pone.0217409 (PMC6538166; doi:10.1371/journal.pone.0217409)
Supplement: S5 File — Includes responses to Questions 61 and 62. (PDF) [file pone.0217409.s007.pdf]

S5 FILE

TWO CASE STUDIES DESCRIBING SPECIFIC LOSS OF EARNINGS AND JOBS IN THE NORTH WEST AND FREE STATE PROVINCES

Includes responses to Questions 61 and 62.

CASE 1: Detailed loss of earnings from a facility in the North West province

- Facility estimated to have lost R11 670 000 since January 2016
- Opened: 2009
- Core purpose: hunting safaris
- Other activities: breeding, keeping and live sales
- Size: 1500 ha (4 ha for breeding and keeping; remainder for hunting)
- Employs: 11 people
- Retrenched since January 2016: 6 unskilled workers
- The farm also caters for plains game hunts. Hence, when lion hunts were cancelled, the hunter also cancelled the entire hunting package.
- The facility calculated the "*very conservative*" loss of earnings to be the following:
    - Loss from cancellation of lion hunts: R10 610 000
        - 2016: 30 x male lions with average price of R150 000 = R4 500 000
        - 2016: 10 x lionesses with average price of R45 000 = R450 000
        - 2017: 30 x male lions with average price of R150 000 = R4 500 000
        - 2017: 10 x lionesses with average price of R45 000 = R450 000
        - 2016/7: stay of R1250 x 2 clients per hunt (80 clients x R1 250) = R100 000
        - 2016/7: stay of R750 x 1 PH (Professional Hunter) per hunt (80PH x R750) = R60 000
    - Loss from other game hunts following cancellation of lion hunt: R1 610 000
        - 40 x blue wildebeest = R220 000
        - 20 x kudu = R250 000
        - 40 x blesbok = R140 000
        - 20 x impala = R50 000 (impala)
        - 5 x buffalo = R500 000
        - 10 x sable = R450 000

CASE 2: Description of loss of earnings and jobs from a facility in the Free State province

- Facility estimated to have lost R20 million since January 2016
- Opened: 2012
- Core purpose: Lions as game farm animals, to be used for hunting and ecotourism
- Other activities: hunting, breeding, and ecotourism
- Size: 7400 ha (5700 ha set aside for hunting)
- Employs: 15 people
- Retrenched since January 2016: 13 unskilled workers (each with 6 dependents), and 3 skilled workers (each with 4 dependents), and 3 Professional Hunters
- Comments on loss of income: "*from lion hunts that can't be imported into the USA and the plains game that the lion hunters and their hunting parties would shoot at the same time*".
