# Supplementary material for: Born captive: A survey of the lion breeding, keeping and hunting industries in South Africa
Source: PLoS One. 2019 May 28;14(5):e0217409. doi: 10.1371/journal.pone.0217409 (PMC6538166; doi:10.1371/journal.pone.0217409)
Supplement: S6 File — Responses to Question 63. (PDF) [file pone.0217409.s008.pdf]

# S5 File

# Final Respondent Comments

## Responses to Question 63 are listed below.

The following responses are in answer to the question: *“Any comments or suggestions?”*; 28 replied. No summaries of the responses have been produced.

| **a) English** |
|---|
| Hunting of lions is the priority and then the bone market supporting. |
| The hunting of lions is the main focus with lion bones as a supportive industry. |
| I would like to continue hunting lions, and use the products from the hunted lions to export in the lion bone trade market. |
| The lion bone trade is a supportive industry to the breeding and keeping of lions. If we cannot hunt lions in order to generate and income, we will need to increase the bone trade market. |
| I love my lions. Unfortunately, if I cannot hunt my lions, I cannot afford to maintain them in my facilities. I have to sell all my lions in order to cover financial losses due to the USA Ban. It was not possible for me to sell my lions to the hunting market. |
| The hunting of lions is a commercial venture. The end value of the lions are important, as this motivates me to maintain these animals in the expensive facilities. The cost involved to establish and maintain facilities for lion hunting are very high. |
| We are a high class tourism destination and are keeping our lions for tourism purposes. |
| We are in contract research involved with South African and International Universities. Primarily focusing on reproductive genetics. |
| I have got a large breeding and keeping facility. In order for me to maintain this facility and provide quality care of my animals, I would need to secure an income of some sort. Previously this income was mostly from the sales of live animals for the hunting market. Due to the USA ban, I could not sell all my adult animals to the hunting market, this influenced my management system and I was forced to euthanize animals in order to ensure enough space in my keeping facilities. |
| I am having a small predator facility. But would need to "exchange" my male with another male. This old male of mine will then needs to be either hunted or euthanized in order to cover for the expenses in order to keep the animals. |
| I need to ensure a market for the my lions. If no market exists I will not be able to afford the cost involved in keeping these animals and would thus be forced to either sell my animals or euthanize them all. |
| The Breeding of lions for hunting purposes are my major focus. Conservation of the Species are very important to me. The lion bone trade is a supplementary business to my major Hunting business. |
| Hunting of lions are very important for the lion industry in South Africa and for the Conservation of the lion as a species in Africa |
| A controlled bone market is better, than an uncontrolled illegal market. |
| This hunting venture was started as a expansion of the breeding project, to ensure a product flow. |
| CBL industry needs to change to be more "free-roaming" oriented |
| No quota and lions will thrive. |
| I need to have a market for my lion. |

| **b) Afrikaans** | **English translation** |
|---|---|
| Maak invoere na vsa oop. As dit nie spoedig gebeur nie gaan daar binnekort niks oorwees nie. Leeus sal dieselfde pad as renosters loop. As daar nie n inkomste is om na jou leeus om te sien nie sal hulle nie na gekyk kan word nie. Dit kos geld om leeus te he!!! If it doesn't pay, it goes!!! | Open the imports to the USA. If it does not happen soon, soon nothing will be left. Lions will go the same way as rhinos. Without income, you will not be able to look after your lions. It costs money to keep lions!!! If it doesn't pay, it goes!!! |
| Die virbot op die invoer van jag trovees in die vsa. Het n grood inpak op die leeu populasie in SA wat as buffer dien tussen wilde en hok leeus, sou die virbot langer duur sal die aanslag op wilde leeus sterker word en sal die leeu getalle is siud afrika ook afneem soos die ander afrika lande. | The ban on the import of hunting trophies in the USA had a big impact of the lion population in South Africa, which is a buffer between wild and captive lions. If the ban lasts longer, then the onslaught on wild lions will be stronger and the numbers in South Africa will drop as has happened in other African countries. |
| Gebruik gesonde verstand. Vat enige dier se waarde weg en vernietig hom | Use your common sense. If you remove an animal's value you will destroy it. |
| Stel 'n kwota vas vir uitvoere van byprodukte slegs vir leeus wat gejag is. Nie vir leeus wat uitgesit is nie. | Establish a quota only for lions that are hunted, not for those that are euthanized. |
| Die leeubedryf beskerm nie alleen die wilde leeus nie dit skep werk toerims buitelandse valuta ekonomiese groei ekonomiese welvaart vir alle suid afrikaners | The lion industry does not only protect wild lions, it creates job opportunities, internationally driven economic growth and welfare for all South Africans. |
| Ek is n leeu boer en taxidermy ek is n middelman tusen die boer en client in die been bedryf my voorstel is om die kwota 1600–2000 karkaste n jaar temaak ek glo ons kan soveel jaarliks verkoop sonder om die aantal leeus teverminder | I am a lion farmer, taxidermist and intermediary between other farmers and clients in the bone industry. My proposal is to set the quota at 1600–2000 carcasses per year. I believe this is the sustainable annual production capacity of the industry. |
| Leeus Moët in boere se hande Gelos word wat Sapa monitor in same werking met regering ! Suid Afrika soek werkskepping en hoe meer ons hulle Kan uitvoer vir bene hoe meer werkskep ons ! Werkskepping is meer belangrik as as " anti Leeu Jagters/ anti teel / of anti hok! | Lions must be placed in the hands of farmers affiliated to SAPA and working together with the government! South Africa seeks job creation, and the more we can export the more jobs we can create. Employment is more important than those that are opposed to hunting, breeding or keeping. |
| Met huidige kwota stelsel, sukkel met permitte en jagmark sal genoodsaak wees om besigheid drasties kleiner te maak. Voorstel is dat geen kwota op uitvoere moet wees. Ek wil teel met leeus soos skape en beeste. Natuurlik sal ek bewaring toepas en dan teel vir beter genetika. Eie genetika is klaar beter as wilde leeus in parke. Teveel mense het eier te lê oor die besigheid wat geen kennis daarvan het nie. Natuurbewaring kan hul toepas op wilde leeus en parke i.p.v ons telers wil agtervolg i.p.v. om ons te wil help. Selfde geld vir SPCA. Permitte is n gemors. Maak 1 genasionaliseerde permit stelsel oor hele land. Bv. FS het hul siening en NW hul eie maar voer nou uit v 1 provinsie na ander. Hul werk nie saam nie. In natuurbewaring is daar individue wat eie besluite neem hoe dit moet werk. Kom daarop neer onbevoegde mense word in poste gestel wat geen kennis van bedryf het nie. Ons word verhoed om ons besigheid tot sy potensiaal te bedryf. Solank enige dier n waarde het sal bewaring toegepas word. Om bewaring toe te pas het jy geld nodig en om geld te kry moet jy produk kan verkoop. | With the current quota system, struggling with permits and hunting markets, it will be necessary to make the business drastically smaller. My proposal is that there should be no quota on exports. I want to breed with lions like sheep and cattle. Of course I will apply conservation principles and then breed for better genetics. Our genetics are already better than wild lions in parks. Too many people are critical of a business of which they have no knowledge. Nature conservation can look after wild lions and parks instead of pursuing breeders and not assisting us. The same applies to the SPCA. Permits are a mess. Create one nationalized permit system across the country. Approaches vary between provinces (e.g. Free State and North West) and they do not work together. In nature conservation there are individuals who make their own decisions on how to work. Incompetent people, with no knowledge of our business, are appointed and prevent us from operating our business to its potential. As long as any animal has a value, it will be protected. To conserve you need money and to get money you need to sell a product. |
| Been mark moet toegelaat word tot jag weer op dreef kom | The bone market must be allowed until hunting market recovers |
